# Microglia‐synapse engulfment via PtdSer‐TREM2 ameliorates neuronal hyperactivity in Alzheimer's disease models

**DOI:** 10.15252/embj.2022113246

**Published:** 2023-08-14

**Authors:** Javier Rueda‐Carrasco, Dimitra Sokolova, Sang‐Eun Lee, Thomas Childs, Natália Jurčáková, Gerard Crowley, Sebastiaan De Schepper, Judy Z Ge, Joanne I Lachica, Christina E Toomey, Oliver J Freeman, John Hardy, Samuel J Barnes, Tammaryn Lashley, Beth Stevens, Sunghoe Chang, Soyon Hong

**Affiliations:** ^1^ UK Dementia Research Institute, Institute of Neurology University College London London UK; ^2^ Neuroscience BioPharmaceuticals R&D, AstraZeneca Cambridge UK; ^3^ Department of Physiology and Biomedical Sciences Seoul National University College of Medicine Seoul South Korea; ^4^ Department of Neuroscience, Physiology and Pharmacology University College London London UK; ^5^ The Queen Square Brain Bank for Neurological Disorders UCL Queen Square Institute of Neurology London UK; ^6^ Department of Clinical and Movement Neurosciences UCL Queen Square Institute of Neurology London UK; ^7^ UK Dementia Research Institute, Department of Brain Sciences Imperial College London London UK; ^8^ Department of Neurodegenerative Diseases UCL Queen Square Institute of Neurology London UK; ^9^ F.M. Kirby Neurobiology Center Boston Children's Hospital Boston MA USA; ^10^ Harvard Medical School Boston MA USA; ^11^ Stanley Center for Psychiatric Research Broad Institute of MIT and Harvard Cambridge MA USA; ^12^ Howard Hughes Medical Institute, Boston Children's Hospital Boston MA USA

**Keywords:** Abeta oligomers, Alzheimer's disease, microglia, pruning, synapses, Immunology, Neuroscience

## Abstract

Neuronal hyperactivity is a key feature of early stages of Alzheimer's disease (AD). Genetic studies in AD support that microglia act as potential cellular drivers of disease risk, but the molecular determinants of microglia‐synapse engulfment associated with neuronal hyperactivity in AD are unclear. Here, using super‐resolution microscopy, 3D‐live imaging of co‐cultures, and *in vivo* imaging of lipids in genetic models, we found that spines become hyperactive upon Aβ oligomer stimulation and externalize phosphatidylserine (ePtdSer), a canonical “eat‐me” signal. These apoptotic‐like spines are targeted by microglia for engulfment via TREM2 leading to amelioration of Aβ oligomer‐induced synaptic hyperactivity. We also show the *in vivo* relevance of ePtdSer‐TREM2 signaling in microglia‐synapse engulfment in the hAPP NL‐F knock‐in mouse model of AD. Higher levels of apoptotic‐like synapses in mice as well as humans that carry TREM2 loss‐of‐function variants were also observed. Our work supports that microglia remove hyperactive ePtdSer^+^ synapses in Aβ‐relevant context and suggest a potential beneficial role for microglia in the earliest stages of AD.

## Graphical abstract

Microglia remove hyperactive phosphatidylserine‐positive synapses via TREM2 in Alzheimer's disease (AD) models supporting a beneficial role for microglia in AD.

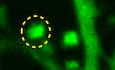

## Introduction

A key function of tissue‐resident macrophages is to phagocytose various elements in their local milieu to help maintain tissue homeostasis (Doran *et al*, 2020). In the healthy brain, microglia respond to changes in local neuronal activity and engulf certain synaptic elements contributing to synaptic refinement (Badimon *et al*, 2020; De Schepper *et al*, 2021). In the aged, diseased, or injured brain, microglia are often found phagocytosing toxic aggregates, including β‐amyloid (Aβ) plaques, dying cells and debris (Podleśny‐Drabiniok *et al*, 2020). Interestingly, in various models of neurologic diseases, including those of AD, we and others have shown that microglia‐mediated developmental synaptic pruning pathway involving complement (Stevens *et al*, 2007; Schafer *et al*, 2012) is reactivated in a region‐specific manner to mediate synaptic dysfunction and loss (Hong *et al*, 2016; Lui *et al*, 2016; Shi *et al*, 2017; Dejanovic *et al*, 2018; Vukojicic *et al*, 2019; Wu *et al*, 2019; Gratuze *et al*, 2020; Werneburg *et al*, 2020). Specifically, in animal models of amyloidosis, microglia‐synapse engulfment occurs early, before robust plaque deposition and plaque‐associated microglia population, as Aβ oligomers accumulate and synapses become lost (Hong *et al*, 2016). However, we still do not know whether specific synapses are targeted for engulfment by microglia in this early AD‐relevant context, and if so, which ones are eliminated (Bartels *et al*, 2020). Addressing this question is important because it will help explain whether synaptic engulfment by microglia is, at least at these early stages, protective or pathogenic, and will provide insight into how microglial function could be targeted in disease.

A fundamental role of macrophages is to selectively remove unwanted materials in their local microenvironment for proper maintenance of tissue homeostasis (Lemke, 2019). A conserved mechanism governing the selectivity of macrophage removal involves the recognition of externalized phosphatidylserine (ePtdSer). PtdSer is normally asymmetrically localized to the inner leaflet of plasma membranes; however, it can be externalized to the outer leaflet during caspase‐mediated apoptosis for the removal by phagocytes (Segawa *et al*, 2014). Caspase‐3 activation and PtdSer externalization can occur locally on isolated cell membranes in the absence of overt cell death (Ertürk *et al*, 2014; Scott‐Hewitt *et al*, 2020). Microglia express a plethora of receptors that recognize ePtdSer including AD‐risk‐associated triggering receptor expressed on myeloid cells 2 (TREM2; Wang *et al*, 2015). Here, we hypothesized that local synaptic ePtdSer underlies the vulnerability of synapses to microglial engulfment via TREM2.

We show in Aβ models, which replicate the earliest phases of disease pathogenesis when synapses become dysfunctional (Li *et al*, 2009; Zott *et al*, 2019), which microglia display preferential selectivity toward “damaged” synapses, that is, those which externalize PtdSer on their membranes. Elimination of ePtdSer^+^ synapses via TREM2 results in the resolution of Aβ oligomer‐induced neuronal hyperactivity. Furthermore, in TREM2 dysfunctional microglia where recognition of ePtdSer is impaired (Wang *et al*, 2015), microglia no longer preferentially engulf Aβ^+^ synapses *in vitro* despite normal phagocytic activity, a finding which was further corroborated with *in vivo* microglia‐synapse engulfment assay in hAPP NL‐F KI;Trem2 R47H KI mice. In accord, we find significantly higher levels of uncleared apoptotic‐like synapses in mouse and human brains of patients with TREM2 loss‐of‐function mutations. Altogether, our data suggest that microglia‐synapse engulfment is, at least in the beginning, a beneficial mechanism targeted toward ePtdSer^+^ synapses with the goal of resolving neuronal homeostasis.

## Results

### Microglia show preferential selectivity toward Aβ^+^ ePtdSer^+^ synapses

**Figure 1:**
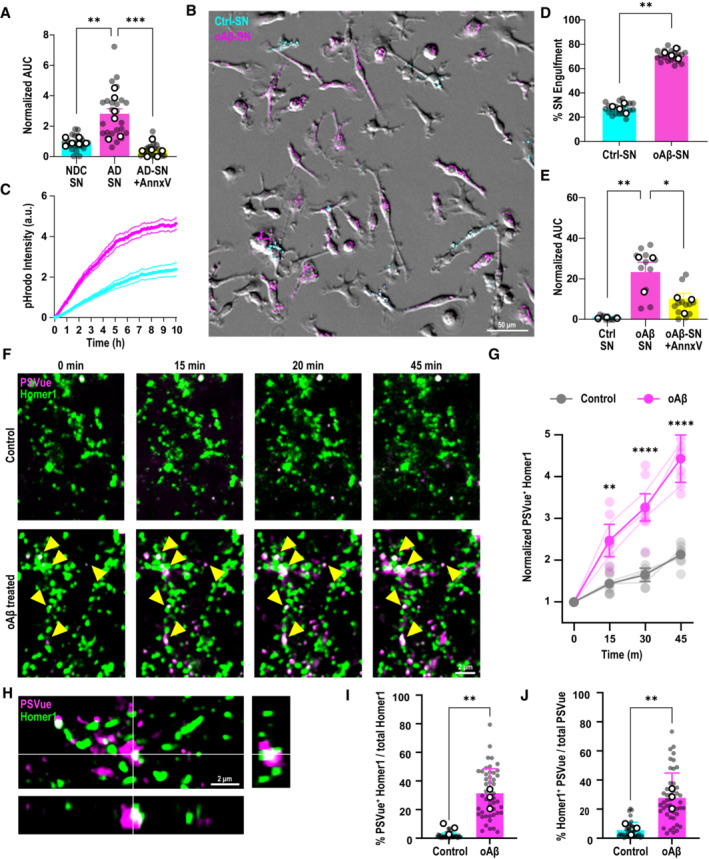
Microglia target AD synapses for engulfment via ePtdSer AArea under curve (AUC) for pHrodo fluorescence signals in primary microglia at *t* = 3 h, normalized to respective control, post‐application of synaptosomes (SN) from either AD patients or NDC brains, with and without Annexin‐V (AnnxV) pretreatment. Data are normalized to NDC SN. ∼40 microglia per ROI, two ROIs per well, 2–3 wells per experiment, *n* = 6 human cases for NDC and AD, *n* = 3 independent experiments.BRepresentative image of primary microglia simultaneously treated with Aβ oligomer‐bound SN (oAβ‐SN; in pHrodo red [magenta]) and control SN (Ctrl‐SN; in pHrodo deep red [cyan]). Scale bar, 50 μm.CpHrodo fluorescence (a.u.) over 10 h (3–5‐min intervals, SNs added to microglia at *t* = 0) showing a faster rate of increase of oAβ‐SN compared with Ctrl‐SN. Data are normalized to Ctrl‐SN. ∼40 microglia per ROI, two ROIs per well, 2–3 wells per experiment, *n* = 4 independent experiments.DPercentage of pHrodo fluorescence of either oAβ‐SN or Ctrl‐SN in microglia at *t* = 3 h. ∼40 microglia per ROI, two ROIs per well, 2–3 wells per experiment, *n* = 3 independent experiments.EpHrodo fluorescence with time shown as AUC at 3 h normalized to respective control. AUC of engulfed mouse oAβ‐SN versus Ctrl‐SN with and without AnnxV pretreatment.FTime‐lapse images of primary Homer1‐eGFP neurons (green) treated with 50 nM oAβ versus vehicle control. Yellow arrows indicate increasing PSVue550 (magenta) signal on dendritic spines with Aβ oligomer treatment over 45 min. Scale bar, 2 μm.GRelative fold change of colocalized PSVue and Homer1‐eGFP signal over time. Two–three ROIs per experiment, *n* = 3 independent experiments.HOrthogonal view of AiryScan image showing colocalization of Homer1‐eGFP (green) and PSVue (magenta) at 1‐h post‐treatment with Aβ oligomer. Scale bar, 2 μm.IQuantification of colocalized Homer1‐eGFP with PSVue among total Homer1. Three ROIs per neuron, 4–5 neurons, *n* = 3 independent experiments.JQuantification of colocalized PSVue with Homer1‐eGFP among total PSVue. Three ROIs per neuron, 4–5 neurons, *n* = 3 independent experiments. Data information: Data shown as mean ± SEM (A–E) or as mean ± SD (F–J). Each shaded point represents one ROI, and each open point represents the mean of each independent experiment (A, D, E, I, J). Filled point (G) represents the average of all ROIs. One‐way (A and E) or two‐way (G) ANOVA followed by Bonferroni *post hoc* test, paired (D) or unpaired (I and J) *t*‐test. *P*‐values shown as *P < 0.05; **P < 0.01; ***P < 0.001; ****P < 0.0001. Source data are available online for this figure.

**Figure EV1:**
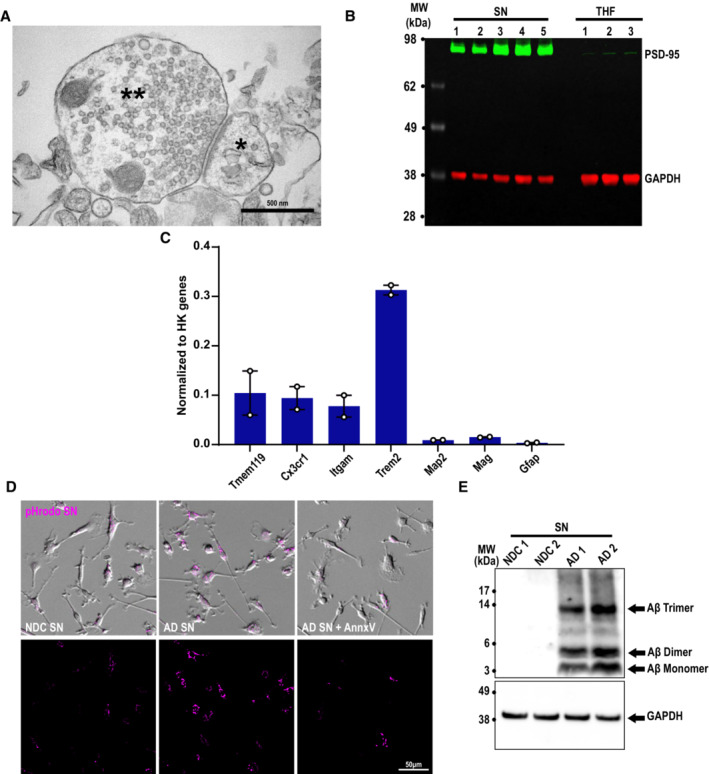
Annexin‐V pretreatment of AD synaptosomes decreases microglial engulfment ACrude synaptosomes were prepared using a protocol that yields electrically functional synaptosomes for several hours post‐isolation, which can be depolarized and stimulated with KCl and NMDA, respectively. Electron microscopy of crude synaptosome preparation showing intact pre‐ (**) and postsynaptic sites (*). Scale bar 500 nm.BWestern blot showing enrichment of synaptic markers PSD‐95 (green, 95 kDa) in synaptosomes (SN) compared with total homogenate (THF) fractions with respect to GAPDH (red, 37 kDa) loading control. One lane represents one mouse.CPrimary microglia grown with TGFβ express high mRNA levels of microglial genes including *Cx3cr1*, *Itgam*, *Trem2* but also homeostatic *Tmem119* with little contamination from neurons (*Map2*), astrocytes (*Gfap*), and oligodendrocytes (*Mag*). Gene expression normalized to the geomean of three housekeeping genes (*Actb*, *Gapdh*, and *Rpl32*).DMicroglia treated with NDC (NDC SN), AD (AD SN), or AD synaptosomes pretreated with 10 μg/ml Annexin‐V (AD SN + AnnxV) conjugated to pHrodo red (magenta). Scale bar 50 μm.EAll isolated human synaptosomes (SN) used in this study have been tested for the presence of Aβ oligomers by western blotting. Exemplar western blot showing higher levels of Aβ trimer (14 kDa), dimer (6.5 kDa), and monomer (3 kDa) in synaptosomes prepared from the frontal cortex of AD (AD SN) patients and NDC (NDC SN) with respect to GAPDH loading control (38 kDa) loading control. One lane represents one patient. Data information: Data shown as mean ± SEM. Each point represents the average per experimental replicate.

We and others have previously shown that microglia engulf synapses in mouse models of AD (Hong *et al*, 2016; Dejanovic *et al*, 2018; Gratuze *et al*, 2020). However, whether specific synapses are being eliminated by microglia has been unclear. A fundamental role of tissue‐resident macrophages is to recognize and selectively remove unwanted materials in their local microenvironment for proper maintenance of tissue homeostasis (Lemke, 2019). Hence, we hypothesized that healthy microglia, as brain‐resident macrophages, should eliminate dysfunctional synapses. To this end, we performed an *in vitro* synaptosome engulfment assay, where we measured microglial engulfment rate and level of pHrodo‐conjugated synaptosomes (Appendix Fig S1A and B). First, we isolated synaptosomes from the frontal cortex of AD patient brains or non‐demented control (NDC) human brains (please refer to Dataset EV1 for patient demographics). We used primary microglia from healthy C57BL6/J wild‐type (WT) mice which were supplemented with mCSF1, TGF‐β1, and CX3CL1 in order to induce a more homeostatic microglial transcriptome in culture (Butovsky *et al*, 2014; Gosselin *et al*, 2017; Fig EV1C). We observed a significantly more rapid and preferential phagocytosis of synaptosomes from AD patient brains versus those from NDC brains by microglia (Figs 1A and EV1D), suggesting selective recognition of certain molecules on AD patient synaptosomes. Low‐molecular‐weight Aβ oligomers have been associated with neuronal synaptic compartments in the AD brain (Li *et al*, 2009; Renner *et al*, 2010); accordingly, we found a significantly higher level of Aβ oligomers enriched in the synaptosomes of AD brains as compared to those of NDC brains (Fig EV1E). To directly test the role of Aβ oligomers in microglia‐synapse selectivity, we incubated WT mouse brain synaptosomes (Sodero *et al*, 2011) with soluble Aβ oligomers (Appendix Fig S1A and B) and simultaneously exposed both the Aβ oligomer‐bound and control synaptosomes to microglia for *in vitro* engulfment assay. Similar to human AD synaptosomes, microglia rapidly and preferentially engulfed the Aβ oligomer‐bound synaptosomes over control synaptosomes (Fig 1B–D). This discrimination by WT microglia lasted over 10 h of live‐cell time‐lapse imaging (Movie EV1). We confirmed that microglial preference for Aβ oligomer‐bound synaptosomes was independent of pHrodo dye conjugates used (Appendix Fig S2A–C) and that fluorescence signals were pH‐dependent (Appendix Fig S2D and E).

We next asked which molecular determinants on AD synaptosomes induce microglia phagocytosis. Interestingly, unbiased profiling of synaptic proteome in AD patients showed a significant enrichment of Annexin‐V (AnnxV; Hesse *et al*, 2019), a protein which binds ePtdSer, suggesting potentially higher levels of ePtdSer on AD synapses. We and others have previously shown that microglia engulf ePtdSer^+^ neuronal elements in the context of the developing brain (Li *et al*, 2020; Scott‐Hewitt *et al*, 2020). However, whether this occurs in Aβ‐relevant synapse engulfment, in the absence of neuronal apoptosis, is unknown. Indeed, several studies have shown in mouse models and human brains of AD that synapses present focal apoptotic‐like mechanisms before frank neuronal death including ePtdSer, a process coined synaptosis (Bader Lange *et al*, 2008; D'Amelio *et al*, 2011; Sokolova *et al*, 2021). In line, we observed that treating the freshly prepared mouse brain synaptosomes with Aβ oligomers results in increased externalization of PtdSer on outer synaptic membranes (Appendix Fig S1B), suggesting a direct relevance of Aβ oligomers on synaptic ePtdSer. Masking the ePtdSer signal on synaptosomes using AnnxV (Krahling *et al*, 1999) led to a marked decrease in microglial phagocytosis of both AD human and Aβ oligomer‐treated mouse synaptosomes (Figs 1A and E, and EV1D; Appendix Fig S1C and D). Altogether, these results suggest that microglia preferentially engulf Aβ^+^ synapses via ePtdSer recognition.

### Microglia phagocytose dendritic spines that become ePtdSer^+^ post‐Aβ challenge

**Figure EV2:**
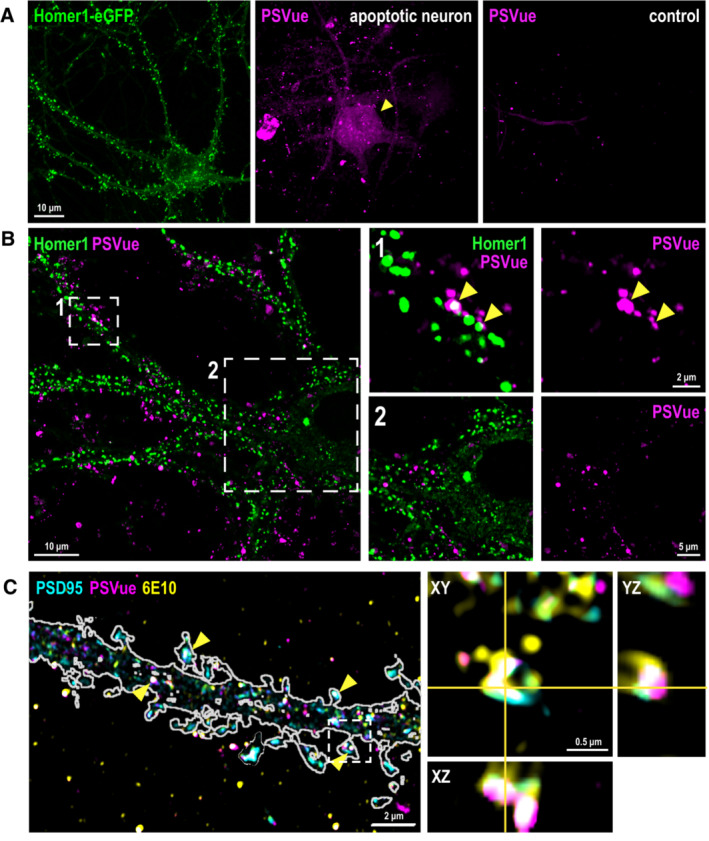
Aβ oligomers induce focal PtdSer externalization in dendritic spines ARepresentative image of Homer1‐eGFP neuron used in our studies here (green, left panel). In contrast to an apoptotic neuron (magenta, middle panel), where PSVue signals are observed in whole parts of the dying cell, the neurons used in our studies are nonapoptotic with negligent PSVue signal in the soma (right panel). Scale bar 10 μm.BSuper‐resolution Airyscan images of neuronal PSvue (magenta) staining after 1‐h treatment of 50 nM Aβ oligomer. Note PSVue signals in insets 1 and 2 are not random but are colocalized with or in close vicinity to Homer1‐eGFP signal as indicated by yellow arrowheads. Scale bar 10, 2, and 5 μm.CSuper‐resolution Airyscan images of PSD95 (cyan), PSVue (magenta) and 6E10 anti‐Aβ antibody (yellow) after 1‐h treatment with Aβ oligomer. Yellow arrowheads indicate the spines showing colocalized signal of PSD95, PSVue, and 6E10. Magnified orthogonal view of a single spine with PSD95, PSVue, and 6E10 colocalization in the inset. Scale bar 2 and 0.5 μm.

To confirm whether intact postsynaptic compartments focally externalize PtdSer for microglial targeting, we performed high‐resolution confocal Airyscan live‐cell imaging on Homer1‐eGFP hippocampal neurons (Ebihara *et al*, 2003). We followed in real time the Aβ oligomer‐induced ePtdSer signals using PSVue bis‐Zinc^2+^‐dipicolylamine (Zn‐DPA), which binds to negatively charged PtdSer when externalized on outer membranes (Hanshaw *et al*, 2005; Scott‐Hewitt *et al*, 2020). Application of soluble Aβ oligomers at physiologically relevant concentration (50 nM) rapidly and irreversibly elevated PSVue signals on Homer1‐eGFP^+^ postsynaptic puncta (Fig 1F and G). Super‐resolution microscopy (SRM) imaging confirmed punctate PSVue colocalization with Homer1‐eGFP^+^ puncta (Fig 1H) and along Aβ^+^ PSD95^+^ dendrites (Fig EV2C). Exposure to Aβ oligomers led to a markedly higher percentage of Homer1‐eGFP^+^ puncta displaying ePtdSer (Fig 1I). Furthermore, at the low Aβ concentrations and the timeframe of our imaging, the PSVue signals in the Aβ oligomer‐treated neurons were localized to Homer1‐eGFP^+^ signals and did not fluoresce across the whole neuron (Fig EV2A and B), as has previously been shown (Bader Lange *et al*, 2008; D'Amelio *et al*, 2011). In contrast, there were negligible levels of PSVue signal on Homer1‐eGFP^+^ puncta in control conditions throughout the timespan of imaging, suggesting healthy basal culture levels (Figs 1G and EV2A). In line, a higher proportion of PSVue signals were found to colocalize with Homer1‐eGFP^+^ spines in the Aβ oligomer‐treated versus control Homer1‐eGFP^+^ spines (Fig 1J). These results altogether suggest that synaptic ePtdSer induced by Aβ oligomers acts as an “eat‐me” signal on synapses for microglial engulfment.

**Figure 2:**
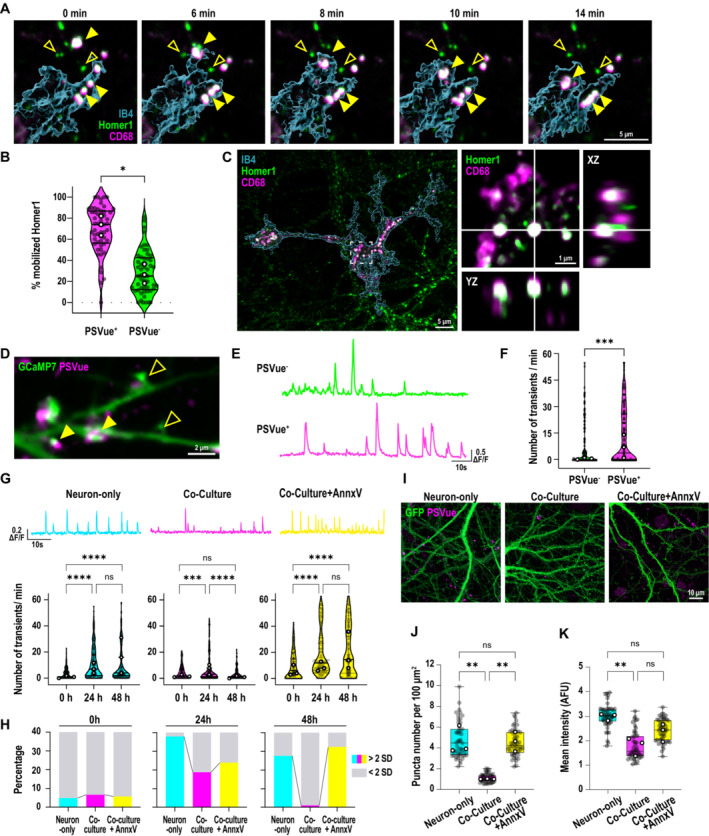
Microglia selectively engulf hyperactive ePtdSer^+^ spines and ameliorate Aβ oligomer‐induced neuronal hyperactivity A
Time‐lapse images of microglia (cyan, labeled with IB4‐647, 3D rendered) internalizing (yellow arrowheads) PSVue^+^ Homer1‐eGFP dendritic spines. Note PSVue^−^ Homer1‐eGFP dendritic spines are left behind (empty yellow arrowheads). Scale bar, 5 μm.B
Percentage of PSVue^+^ or PSVue^−^ Homer1 mobilized by microglial processes within 10 min of recording. Fifteen to thirty ROIs per experiment, three independent experiments.C
SRM images of Homer1‐eGFP and CD68 (magenta) lysosomes in microglia. Inset shows orthogonal view of colocalized Homer1‐eGFP and CD68. Scale bar, 5 μm.D
Representative image of transfected GCaMP7 signal on dendritic spines at 48‐h post‐Aβ oligomer challenge labeled with PSVue, closed and open arrows point to PSVue^+^ and PSVue^−^ spines, respectively. Scale bar, 2 μm.E
Representative trace of transfected postsynaptic GCaMP7 signals from PSVue^+^ and PSVue^−^ spines at 48‐h post‐treatment with Aβ.F
Number of spontaneous calcium transients per min in PSVue^+^ and PSVue^−^ spines. ∼10 spines per neuron, ∼5 neurons per experiment, from three independent experiments.G
(Top panel) Representative traces of spontaneous GCaMP7 signals at 48‐h post‐treatment with Aβ oligomer in dendritic spines from neuron‐only culture (cyan), neuron–microglia co‐culture (magenta), and neuron–microglia co‐culture treated with AnnxV (yellow). (Bottom panel) number of calcium transients per minute before (0 h), at 24‐h, and 48‐h post‐treatment of Aβ oligomer. ∼10 spines per neuron, ∼10 neurons per experiment, from three independent experiments.H
Percentage of spines showing high frequency (> mean + 2 SD; cyan, magenta, and yellow) compared with low frequency (< mean + 2 SD; gray) of calcium transients in each experimental group.I
SRM images of neuron‐only, neuron–microglia co‐culture, and neuron–microglia co‐culture treated with Annexin‐V (AnnxV) labeled with PSVue (magenta) 48 h after 50 nM Aβ oligomer treatment; neurons are transfected with GCaMP7‐eGFP (green). Scale bar, 10 μm.J, K
Number of PSVue puncta per 100 μm^2^ (J) and mean intensities of PSVue signal (K) in neuron‐only culture, and neuron–microglia co‐culture with or without AnnxV. Sixteen ROIs per experiment, *n* = 3 independent experiments. Data information: Data shown as mean (B), median (F and G) and frequency distribution (H) or box plots (J and K). The top and the bottom of the boxes represent the 75^th^ and 25^th^ percentiles, respectively, and the lines in the middle represent the median. The whiskers represent the highest and lowest values that are not outliers. Central bands of the violin plot represent median and quartiles. Each shaded point represents one ROI, and each open point represents the mean (or median for GCaMP studies) of each independent experiment. Paired *t*‐test (B) Mann–Whitney test (F), Kruskal–Wallis test followed by Dunn's multiple comparisons test (G) or one‐way ANOVA followed by Bonferroni *post hoc* test (J and K). *P*‐values shown ns *P* > 0.05; *P < 0.05; **P < 0.01; ***P < 0.001; ****P < 0.0001. Source data are available online for this figure.

**Figure EV3:**
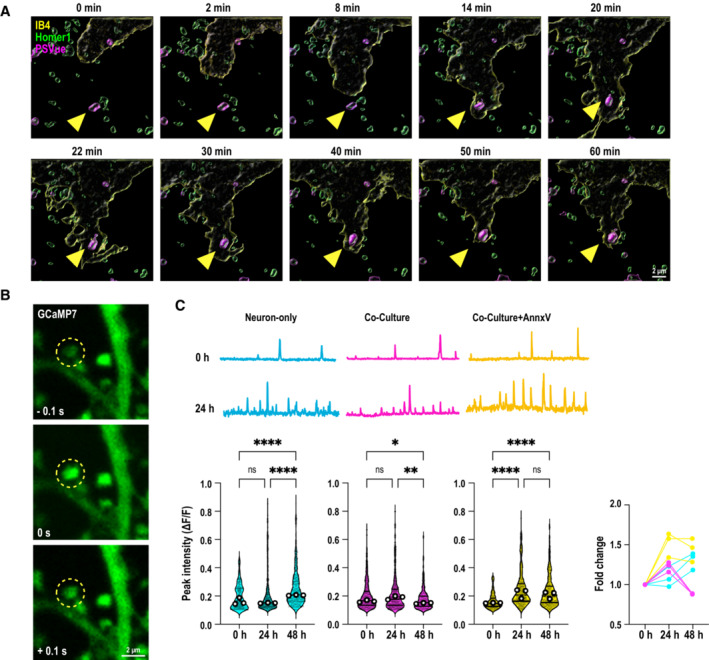
Primary microglia contact and remove ePtdSer^+^ dendritic spines to resolve neuronal hyperactivity upon acute Aβ oligomer challenge ATime‐lapse sequence images of 10 consecutive optical sections (Δz = 0.4 μm) showing primary Homer1‐eGFP neurons (green), PSVue (magenta) and microglia (yellow, labeled with Isolectin B4‐647, IB4‐647). Yellow arrowheads indicate PSVue^+^ Homer1‐eGFP dendritic spines contacted by microglia over 1 h. See Movie EV2. Scale bar 2 μm.BRepresentative image of transfected GCaMP7 signal on dendritic spines at 48‐h post‐oAβ challenge. Scale bar 2 μm.C(Top panel) Representative traces of GCaMP7 signal in dendritic spines at 0‐h and 24‐h post‐treatment with Aβ oligomer from neuron‐only culture (cyan), neuron–microglia co‐culture (magenta), and neuron–microglia co‐culture treated with Annexin‐V (AnnxV, yellow). (Bottom panel) Violin plots showing normalized peak intensities (Δ*F*/*F*_0_) of spontaneous GCaMP7 signal in dendritic spines before (0 h), at 24‐h and 48‐h post‐treatment with Aβ oligomer. (Bottom right) Relative fold change. ∼10 spines per neuron, ∼10 neurons per experiment, from three independent experiments. Data information: Data shown as violin plot, central bands represent median and quartiles. Each shaded point represents one ROI, and each open point represents the median of each independent experiment. Kruskal–Wallis test followed by Dunn's multiple comparisons test. *P*‐values shown as ns *P* > 0.05; *P < 0.05; **P < 0.01; ****P < 0.0001.

We next tested whether synaptic ePtdSer initiates targeted microglial phagocytosis, in the absence of cellular death. Microglia have been suggested to phagocytose viable neurons and synapses in Aβ context (Neniskyte *et al*, 2011); however, whether this process occurs on the level of apoptotic synapses (vs. apoptotic cells; Damisah *et al*, 2020) is unclear. In fact, direct phagocytosis of synapses by microglia has been debated (Weinhard *et al*, 2018). Distinguishing this is important, because it could suggest a role for microglia in attempting to fine‐tune synaptic homeostasis in earliest stages of AD when synapses are vulnerable, as microglia have been shown to do so in healthy adult brains (Badimon *et al*, 2020; Cserép *et al*, 2020). To that end, we assessed microglia‐synapse interactions with labeled Homer1‐eGFP spines in real time. Using live‐cell imaging with 3D high‐resolution capacity and AiryScan, we found that microglial processes first contact the Homer1‐eGFP^+^ spines that become PSVue^+^ upon Aβ oligomer challenge (Fig EV3A; Movie EV2) and then engulf (Fig 2A; Movie EV3). Interestingly, targeting and contacting of PSVue^+^ eGFP^+^ spines by microglial processes occur for some time (Fig EV3A), whereas engulfment itself appears to be a rapid event (Fig 2A). Importantly, a significantly higher percentage of eGFP^+^ synapses which were internalized by microglia post‐contact were PSVue^+^ (73 vs. 27% PSVue‐; Fig 2B; Movie EV2), suggesting that intact spines that become ePtdSer^+^ are preferentially targeted and engulfed by microglia. SRM imaging further showed lysosomal localization of the engulfed Homer1‐eGFP^+^ (Fig 2C). These data altogether suggest that microglia phagocytose intact ePtdSer^+^ synapses in an Aβ oligomer‐relevant context.

### Microglia remove hyperactive ePtdSer^+^ spines for the resolution of neuronal activity

Synaptic calcium activity is known to be increased upon Aβ oligomer treatment (Arbel‐Ornath *et al*, 2017; Zott *et al*, 2019). Interestingly, spontaneous calcium signals in spines were significantly higher in PSVue^+^ spines than PSVue^−^ ones (Fig 2D–F; Movies EV4 and EV5). We further observed that PtdSer externalization is dependent on calcium influx induced by neuronal depolarization (Appendix Fig S3), suggesting that the effect of oligomeric Aβ on synaptic PtdSer externalization involves changes in calcium signaling. To address the role in microglia‐ePtdSer interaction in neuronal hyperactivity, we measured spontaneous calcium transients in dendritic spines post‐Aβ oligomer challenge in neuron‐only cultures versus microglia–neuron co‐cultures. In neuron‐only cultures, neuronal hyperactivity was induced by Aβ oligomers within 24 h and stayed elevated; however, in microglia–neuron co‐cultures, Aβ oligomer‐induced neuronal hyperactivity was ameliorated back to baseline levels within 48 h (Figs 2G and EV3B and C), suggesting that microglia work to resolve neuronal hyperactivity. Interestingly, in‐depth analysis further showed that the fraction of spines displaying high‐frequency calcium transients (with frequency levels > mean + 2 SD of the initial value) were almost absent in microglia–neuron co‐culture despite Aβ oligomer challenge, as compared to neuron‐only cultures (Fig 2H). In line, the levels of synaptic PSVue were significantly less in Aβ‐treated microglia–neuron co‐culture versus those in neuron‐only culture at 48 h (Fig 2I–K). These data altogether suggest that microglia may selectively target hyperactive ePtdSer^+^ spines.

Thus, we next asked whether the resolution of neuronal hyperactivity by microglia requires ePtdSer^+^ recognition. AnnxV masking of ePtdSer on synaptosomes prevents microglial engulfment of PSVue^+^ synapses (Figs 1E and EV1D; Appendix Fig S1C and D). In accord, AnnxV treatment resulted in sustained elevated levels of synaptic PSVue in the microglia–neuron co‐cultures, at levels similar to the neuron‐only cultures (Fig 2I–K), suggesting that microglia are no longer able to clear ePtdSer^+^ spines. Importantly, in these AnnxV‐treated microglia–neuron co‐cultures, neuronal hyperactivity was no longer ameliorated, and we observed sustained elevation of dendritic calcium levels at 48 h (Fig 2G). In line, the percentage of high‐frequency spines continued to increase (Fig 2H). These results suggest that microglia work to resolve Aβ oligomer‐induced neuronal hyperactivity via the selective removal of ePtdSer^+^ hyperactive synapses.

### TREM2 is required for microglial removal of synapses in Aβ‐contexts

**Figure 3:**
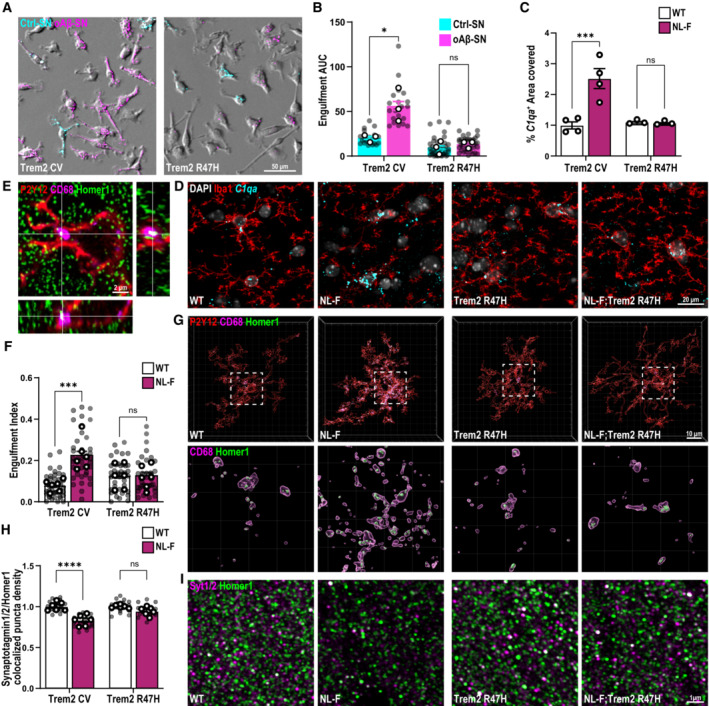
Microglia require TREM2 to engulf synapses in *in vitro* and *in vivo* Aβ models A
Trem2 CV and Trem2 R47H KI primary microglia treated simultaneously with oAβ‐bound synaptosomes (SN) conjugated with pHrodo red (magenta) and control SN, conjugated with pHrodo deep red (cyan). Scale bar, 50 μm.B
pHrodo fluorescence with time shown as AUC at 3 h. AUC of oAβ‐SN is higher compared with Ctrl‐SN in Trem2 CV but not in Trem2 R47H KI microglia. ∼40 microglia per ROI, two ROIs per well, 2–3 wells per experiment, *n* = 3 independent experiments.C
Quantification of the percent of *C1qa* area covered normalized to WT showing an increase in the NL‐F but not NL‐F KI; Trem2 R47H KI. *N* = 3–4 animals per genotype.D
Representative images from hippocampal CA1 from 6‐month‐old WT, Trem2 R47H KI, NL‐F KI, and NL‐F KI; Trem2 R47H KI mice probing for *C1qa* (cyan) by *in situ* hybridization (RNAScope) followed by Iba1 (red) immunostaining. Scale bar, 20 μm.E–G
Hippocampal CA1 SR of 6 mo WT, Trem2 R47H KI, NL‐F KI, and NL‐F KI; Trem2 R47H KI mice immunostained for P2Y12 (red), CD68 (magenta), and Homer1 (green). (E) Orthogonal image showing engulfed Homer1 (green) inside CD68 (magenta)‐immunoreactive lysosomes in P2Y12^+^ (red) microglia. Scale bar, 2 μm. (F) Quantification of engulfment index (volume of Homer1 in CD68/microglial cell volume)*100). Six to nine microglia per animal, *n* = 6 animals per genotype. (G) Representative 3D surface rendering reconstructions showing increased Homer1 in microglia in NL‐F KI compared with WT, Trem2 R47H KI, and NL‐F KI; Trem2 R47H KI mice. Scale bar, 10 μm.H
Colocalized puncta density normalized to WT or Trem2 R47H KI accordingly showing decreased synapse density in NL‐F KI but not NL‐F KI; Trem2 R47H KI. Three ROIs per animal, *n* = 6 animals per genotype.I
SRM images from the hippocampal CA1 SR of 6 mo WT, Trem2 R47H KI, NL‐F KI and NL‐F KI; Trem2 R47H KI mice immunostained for Synaptotagmin (Syt1/2, magenta) and Homer1 (green), pre‐ and postsynaptic puncta, respectively. Scale bar, 1 μm. Data information: Data shown as mean ± SEM. Each shaded point represents one ROI, and each open point represents the mean of each independent experiment. Two‐way ANOVA followed by Bonferroni's *post hoc* test. *P*‐values shown as ns *P* > 0.05; *P < 0.05; ***P < 0.001; ****P < 0.0001. Source data are available online for this figure.

**Figure EV4:**
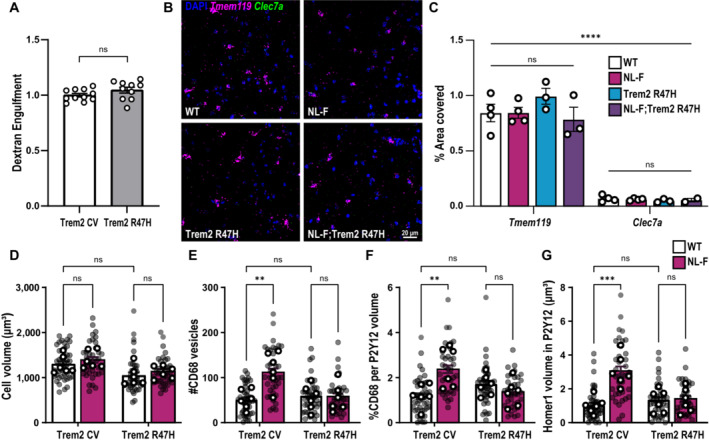
Microglia require functional TREM2 to engulf synapses *in vivo* A
Primary microglia (P0‐P4) prepared from either Trem2 CV or Trem2 R47H KI mice treated with dextran conjugated to pHrodo red to compare engulfment of inert particles, showing no difference between the two genotypes.B
Representative images from hippocampal CA1 stratum lacunosum‐moleculare from 6‐month‐old WT, Trem2 R47H KI, NL‐F KI, and NL‐F KI; Trem2 R47H KI mice probing for *Tmem119* (magenta) and *Clec7a* (green) by *in situ* hybridization (RNAScope). DAPI shown in blue. Scale bar, 20 μm.C
Quantification of the percent of area covered either by *Tmem119* or *Clec7a* spots showing higher levels of *Tmem119* compared with *Clec7a* across all genotypes with no difference between genotypes. *n* = 2–4 animals.D–G
Hippocampal CA1 stratum radiatum sections from 6‐month‐old WT, Trem2 R47H KI, NL‐F KI, and NL‐F KI; Trem2 R47H KI mice immunostained for P2Y12 (red), CD68 (magenta), and Homer1 (green). 3D surface rendering reconstructions of microglia showing increased Homer1 signal inside microglia in NL‐F KI but not NL‐F KI; Trem2 R47H KI compared with WT and Trem2 R47H KI, respectively. Quantification of microglial P2Y12^+^ cell volume (D), number of CD68^+^ lysosomal vesicles per microglia (E), percentage of P2Y12^+^ volume occupied by CD68^+^ immunoreactive vesicles (F), and total volume of Homer1^+^ material within P2Y12^+^ microglia (G). All volumes are represented in μm^3^. Six to nine microglia per animal, *n* = 6 animals per genotype. Data information: Data shown as mean ± SEM. Each shaded point represents one ROI, and each open point represents the average per experimental replicate. Unpaired *t*‐test (A) or two‐way ANOVA followed by Bonferroni's *post hoc* test. *P*‐values shown as ns *P* > 0.05; **P < 0.01; ***P < 0.001; ****P < 0.0001.

We then tested whether TREM2, which has been linked to ePtdSer recognition (Wang *et al*, 2015), is involved in Aβ^+^ synaptic engulfment by microglia. In line, TREM2 has also been shown to modulate microglia‐synapse engulfment in the developing brain (Filipello *et al*, 2018; Scott‐Hewitt *et al*, 2020) as well as in models of tau‐mediated neurodegeneration (Gratuze *et al*, 2020). Indeed, we found that microglia from Trem2 R47H knock‐in (KI) mice, where TREM2 expression (Xiang *et al*, 2018) and function (Kleinberger *et al*, 2014) are impaired, no longer displayed preferential engulfment of Aβ^+^ synapses over control ones (Fig 3A and B). Interestingly, there was no altered phagocytic efficiency for inert particles in the KI microglia versus WT microglia (Fig EV4A; Filipello *et al*, 2018), suggesting that it is not a general failure of KI microglia to phagocytose. Furthermore, there were no gross differences in microglial motility or morphology between WT and KI microglia (Movie EV6).

To address whether the failure of Trem2 KI microglia to detect ePtdSer^+^ synaptosomes *in vitro* is recapitulated *in vivo*, we crossed the Trem2 R47H KI mice with the hAPP NL‐F KI mice. The NL‐F KI allows for endogenous control of Aβ production (Saito *et al*, 2014); furthermore, amyloidosis is slow‐progressing, which allows for a higher temporal resolution for microglial analysis at distinct stages of the disease. This is important to distinguish because in plaque‐enriched stages, there are significant plaque‐induced microglial cell states, some of which are TREM2‐dependent (Keren‐Shaul *et al*, 2017; Wood *et al*, 2022). Thus, here we focused on preplaque stages when Aβ oligomers start depositing and synapses are already found vulnerable (Colom‐Cadena *et al*, 2020). At this age in the NL‐F hippocampus, we do not yet see a significant level of plaques or disease‐associated microglia (DAM; Keren‐Shaul *et al*, 2017; De Schepper *et al*, 2023; Fig EV4B and C). However, synapses are already vulnerable to C1q‐dependent microglial engulfment at these preplaque stages (Hong *et al*, 2016; De Schepper *et al*, 2023). The increase of microglial *C1qa* levels in the NL‐F hippocampus was ameliorated in the NL‐F; Trem2 hippocampus (Fig 3C and D). We next assessed for levels of synaptic engulfment in microglia. As expected, we found a significantly higher volume of Homer1‐immunoreactive synaptic puncta inside CD68^+^ lysosomes of P2Y12^+^ microglia in the 6 mo NL‐F hippocampus, as compared to those in age‐ and sex‐matched WT controls (Figs 3E–G and EV4D–G), suggesting that microglia engulfed synapses in the NL‐F hippocampus. However, there were no appreciable differences between levels of engulfed Homer1‐immunoreactive synaptic puncta between 6 mo NL‐F; Trem2 and Trem2 KI mice, suggesting that proper TREM2 function is required for microglia to engulf synapses in Aβ mouse models. Levels were also comparable between hippocampus of WT and Trem2 KI mice, suggesting that Trem2 KI mice do not display overt defects in engulfment at baseline (Figs 3F and G, and EV4D–G). In line with the microglia‐synapse engulfment and microglial *C1qa* levels, SRM imaging showed loss of excitatory synapses, as assessed by colocalization of Homer1‐ and Synaptotagmin 1/2‐immunoreactive synaptic puncta, in the NL‐F hippocampus, but not in the NL‐F;Trem2 hippocampus (Fig 3H and I). These results altogether suggest that proper TREM2 function is required for microglia to remove synapses *in vivo* and *in vitro*.

### TREM2 loss‐of‐function impairs microglial ability to restore neuronal activity and leads to the accumulation of apoptotic‐like synapses

**Figure 4:**
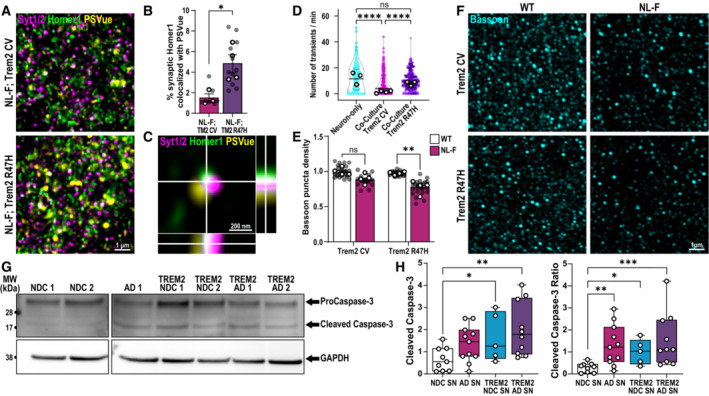
TREM2 loss‐of‐function leads to increased levels of apoptotic‐like synapses in mouse and human brains AHippocampal dentate gyrus hilus of 6 mo NL‐F KI and NL‐F KI; Trem2 R47H KI mice ICV injected with PSVue 643 (yellow) and immunostained for Synaptotagmin 1/2 (magenta) and Homer1 (green). Scale bar, 1 μm.BPercentage of synaptic Homer1‐immunoreactive puncta found colocalized with PSVue in NL‐F KI; Trem2 R47H compared with NL‐F KI. Three ROIs per animal, *n* = 3–4 animals per genotype.COrthogonal SRM image showing colocalized PSVue (yellow) with synaptic markers, Syt1/2 (magenta) and Homer1 (green). Scale bar 200 nm.DNumber of spontaneous calcium transients per minute at 48‐h post‐treatment of Aβ oligomer in neuron‐only culture (cyan), neuron‐Trem2 CV microglia co‐culture (magenta) or neuron‐ Trem2 R47H KI microglia co‐culture (purple). Approximately ten spines per neuron, ∼10 neurons per experiment, from 3 to 4 independent experiments.EQuantification of Bassoon‐immunoreactive puncta density in four different genotypes. Six ROIs per animal, *n* = 4 animals per genotype.FSRM 3D images from the hippocampal CA1 SR of 6 mo WT, Trem2 R47H KI, NL‐F KI, and NL‐F KI; Trem2 R47H KI mice immunostained for Bassoon (cyan), a presynaptic marker enriched in functional active zones. Scale bar, 1 μm.GRepresentative western blots comparing levels of cleaved caspase‐3 (17/19 kDa) and procaspase‐3 (35 kDa) with respect to GAPDH (38 kDa) loading control in synaptosomes isolated from human NDC, AD, NDC TREM2 and AD TREM2 brains. One lane represents one patient.HWestern blot densitometry analysis showing cleaved caspase‐3 levels (left graph) and the ratio of cleaved caspase‐3/procaspase‐3 (right graph) in synaptosomes (SN) isolated from human NDC, AD, TREM2 NDC, and TREM2 variants. *N* = 9 NDC, 11 AD, 5 TREM2 NDC, 10 TREM2 AD cases. Data information: Data shown as mean ± SEM. Each shaded point represents one ROI, and each open point represents the mean (or median for GCaMP studies) of each independent experiment. Central bands of the violin plot (D) represent median and quartiles. The top and the bottom of the box plot (H) represent the 75^th^ and 25^th^ percentiles, respectively, and the line represents the median. The whiskers represent the highest and lowest values that are not outliers. Unpaired *t*‐test (B), Kruskal–Wallis test followed by Dunn's test (D), two‐way (E), or one‐way (H) ANOVA followed by Bonferroni's *post hoc* test. *P*‐values shown as ns *P* > 0.05; *P < 0.05; **P < 0.01, ****P < 0.0001. Source data are available online for this figure.

**Figure EV5:**
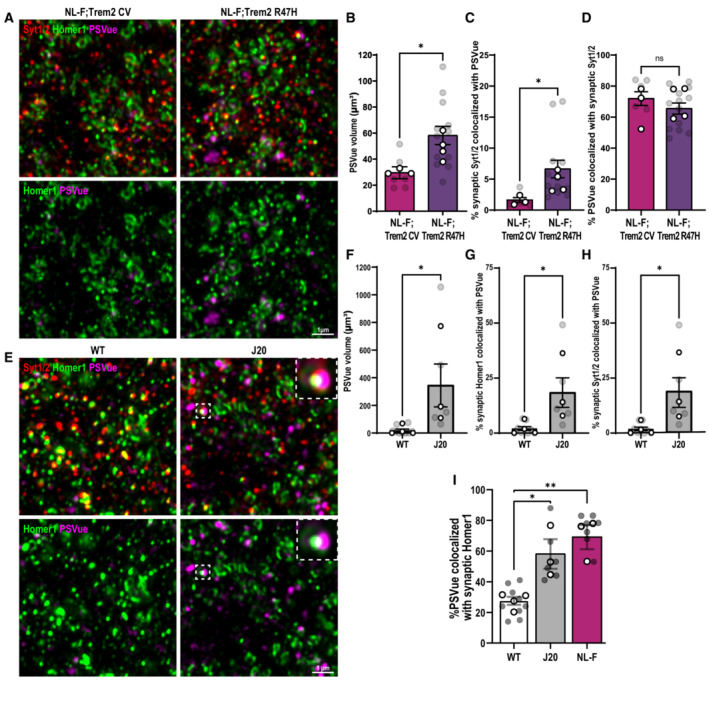
Trem2 loss‐of‐function exacerbates synaptic ePtdSer in the NL‐F model of amyloidosis ASuper‐resolution images from the hippocampal CA1 stratum radiatum of 6‐month‐old NL‐F KI and NL‐F KI; Trem2 R47H KI mice immunostained for presynaptic Synaptotagmin 1/2 (Syt1/2, red) and postsynaptic Homer1 (green). PSVue 643 (magenta) is ICV injected. Upper panels show Syt1/2, Homer1, and PSVue. Lower panels show Homer1 and PSVue only. Scale bar 1 μm.BPSVue volume represented in μm^3^. Three ROIs per animal, *n* = 3–4 per genotype.CPercentage of synaptic Synaptotagmin 1/2 puncta within 0.25 μm of PSVue showing increased percentage of PSVue^+^ synapses in NL‐F KI; Trem2 R47H KI mice compared with NL‐F KI. Three ROIs per animal, *n* = 3–4 animals per genotype.DPercentage of PSVue volume within 0.25 μm of synaptic Synaptotagmin 1/2 puncta. Three ROIs per animal, *n* = 3–4 animals per genotype.ESuper‐resolution images from the hippocampal CA1 dentate gyrus hilus of 4‐month‐old WT and J20 Tg mice immunostained for pre‐Synaptotagmin 1/2 (Syt1/2, red) and postsynaptic Homer1 (green). PSVue 643 (magenta) is ICV injected. Top panels show Syt1/2, Homer1, and PSVue. Bottom panels show Homer1 and PSVue only. Insets show either triple (Syt1/2, Homer1 and PSVue; top panel) or double colocalization (Homer1 and PSVue; bottom panel). Scale bar 1 μm.FPSVue total volume per ROI (7,500 μm^3^) showing increased PSVue in J20 Tg mice compared with WT. Three ROIs per animal, *n* = 3–4 animals per genotype.GPercentage of synaptic Homer1 puncta within 0.25 μm of PSVue showing an increase in PSVue^+^ synapses in J20 Tg compared with WT. Three ROIs per animal, *n* = 3–4 animals per genotype.HPercentage of synaptic Synaptotagmin 1/2 puncta within 0.25 μm of PSVue showing an increase in PSVue^+^ synapses in J20 Tg compared with WT. Three ROIs per animal, *n* = 3–4 animals per genotype.IPercentage of PSVue volume within 0.25 μm of synaptic Homer1 puncta. Three ROIs per animal, *n* = 3–4 animals per genotype. Data information: Data shown as mean ± SEM. Each shaded point represents one ROI, and each open point represents the average per experimental replicate. Two‐way ANOVA followed by Bonferroni's *post hoc* test. *P*‐values shown as ns *P* > 0.05; *P < 0.05; **P < 0.01.

Given that we found a role for microglia in removing ePtdSer^+^ synapses (Figs 1 and 2) in a Trem2‐dependent manner (Fig 3), we next asked whether there is a higher level of ePtdSer^+^ synapses remaining in the NL‐F;Trem2 hippocampus. To this end, we performed intracerebroventricular (ICV) injection of PSVue (Scott‐Hewitt *et al*, 2020) and SRM imaging to visualize synaptic ePtdSer, that is, PSVue colocalized on Homer1‐ and synaptotagmin 1/2‐immunoreactivate puncta. Compared with healthy young adult WT mice, we found higher percentages of synapses that were PSVue^+^ in preplaque hippocampus of two mouse models of Aβ pathology, that is, 6 mo NL‐F KI and 3 mo J20 transgenic mice (Fig EV5). Interestingly, the levels of synaptic ePtdSer were even more significantly elevated in hippocampus of NL‐F;Trem2 as compared to those of NL‐F (Figs 4A–C and EV5), suggesting that a proportion of synapses in the NL‐F;Trem2 hippocampus reflect ePtdSer^+^ synapses which are not being properly cleared by the Trem2 KI microglia. To better understand the impact of Trem2 loss‐of‐function in Aβ‐induced neuronal hyperactivity, we tested whether primary microglia prepared from Trem2 KI mice were able to restore neuronal activity at 48 h upon Aβ oligomer challenge in our neuron–microglia GCaMP7 paradigm (Fig 2G). We found that Trem2 KI microglia failed to resolve Aβ oligomer‐induced neuronal hyperactivity (Fig 4D) similar to what we observed in WT microglia treated with AnnxV (Fig 2G). These data suggest that functional TREM2 is required for the removal of ePtdSer^+^ synapses and to resolve neuronal hyperactivity. In line, we found that the levels of Bassoon, which plays an important role for function of the active zone (Altrock *et al*, 2003; Lazarevic *et al*, 2011), were found to be the most decreased in the NL‐F;Trem2 hippocampus (Fig 4E and F), suggesting a potential exacerbated synaptic dysfunction in the 6 mo NL‐F; Trem2 brain, as opposed to the NL‐F.

Finally, we assessed the translational relevance of ePtdSer^+^ synapses in patient brains. Direct PSVue labeling is not possible in frozen postmortem samples. However, PtdSer is canonically externalized by the cleavage of caspase 3 (Ertürk *et al*, 2014; Segawa *et al*, 2014) as such, we probed for levels of full‐length and cleaved caspase‐3 by western blotting on isolated human synaptosomes. We found increased levels of caspase‐3 ratio in synaptosomes isolated from human AD brains as compared to those from NDC brains (Fig 4G and H; *N* = 9 for NDC, *N* = 11 for AD, *N* = 5 TREM2 NDC and *N* = 10 TREM2 AD). Due to limited availability of TREM2 patient tissue, we were only able to sample a small cohort of patients carrying AD‐risk‐associated TREM2 variants. In this cohort, we found significantly higher levels of caspase‐3 activation in TREM2 AD synaptosomes compared with NDC and similarly when comparing synaptosomes derived from patients without pathology, between TREM2 NDC and NDC (Fig 4G and H). These data may suggest that TREM2 loss‐of‐function may impact human brains likewise to what we observed in mouse models, but larger cohort analysis is needed to better elucidate the relationship between TREM2 function and apoptotic‐like synapses in humans.

## Discussion

In AD, many of the risk factors identified via genome‐wide association studies converge on phagocytic pathways of microglia (Podleśny‐Drabiniok *et al*, 2020). This raises a critical need to understand how microglial phagocytosis in the aging and diseased brain fail and result in prolonged dyshomeostasis and increased susceptibility to neurodegeneration. Here, we show that microglia selectively target specific synapses for phagocytosis, which aids in the resolution of neuronal homeostasis. In particular, we demonstrate that Aβ oligomers induce synapse dysfunction, as measured by spontaneous calcium activity and synaptic exposure of PtdSer. Microglia then target and remove these ePtdSer^+^ synapses, resulting in amelioration of neuronal hyperactivity. Furthermore, we show that TREM2, a major risk factor for late‐onset AD (Guerreiro *et al*, 2013; Jonsson *et al*, 2013), mediates the selective engulfment of Aβ‐damaged synapses. In accord, there are increased levels of apoptotic‐like synapses remaining in the brains of AD humans and mouse models with dysfunctional TREM2. Our studies altogether suggest a role for ePtdSer‐TREM2 in microglial engulfment of synapses in Aβ^+^ models. Furthermore, we suggest that the microglial engulfment of synapses, at least in Aβ oligomer‐relevant context which likely reflects the earliest stages of disease, is beneficial.

Our data further suggest that ePtdSer^+^ synapses represent a dysfunctional apoptotic‐like population whereby microglia‐mediated removal is necessary to maintain neural homeostasis. Whereas microglia can engulf whole apoptotic neuronal cells in various injury and disease contexts (Tufail *et al*, 2017; Brelstaff *et al*, 2018; Garcia‐Reitboeck *et al*, 2018), in the normal healthy brain, ePtdSer signals have been shown to occur on synaptic and other subcellular compartments of neurons to promote their phagocytosis in a context‐dependent manner (Ruggiero *et al*, 2012; Li *et al*, 2020; Scott‐Hewitt *et al*, 2020). In mouse models, mild cognitive impairment (MCI) and AD patients, local increase of apoptotic‐like signaling including ePtdSer occurs on synapses (our data here, Mattson *et al*, 1998; Bader Lange *et al*, 2008; D'Amelio *et al*, 2011; Park *et al*, 2020; Phongpreecha *et al*, 2021). Here, we propose a model whereby Aβ oligomers, which are predominantly generated as a natural by‐product of activity‐dependent APP cleavage (Cirrito *et al*, 2008), induce aberrant hyperactivity and externalization of PtdSer upon interaction with synapses. Dysfunction and ePtdSer could be induced in multiple ways: Inefficient clearing of Aβ from synapses can lead to Aβ deposition and aggregation on membranes and/or disruption of membrane dynamics including glutamatergic transmission and synaptic plasticity (Li *et al*, 2009; Renner *et al*, 2010; Zott *et al*, 2019), altogether resulting in mitochondrial dysfunction and local activation of caspase‐3, a well‐established modulator of flippases and scramblases that regulate ePtdSer (Segawa *et al*, 2014; Lemke, 2019; Sokolova *et al*, 2021). ePtdSer then acts as an “eat‐me” signal, rendering these synapses susceptible to microglial engulfment.

One mechanism by which ePtdSer is recognized by macrophages is via TREM2 (Wang *et al*, 2015). Whereas the role of TREM2 in plaque‐ and tangle‐related neuropathology in AD has been widely studied (Deczkowska *et al*, 2020; Gratuze *et al*, 2020; Lee *et al*, 2021), little is known whether TREM2 impacts synapse engulfment in preplaque brains. Here, we show that microglia with dysfunctional TREM2 fail to engulf AD ePtdSer^+^ synapses, which results in an increased burden of uncleared, apoptotic‐like synapses in the hippocampus. These data, along with significantly decreased levels of Bassoon, suggest an exacerbation of presynaptic defects in these preplaque brains. Altogether, this suggests that TREM2 is important for clearing apoptotic‐like synapses in the aging and diseased brains and provides a possible explanation of how TREM2 loss‐of‐function variants may lead to an increased risk of AD.

Our data further suggest that TREM2 may also act upstream of C1q‐mediated microglia‐synapse engulfment in amyloid models, in line with a previous study in a tau model of AD (Gratuze *et al*, 2020). We and others have previously shown that microglia eliminate synapses in a complement‐dependent manner in Aβ‐ and tau‐based models (Hong *et al*, 2016; Dejanovic *et al*, 2018); however, upstream mediators determining which synapses to target were not known. Interestingly, C1q has been highlighted as putative binding partner of ePtdSer (Païdassi *et al*, 2008) and proteomics analysis suggests that C1q‐tagged synaptosomes are enriched in apoptotic‐like features (Györffy *et al*, 2018). Altogether these studies suggest that TREM2‐ePtdSer‐C1q may converge on the same pathway to mediate microglia‐synapse elimination. The exact mechanisms of how TREM2 modulates synapse phagocytosis as well as the functional consequences of ePtdSer‐TREM2 microglia‐synapse engulfment in the plaque‐enriched, aged brains are to be determined.

Finally, we show that there is selectivity to which synapses microglia engulf in AD‐relevant context. Our data suggest that in preplaque AD brains, externalization of PtdSer at synapses, potentially as a consequence of Aβ‐induced excessive synaptic activity, is used as a focal damage signal which is recognized by microglia to help to maintain neural homeostasis. Given that microglia are equipped with a multitude of phagocytic receptors and sensors that have been shown to directly or indirectly interact with ePtdSer (Lemke, 2019), it is likely that TREM2 is not the sole mechanism by which ePtdSer is recognized and removed in AD brains (Tufail *et al*, 2017; Brelstaff *et al*, 2018). An ensemble of “eat‐me” (Hong *et al*, 2016) and “don't‐eat‐me” (Lehrman *et al*, 2018) molecules on synapses may together determine the precision with which synapses are removed or not removed. Given the importance of synaptic homeostasis, especially as cells become senescent in the aging brain, what decisive and rapid alliance of various signaling molecules regulate microglia‐synapse engulfment will be important to determine.

## Materials and Methods

### Reagents and Tools table

**Table:** 

qPCR primers	
Gene	Primer sequence 5′‐3′
*Actb*	Forward: CATTGCTGACAGGATGCAGAAGG, Reverse: TGCTGGAAGGTGGACAGTGAGG
*Cx3cr1*	Forward: GAGTATGACGATTCTGCTGAGG, Reverse: CAGACCGAACGTGAAGACGAG
*Gapdh*	Forward: CATCACTGCCACCCAGAAGACTG, Reverse: ATGCCAGTGAGCTTCCCGTTCAG
*Gfap*	Forward: CACCTACAGGAAATTGCTGGAGG, Reverse: CCACGATGTTCCTCTTGAGGTG
*Itgam*	Forward: ATGGACGCTGATGGCAATACC, Reverse: TCCCCATTCACGTCTCCCA
*Mag*	Forward: GGCCGAGGAGCAAGAATGG, Reverse: CATGCACTCTGCGATACGCT
*Map2*	Forward: ATGACAGGCAAGTCGGTGAAG, Reverse: CATCTCGGCCCTTTGGACTG
*Rpl32*	Forward: ATCAGGCACCAGTCAGACCGAT, Reverse: GTTGCTCCCATAACCGATGTTGG
*Tmem119*	Forward: CCTACTCTGTGTCACTCCCG, Reverse: CACGTACTGCCGGAAGAAATC
*Trem2*	Forward: CTGGAACCGTCACCATCACTC, Reverse: CGAAACTCGATGACTCCTCGG

Primary antibodies				
Antibody target	Catalog no.	Company	Host	Dilution
Homer1	160006	Synaptic Systems	Chicken	1/200
Synaptotagmin 1/2	105002	Synaptic Systems	Rabbit	1/200
Bassoon	141003	Synaptic Systems	Rabbit	1/200
6E10	803001	BioLegend	Mouse	1/1,000
4G8	800708	BioLegend	Mouse	1/1,000
GAPDH	ab181602	Abcam	Rabbit	1/20,000
Synaptophysin	ab8049	Abcam	Mouse	1/1,000
PDS‐95	MAB1596	Merck	Mouse	1/1,000
PSD‐95	124014	Synaptic Systems	Guinea pig	1/1,000
Cleaved caspase‐3	9661S	Cell Signalling	Rabbit	1/1,000
Caspase‐3	9662	Cell Signalling	Rabbit	1/1,000
Iba1	019‐19741	Wako Chemicals	Rabbit	1/500
P2Y12	AS‐55043A	Anaspec	Rabbit	1/500
CD68	MCA‐1957	Serotec	Rat	1/500

Secondary antibodies				
Fluorophore tag	Catalog no.	Company	Host	Dilution
Anti‐Chicken 488	A11039	ThermoFisher	Goat	1/500
Anti‐Rabbit 594	A11037	ThermoFisher	Goat	1/500
Anti‐Rabbit 546	A11035	ThermoFisher	Goat	1/500
Anti‐Rabbit 647	A27040	ThermoFisher	Goat	1/1,000
Anti‐Rat 647	A21247	ThermoFisher	Goat	1/500
Anti‐ Mouse HRP	ab205719	Abcam	Goat	1/5,000
Anti‐ Rabbit HRP	ab205718	Abcam	Goat	1/10,000
Anti‐Mouse 800	A32789	ThermoFisher	Goat	1/1,000
Anti‐Rabbit 680	A32734	ThermoFisher	Goat	1/1,000

### Methods and Protocols

#### Animals

All experiments have been reviewed by UCL's animal care committees and conducted in accordance with the regulations set out in the Animals in Scientific Procedures Act (ASPA) 1986. Sprague Dawley rats obtained from Charles River UK and Homer1‐eGFP (Ebihara *et al*, 2003) obtained from Japan (kind gift from S. Okabe) were used for primary neuronal cultures at embryonic day (E18). C57BL6/J (WT) mice obtained from Charles River UK and Trem2 R47H KI mice (imported from JAX, C57BL/6J‐*Trem2* ^*em1Adiuj/J*^ Strain #027918) were used for primary microglial culture preparation at P0‐P4. App^NL‐F^ KI mice (Saito *et al*, 2014; kindly provided by Takaomi Saido, Riken and distributed by Frances Edwards, UCL) were crossed to Trem2 R47H KI mice for *in vivo* labeling of lipids, synapse loss, and microglial engulfment studies. APP transgenic J20 mice (kindly provided by Lennart Mucke and distributed by Patricia Salinas, UCL) were used at age 3 months for *in vivo* labeling of lipids. For all experiments, appropriate sex‐ and age‐matched controls were used. For App^NL‐F^ KI and Trem2 R47H KI genotype, homozygous mice were used; for Homer1‐eGFP, heterozygous mice were used.

#### Primary neuronal culture

Primary hippocampal neurons were prepared as previously described (Lee *et al*, 2016) from E18 Sprague Dawley rats and Homer1‐eGFP mice of either sex (*n* = 10–15 pups per preparation). Briefly, hippocampi were dissected, dissociated with papain, and triturated with a polished half‐bore pasteur pipette. Next, cells were resuspended in Hank's Balanced Salt Solution (HBSS; HyClone, Logan, UT) supplemented with 0.6% glucose, 1 mM pyruvate, 2 mM GlutaMAX (Gibco), and 10% FBS (HyClone) and plated on Poly‐D‐lysine (PDL)‐coated glass coverslips in a 60‐mm Petri dish or 35 mm glass‐bottom culture dish (81158, ibidi). Four hours after plating, the medium was replaced with neurobasal medium (Invitrogen) supplemented with 2% (v/v) B‐27 (Invitrogen), 0.5 mM GlutaMAX. Half of the medium was replaced by a new neurobasal media with B‐27 and L‐glutamine at DIV 4, 7 and 14. Four mM 1‐β‐D‐cytosine‐arabinofuranoside (Ara‐C; Sigma) was added as needed. Cells were maintained in an incubator at 37°C and 5% CO_2_ and used at DIV 17–21 for experimental procedures.

#### Primary neuron GCaMP7 transfection

Neurons were transfected using a modified calcium‐phosphate method as previously described (Lee *et al*, 2016) using a pAAV‐syn‐jGCaMP7c‐WPRE plasmid (Addgene). Briefly, 6 μg of DNA and 9.3 μl of 2 M CaCl_2_ were mixed in distilled water to a total volume of 75 μl and the same volume of 2× BBS [50 mM BES, 280 mM NaCl, and 1.5 mM Na_2_HPO_4_ (pH 7.1)] was added. The cell culture medium was completely replaced by transfection medium (MEM; 1 mM sodium pyruvate, 0.6% glucose, 10 mM HEPES, 1 mM Kynurenic acid, and 10 mM MgCl_2_, pH 7.71), and the DNA mixture was added to the cells and incubated in a 5% CO_2_ incubator for 60 min. Cells were washed with a washing medium (pH 7.30) and then returned to the original culture medium. Neurons were transfected at DIV 8–9 and analyzed at DIV 16–21. pAAV‐syn‐jGCaMP7c‐WPRE plasmid was purchased from Addgene.

#### Primary microglial culture preparation

Primary mouse WT and Trem2 R47H KI microglial cultures were prepared at P0‐P4 mice from either sex (*n* = 8–10 pups per preparation). Mouse brains were dissected in cold HBSS on ice, and the cortices and hippocampi were isolated. Tissue was homogenized with 2 ml stripette (15 strokes). Next, the homogenate was put through a pre‐wet 70 μM strainer and centrifuged at 400 *g* for 5 min at 4°C. The supernatant was removed, and the cell pellet was resuspended in ice‐cold 35% isotonic percoll. The interface was carefully created with HBSS. The samples were centrifuged for 40 min at 4°C at 2,800 *g* with no break and with slow acceleration and deceleration. The myelin layer and supernatant was aspirated, and the cell pellet was washed in HBSS. The cells were centrifuged for 5 min at 4°C and 400 *g*. The supernatant was removed, and cells were resuspended in 1 ml microglial media (DMEM F12 Gibco, 5% fetal bovine serum Gibco, 1% pen‐strep Gibco, 50 ng/ml CSF1 416‐ML‐010/CF RnD Systems, 50 ng/ml TGFb1 7666‐MB‐005/CF RnD Systems, and 100 ng/ml CX3CL1 472‐FF‐025/CF RnD Systems) for cell counting. Cells were plated in borate buffer 0.1 M pH 8.5 PDL (Gibco)‐coated 12‐well plates (CC7682‐7512, Starlab) in 1 ml of microglial media at a density of 650,000 per well. Cells were maintained in an incubator at 37°C and 5% CO_2_. Ninety percent of media was changed the day after, and subsequently half of the media was changed every 2 days.

Primary microglial cells are supplemented with TGFβ, which has been shown to imprint key microglial signature genes such as *Tmem119* (Butovsky *et al*, 2014). Additionally, mCSF is added to the media to stimulate microglial survival and fractalkine as the ligand for the key microglial homeostatic receptor, CX3CR1. The combination of TGFβ, mCSF, and fractalkine is used to mimic a more homeostatic *in vivo*‐like profile of microglia as shown by high levels of *Tmem119* mRNA in *Cx3cr1* ^+^ *Trem2* ^+^ *Itgam* ^+^ primary microglial cells (Fig EV1C).

#### RNA isolation, reverse transcription, and RT–qPCR

Primary microglial cells were lysed and scraped off using TRIzol reagent (15596026, Invitrogen) after which chloroform was added to separate the homogenate layers. RNA was precipitated from the aqueous layer using 2‐propranolol and then washed with ethanol. The RNA pellet was resuspended in nucleus‐free‐water after which RNA purity and concentration was assessed by Nanodrop. mRNA was converted to cDNA using the qScript cDNA SuperMix reverse transcription kit as described by the manufacturer (95048, Quantabio). For RT–qPCR, 12 ng of cDNA was loaded in triplicates per gene in a total volume of 20 μl using the SYBR green PCR master mix as described by the manufacturer (4309155, ThermoFisher). The reaction was run using a LightCycler 96 Instrument (Roche) with white 96‐well plates (04729692001, Roche). Triplicate Ct values were averaged, and data are shown as respective to the geomean of three housekeeping genes (*Actb*, *Gapdh*, *Rpl32*) using the Ct delta method (2^−∆∆Ct^). Primers purchased from IDT were used at a concentration of 200 nM, see Reagents and Tools table for sequences.

#### Neuron–microglia co‐culture

Primary microglia at DIV 7 were detached with ice‐cold PBS and centrifuged for 5 min at 4°C and 400 *g*. The supernatant was removed, and cells were resuspended in 1 ml neuron culture media supplemented with CSF1, TGFβ1, and CX3CL1. Cells were plated on the DIV 14 neurons at the ratio of 2:1. Cells were co‐incubated in an incubator at 37°C and 5% CO_2_ for 7 days before analysis.

#### Neuronal calcium imaging

To measure neuronal spontaneous calcium activity, GCaMP7‐expressing primary neurons cultured with or without microglia were assessed using a spinning disk confocal microscope (ECLIPSE Ti‐E, Nikon) with a Plan Apo 60×/NA 1.40 oil objective and a Neo sCMOS camera (Andor Technology) at 37°C. Time‐lapse images were acquired every 100 ms for 1 min. First, regions of interest (ROIs) were drawn around individual dendritic spines, and then, relative fluorescence change (Δ*F*/*F*_0_) versus time traces were generated for each ROI. Ca^2+^ transients were identified as changes in Δ*F*/*F*_0_ that were larger than 10% of the baseline intensity_0_.

#### Live‐cell labeling

For live‐cell labeling of externalized phosphatidylserine (ePtdSer), 1 mM PSVue® 550 (P‐1005, Molecular Targeting Technologies; prepared following the manufacturer's instructions) was diluted in Tyrode's solution (136 mM NaCl, 2.5 mM KCl, 2 mM CaCl_2_, 1.3 mM MgCl_2_, 10 mM HEPES and 10 mM Glucose, pH 7.4) at 1:1,000 and incubated for 10 min before imaging. For live‐cell microglial labeling, fluorescent conjugated plant lectin Griffonia (Bandeiraea) simplicifolia lectin I, Isolectin GS‐IB_4_‐647 (IB4; I32450, ThermoFisher) was diluted in Tyrode's solution at 1/1000 and incubated for 10 min before imaging. IB4 binds selectively to microglial RET receptor tyrosine kinase and is commonly used as a microglial marker in the brain.

#### High‐resolution cell imaging and analysis

Airyscan live‐cell images were acquired with a laser scanning LSM880 Airyscan microscope, using a Plan APO 20X/NA 0.8 objective (Zeiss). Emission filter bandwidths and sequential scanning acquisition were set up, to avoid any possible spectral overlap between fluorophores with 37°C and 5% CO_2_ maintained. 3D time‐lapse images were acquired in z‐stack step size 800 nm × 15 steps every 2 min for 1 h and subsequently processed using Imaris software (Bitplane). PSVue^+^ Homer1‐GFP inside the microglia were identified by masking PSVue colocalized Homer1 with IB4 surface. For Homer1‐eGFP and PSVue colocalization analysis, z‐stack images were acquired on a LSM880 Airyscan microscope using 63×/NA 1.40 objective with 0.3 μm z‐steps. The percentage of Homer1 and PSVue colocalization was calculated for every z‐step using Fiji (NIH software). For quantification of preferential contact and engulfment of Homer1‐eGFP^+^ PSVue^+^ dendritic spines by microglia, ROIs with mobile Homer1‐eGFP puncta within 5 μm from microglial were selected and the ratio of colocalization with PSVue was analyzed.

For quantification of PSVue‐level postfixation of neuron‐only and co‐cultured neurons with microglia in GCaMP7 experiments, z‐stack images were acquired on a LSM980 Airyscan microscope using 63×/NA 1.40 objective with 0.17 μm z‐steps. The puncta number per 100 μm^2^ and mean intensity of PSVue were measured per ROI using Fiji (NIH software). For quantification of PtdSer externalization dependent on depolarization and calcium transients, PSVue‐labeled primary neurons were treated with 30 mM KCl or 50 nM oAβ in normal or low‐calcium Tyrode's solution (136 mM NaCl, 2.5 mM KCl, 0.2 mM CaCl_2_, 3.1 mM MgCl_2_, 10 mM HEPES and 10 mM Glucose, pH 7.4). Z‐stack images were acquired before and after the treatment. The puncta number per 100 μm^2^ and mean intensity of PSVue were measured per ROI using Fiji.

#### Intracerebroventricular PSVue injection

For *in vivo* labeling of ePtdSer, 1 mM PSVue® 643 (P‐1006, Molecular Targeting Technologies) was used following the manufacturer's recommendations. Four‐month‐old WT and J20 Tg animals and 6‐month‐old App^NL‐F^ KI and App^NL‐F^ KI; Trem2 R47H KI mice littermates were used. Mice were anesthetized with 4% inhaled Isoflurane (Forane, Abbott Laboratories) and placed in a stereotaxic apparatus (504926, World Precision Instruments Ltd). Anesthesia was maintained at 1.5% in 250 ml/min oxygen flow. Under aseptic conditions, a midline incision was made to reveal the skull. Two holes were drilled in the skull using a 0.8 mm diameter burr (503599, OmniDrill35 Micro Drill, World Precision Instruments Ltd) to allow for bilateral injections into the lateral ventricles. Next, 1.5 μl of sterile PSVue was injected using a 10 μl syringe (NanoFil, World Precision Instruments Ltd) with a fine borosilicate glass capillary (Hamilton) in the following coordinates: 0.5 mm anterior/posterior, ± 1.0 mm lateral, and −2.3 mm dorsal/ventral from bregma (Paxinos and Franklin's The Mouse Brain in Stereotaxic Coordinates, Fourth Edition). Infusion was performed with a Microinjection Syringe Pump (World Precision Instruments Ltd) at a rate of 0.3 μl/min. The needle was kept in this position for an additional 5 min after injection and then retracted slowly to avoid backflow. The incision on the scalp was closed with Vetbond tissue adhesive (3 M). Subcutaneous carprofen (Carprieve, 5 mg/g body weight) and buprenorphine (Vetergesic, 0.1 mg/g body weight) diluted in 0.9% saline were administered peri‐operatively. Twenty‐four hours after injection, the animals were perfused with 4% PFA for histological analysis.

#### Crude synaptosome preparation

Synaptosomes were prepared from fresh mouse and frozen human postmortem brain tissue provided by the Queen Square Brain Bank for Neurological Disorders and the Newcastle Brain Tissue Resource (see Dataset EV1 for patient information). WT mice aged 2–4 months were used (3–5 animals per preparation). In brief, mice were intracardiac perfused with 10 ml cold PBS. The hippocampi and cortices were dissected on ice. For postmortem human tissue, synaptosomes were prepared from the frontal cortex. Synaptosomes were biochemically isolated as previously described (Sodero *et al*, 2011). Tissue was weighed and homogenized in five volumes of sucrose homogenization buffer (5 mM HEPES pH 7.4, 320 mM sucrose, 1 mM EDTA) using a Dounce homogenizer with 15–20 strokes. The homogenate was centrifuged at 3,000 *g* for 10 min at 4°C, and the supernatant was saved as total homogenate fraction (THF). The THF was centrifuged again at 14,000 *g* for 12 min at 4°C, and supernatant was saved as cytosolic fraction. The pellet was carefully resuspended in 550 μl of Krebs‐Ringer buffer (KRB: 10 mM HEPES, pH 7.4, 140 mM NaCl, 5 mM KCl, 5 mM glucose, 1 mM EDTA) and 450 μl of Percoll solution (for a final concentration of 45%). The solution was mixed by gently inverting the tube, and an interface was slowly created with KRB. After centrifugation at 14,000 *g* for 2 min at 4°C, the synaptosomal fraction was recovered at the surface of the flotation gradient and carefully resuspended in 1 ml of KRB to wash. The synaptosomal preparation was centrifuged at 14,000 *g* for 1 min at 4°C, after which the pellet was resuspended in KRB. When done on fresh tissue, this protocol yields synaptosomes that are electrically functional for several hours post‐isolation as they can be depolarized and stimulated with KCl and NMDA, respectively. After isolation, a standard BCA protein assay was performed to quantify the amount of protein for subsequent assays.

All human samples used in this study were tested for the presence of Aβ on isolated synaptosomes by western blotting as described below to confirm AD pathology status. Results are clearly stated in Dataset EV1. NDC and AD cases used for our experiments are clearly negative or positive, respectively, for Aβ immunoblotting.

#### Synthetic humanized Aβ oligomer 40‐S26C dimer treatment

Primary cultures or fresh synaptosomes were treated with 50 nM Aβ oligomer 40‐S26C dimer (018–71, Phoenix) versus PBS control for 1 h in an incubator at 37°C and 5% CO_2_. Experimental procedures were either performed on live cells during the 1‐h window or on fixed cells post‐treatment. Fresh mouse synaptosomes were immediately divided into Eppendorfs at 2–2.5 mg of protein and resuspended in total 1 ml KRB in 50 nM of oAβ 40‐S26C dimer or just buffer and PBS as control and left overnight at 4°C on nutator. Synaptosomes were then centrifuged at 14,000 *g* for 1 min at 4°C, supernatant was discarded, and synaptosomes were washed in 1 ml PBS, after which they were centrifuged at 14,000 *g* for 1 min at 4°C to obtain oAβ‐synaptosomes and control synaptosomes.

#### Immunocytochemistry (ICC)

Cells and synaptosomes were fixed for 10 min at room temperature (RT) in 4% (w/v) PFA, 4% (w/v) sucrose in PBS, pH 7.4 and subsequently permeabilized with 0.25% Triton X‐100 in PBS for 3 min at RT. The cells were then blocked for 1 h at RT in 10% (w/v) Bovine serum albumin (BSA). Cells and synaptosomes were incubated at 4°C overnight in primary antibodies (1/1000) after which the cells were washed in PBS and incubated with secondary antibodies (1/1000) for 1 h at RT (see Reagents and Tools table for antibody information). The immunostaining of synaptosomes was performed in Eppendorfs with a centrifugation step at 14,000 *g* for 1 min at every wash step. At the end of the protocol, synaptosomes were resuspended in ProLong Gold Antifade mounting media (P36930, Invitrogen) and put through a 1 ml insulin syringe to further homogenize synaptosomes. This solution was then mounted on glass slides (SuperFrost GOLD Adhesion Slides 11976299, Fisher Scientific).

For fresh synaptosome labeling of ePtdSer, synaptosomes were left on nutator at RT in PSVue 643 for 1 h after which excess PSVue was centrifuged at 14,000 *g* for 1 min and washed. Synaptosomes were then fixed for ICC.

#### Synaptosomes conjugation to pHrodo amine‐reactive labels

Synaptosomes were conjugated to low‐background pH‐sensitive dyes, which fluoresce brightly upon acidification (pH 4–6) such as in late endosomes and lysosomes using an adapted protocol from (Lehrman *et al*, 2018). pHrodo dyes are photostable allowing for multicolor and long‐term imaging. In brief, pHrodo™ Red, succinimidyl ester (P36600) and pHrodo™ Deep Red Antibody Labeling Kit (P35355) were dissolved as described in the manual. Human postmortem synaptosomes were conjugated to pHrodo red, whereas mouse synaptosomes were conjugated to both pHrodo red and deep red for preferential engulfment studies. One mg of synaptosomes was left at RT on nutator for 2 h in sodium bicarbonate 0.1 M with respective pHrodo at a concentration of 1 mg/ml. After conjugation, synaptosomes were centrifuged at 14,000 *g* for 1 min, then washed with 1 ml PBS and centrifuged again at 14,000 *g* for 1 min. Synaptosomes were then resuspended as described by the manufacturer. Next, a standard BCA protein assay was performed on pHrodo‐conjugated synaptosomes to quantify protein concentrations for subsequent engulfment assays. Synaptosomes were then aliquoted, respectively, and stored at −80°C. Prior to engulfment analysis, the degree of pHrodo labeling (DOL) was assessed as described by the manufacturer. In brief, synaptosomes were mixed 1:3 in PBS pH 2 to activate the fluorophores. The relative efficiency of the labeling reaction was determined by measuring the absorbance of the protein at 280 nm and the absorbance of the dye at its excitation maximum using a UV–Vis Spectrophotometer. This was done as a control to ensure that the DOL was similar between pHrodo and treatment paradigms.

#### Synaptosome electron microscopy

Synaptosome pellet was pooled from the hippocampi and cortices of three wild‐type mice and prepared as described above. The pellet was fixed in 2% glutaraldehyde/1.5% formaldehyde in 0.1 M sodium cacodylate for 30 min. Postfixation, the fix was removed and replaced with 1% osmium/1.5% potassium ferricyanide for 1 h at 4°C. Postosmium treatment the sample was spun down in a microfuge, the osmium solution was removed and the sample washed three times in cacodylate buffer, mixing and centrifuging each time prior to removing the wash buffer. The samples were resuspended in 0.1% tannic acid and then dehydrated in 70, 90, and 100% ethanol. To maintain pellet integrity, a 1:1 mix of propylene oxide and epon resin was added. After 1 h, the tubes were spun for 3 min in the centrifuge and the po:epon mix removed before 100% resin was added. Samples were left overnight in resin to ensure proper infiltration, after which the old resin was removed and replaced with freshly prepared resin. Samples were left for 4 h and transferred to the oven to polymerize overnight.

#### *In vitro* microglia‐synaptosome engulfment assay

For preferential *in vitro* engulfment assays using mouse synaptosomes, primary mouse microglia were treated with oAβ‐synaptosomes and control synaptosomes. One μg of both control and oAβ‐synaptosomes was added to the same well in microglial media. For engulfment assays using human postmortem synaptosomes, primary mouse microglia were either treated with 1 μg of Alzheimer's disease (AD) or non‐demented control (NDC) synaptosomes in separate wells. Plates were then placed in a cell discoverer 7 (CD7) with the incubator at 37°C and 5% CO_2_. Fluorescent (594 nm and 647 nm) and bright‐field (oblique and phase) images were acquired at a ×20 objective (×0.5) at intervals of 2–5 min. Laser settings were set to the same settings for both red and deep red pHrodo. A 3‐slice z‐stack was taken at 1.5‐μm interval to ensure that imaging was within focus throughout the imaging session; however, one plane was used for analysis. Two ROIs per well were taken with an average of 40 cells per ROI. An imaging session lasted 0–15 h, whereby plateau was reached within the first 6 h, with *t* = 0 being the addition of synaptosomes. The plateau phase persisted, and a decrease in the pHrodo signal was only observed after 48–72 h. Background subtraction was performed on ImageJ for red and deep red pHrodo at 1 pixel. For analysis, a plug‐in on ImageJ was used, z‐profile axis, which measures intensity of a given channel with respect to time. Fluorescence intensity at *t* = 0 was subtracted from subsequent time frames. Experimental replicates were analyzed separately. Data were either shown as fluorescence intensity with time, engulfment (area under curve (AUC) at 50% of the peak pHrodo fluorescence intensity), or engulfment ratio. AUC at 50% peak pHrodo intensity for all experiments was approximately at 3 h. For AUC at 50%, pHrodo intensity was normalized to average control. For engulfment ratio, the sum of control and oAβ‐synaptosome fluorescence intensity was added per well and then individually divided by this total sum, to obtain a fraction per well. For dextran engulfment, pHrodo™ Red Dextran, 10,000 MW, for Endocytosis (P10361, ThermoFisher) was used following the manufacturer's instructions and imaged on the CD7.

#### Annexin‐V treatment

Synaptosomes and neuron–microglia co‐cultures were treated with Annexin‐V, a protein that specifically binds to ePtdSer with high affinity, which has been used to mask PtdSer on apoptotic cells to block macrophage phagocytosis (Krahling *et al*, 1999). Synaptosomes were pretreated with either buffer, 1 μg/ml or 10 μg/ml of purified recombinant Annexin V (556416, BD BioSciences, 0.5 mg/ml stock concentration) in 100 μl 1× Annexin‐V binding buffer (556454, BD Biosciences), 0.1 M HEPES (pH 7.4) 1.4 M NaCl, 25 mM CaCl_2_ (Annexin‐V binding to ePtdSer is calcium‐dependent) for 1 h at RT. These concentrations have been previously used in literature and suggested by the manufacturer (5–15 μg). Synaptosomes were centrifuged at 14,000 *g* for 1 min to remove excess Annexin‐V and resuspended in 1× Annexin‐V binding buffer. Control and oAβ‐synaptosomes were pretreated simultaneously in the same Eppendorf, whereas NDC and AD human synaptosomes were pretreated separately. Neuron–microglia co‐cultures were treated with 0.1 μg/ml of Annexin‐V simultaneously with Aβ treatment.

#### Bafilomycin A1 treatment

Postmicroglia‐synaptosome engulfment assays, 50 nM bafilomycin A1 (B1793, Sigma‐Aldrich), which increases lysosomal pH to 6, was added to microglial wells. Imaging and analysis were performed as described above. Background subtraction was performed at all time points using the intensity at *t* = 0 (when synaptosomes were added). Data shown as *t* = 0 when bafilomycin treatment was added.

#### Western blotting

A BCA protein assay was used to determine the amount of protein in fractions used for western blot analysis. Twenty–forty micrograms of protein was loaded either on 4–12% Bis‐Tris gels or 10% Tris‐Glycine gels (Thermo Fisher Scientific). Gels were run with appropriate sample buffer and running buffer. Gels were transferred to nitrocellulose membranes using an iBlot 2 Dry Blotting System (Thermo Fisher Scientific) as described by the manufacturer. Prior to blocking, Aβ blots were left in PBS and microwaved at 80% strength for 1 min 30s on each side, allowing for 3 min 30 s to settle after each side to expose the epitope. Blots were blocked in casein PBS (1:1, Biorad) for 30 min at RT on shaker and then probed overnight at 4°C on shaker with primary antibodies (1:1,000) in casein PBS‐T (0.01%). Blots were washed, then probed with secondary antibodies in casein PBS‐T (0.01%) for 1 h at RT on shaker, and then visualized either fluorescently on a ChemiDoC (BioRad) system or with HRP‐substrate on an Amersham Imager 680 (Bioke) system.

#### Immunohistochemistry (IHC)

IHC was performed on brain slices from mice transcardially perfused with 4% PFA and postfixed for 24 h. Thirty micrometers free‐floating tissue sections were washed in PBS on a perturbator followed by pretreatment in 1% Triton X‐100 in PBS for 20 min, rinsed in PBS for 5 min, and treated with 0.3% Triton X‐100. Blocking solution and primary and secondary antibody mixtures were centrifuged at 17,000 *g* for 5 min just before use. Sections were then blocked in 20% NGS, 1% BSA, and 0.3% Triton in PBS for synapse IHC or in 10% NGS, 10% FBS, 1% BSA, and 0.3% Triton in PBS for microglial engulfment IHC for 2 h followed by primary antibody incubation overnight at 4°C. Sections were washed in PBS for 10 min, 0.3% Triton X‐100 in PBS for 30 min, followed by secondary antibody incubation for 4 h at room temperature. See Reagents and Tools table for antibody information. Sections were then washed in PBS for 10 min, incubated in 1:10,000 DAPI in PBS for 10 min, and washed in 0.3% Triton for 15 min. Finally, sections were mounted onto slides with ProLong Glass mounting medium and procured for at least 48 h before imaging. IHC of PSVue‐labeled tissue was as described above, with the following substitutions: TBS instead of PBS; Alexa Fluor 546 goat anti‐rabbit instead of Alexa Fluor 594 goat anti‐rabbit.

#### *In situ* hybridization (RNAScope)

RNAScope was performed following the manufacturer's instructions using the RNAscope Fluorescent Multiplex Assay (ACDBio, 320293). The following probes were purchased from the manufacturer: *Tmem119* (472901‐C3), *C1qa* (441221‐C2), and *Clec7a* (532061‐C1). In brief, mice were perfused with 4% PFA and the brains were postfixed in 4% for 24 h. Twelve‐μm‐thick sections were mounted on Superfrost Plus GOLD Slides (Thermo Fisher Scientific, K5800AMNZ72). The slides were incubated in H_2_O_2_ for 4 min at RT and washed in RNAse‐free water. Slides were then placed in boiling target antigen retrieval for 4 min, dehydrated in 100% ethanol for 5 min, and treated with Protease Plus for 15 min at RT before probe hybridization for 2 h at 40°C. For IHC staining post‐RNAScope, the slides were blocked in 2% serum 0.01% Triton X for 30 min and then incubated with the primary antibodies (1/100) overnight at 4°C. The following day, the slides were washed and incubated with the secondary antibodies for 2 h at RT. The blocking buffer was used to dilute the antibodies. Slides were then incubated with DAPI and mounted.

#### *In vivo* microglial engulfment imaging and analysis

Images were acquired on a Zeiss LSM 800 confocal microscope using 63× magnification. Frame size of 2,048 × 2,048 was used for all images. Eleven micrometer Z‐stack was acquired with a voxel size of 0.05 × 0.05 × 0.220 μm^3^. Three regions of interest were acquired in the hippocampal CA1 stratum radiatum per section, and three sections were analyzed per brain. Images were first processed with ImageJ using background subtraction. Imaris 3D surface rendering was used to first create a surface on the P2Y12 channel. Subsequently, CD68 channel was masked to the P2Y12 surface to obtain only CD68 within the analyzed cell. A surface was created on the CD68 masked channel, and a volume filter of > 0.01 μm^3^ was applied. Homer1 channel was then masked to the CD68 surface to obtain Homer1 inside CD68^+^ vesicles, and a surface was created on this masked Homer1 channel. Homer1 surfaces of volume smaller than 0.001 μm^3^ were filtered out and not included in the analysis. Volume of thus obtained Homer1‐immunoreactive puncta was then normalized per cell volume to account for variance due to difference in cell size. Imaris 3D surface function algorithm settings were the following for all channels: “Shortest distance calculation,” “Absolute intensity,” and grain size of 0.1 μm were used. The threshold values were adjusted to optimize visualization of individual channels and varied between channels, but were kept constant within a channel for all images analyzed. Data shown as engulfment index: (volume of Homer1 in CD68/microglial cell volume) * 100.

#### Super‐resolution imaging and analysis

Super‐resolution synapse images were acquired on a Zeiss LSM 880 microscope with Airyscan detector using a 63×, 1.4NA oil immersion Plan‐Apochromat objective (theoretical maximum resolution: 140 nm lateral, 350 nm axial). A zoom factor of 1.8× and frame size of 2,048 × 2,048 was used for all images, resulting in an XY pixel size of 37 nm. Z‐step size was 144 nm, with eight steps per Z‐stack, resulting in a stack thickness of 1.15 μm. Scan speed was 5, line averaging 2, gain 800, and digital gain 1. A laser power of ~0.15 and ~0.45% was used for the 488 and 594 nm lasers, respectively. The Airyscan detector was aligned before imaging each new slide. Three regions of interest were acquired in the center of the hippocampal CA1 stratum radiatum for each brain section. Super‐resolution synapse images were processed in Zen Black using 3D Airyscan Processing at strength 6.0. Synapse quantification method was adapted from Hong *et al*, 2017. Images exhibiting drift or anomalous staining were excluded from analysis. Imaris software was used for pre‐ and postsynaptic puncta detection. Channel brightness was adjusted to maximize the visualization of immunoreactive puncta. The spot detection function was used to generate spots, with region growing, shortest distance calculation, and background subtraction enabled. Spots size was selected to maximize the detection of immunoreactive puncta (XY diameter 0.15 μm, Z diameter 0.45 μm, for both Homer1 and Synaptotagmin 1/2 puncta). Spots were classified using the “Intensity Center” filter. The threshold value was selected to maximize the detection of immunoreactive puncta. The threshold value varied between channels and immunostainings but was kept consistent between pairs of images that were compared. Local contrast was used to define the spot growth boundary, using a value that appropriately reflected the immunoreactive signal of the source channel. A volume filter was then applied to remove spots smaller than 0.005 μm^3^. Pre‐ and postsynaptic spots were colocalized using a MATLAB colocalization script (Colocalize Spots XTension), using a colocalization distance of 0.25 μm between spot centers. Synapse density is shown as App^NL‐F^ KI mice normalized to WT mice, and App^NL‐F^ KI; *Trem2* R47H KI normalized to *Trem2* R47H KI mice.

PSVue‐labeled synapse images were acquired on a Leica Stellaris STED microscope using a 100×, 1.4NA oil immersion Plan‐Apochromat objective. A zoom factor of 2× and frame size of 2,048 × 2,048 was used for all images, resulting in an XY pixel size of 29 nm. Z‐step size was 200 nm, with 11 steps per Z‐stack, resulting in a stack thickness of 2.2 μm. Images were acquired in Photon Counting mode with a scan speed of 600 Hz and line accumulation 8. A laser power of 5, 5, and 10% was used for the 499, 557, and 653 nm lasers, respectively. Three regions of interest were acquired in the center of the hippocampal dentate gyrus hilus for each brain section. Confocal images of PSVue‐labeled synapses were deconvolved using Leica's LIGHTNING deconvolution. Imaris was used for pre‐ and postsynaptic puncta detection as described above. The surface function was then used to generate volumes representing the PSVue signal. Pre‐ and postsynaptic spots within 0.25 μm of a PSVue surface were determined using a MATLAB colocalization script (Spots Close To Surface XTension).

#### Statistics

Data curation of image analysis was performed by investigators blinded to the genotype or treatment of animals/cultures. Statistical tests were performed using GraphPad Prism 9.0 (GraphPad Software) as appropriate. Data points are shown as ROIs and average or median per experimental replicate (either per animal or culture preparation) where applicable. Statistical tests have been performed per animal for mouse studies and per experimental replicate for cell culture studies, with the exception of GCaMP studies which are done per individual spine in accordance with the field's standards. Data shown as mean ± SEM or SD. *P*‐values shown as not significant (ns) *P* > 0.05; *P < 0.05; **P < 0.01; ***P < 0.001; ****P < 0.0001. Statistical tests and data representation are stated in the figure legends.

## Author contributions

**Javier Rueda‐Carrasco:** Conceptualization; data curation; formal analysis; supervision; validation; investigation; visualization; methodology; writing – original draft; writing – review and editing. **Dimitra Sokolova:** Conceptualization; data curation; formal analysis; funding acquisition; validation; investigation; visualization; methodology; writing – original draft; writing – review and editing. **Sang‐Eun Lee:** Conceptualization; data curation; formal analysis; funding acquisition; validation; investigation; visualization; methodology; writing – original draft; writing – review and editing. **Thomas Childs:** Data curation; formal analysis; validation; investigation; visualization; methodology. **Natália Jurčáková:** Data curation; formal analysis; investigation; visualization. **Gerard Crowley:** Investigation. **Sebastiaan De Schepper:** Investigation. **Judy Z Ge:** Investigation. **Joanne I Lachica:** Resources. **Christina E Toomey:** Resources. **Oliver J Freeman:** Supervision; funding acquisition. **John Hardy:** Conceptualization. **Samuel J Barnes:** Methodology. **Tammaryn Lashley:** Resources. **Beth Stevens:** Conceptualization. **Sunghoe Chang:** Supervision; funding acquisition. **Soyon Hong:** Conceptualization; supervision; funding acquisition; visualization; methodology; writing – original draft; project administration; writing – review and editing.

## Disclosure and competing interests statement

OJF is employed by AstraZeneca. The following patents have been granted or applied for PCT/2015/010288, US14/988387, and EP14822330 (SH). All the other authors declare that they have no competing interests.

## Supporting information



Appendix S1

Expanded View Figures PDF

Movie EV1

Movie EV2

Movie EV3

Movie EV4

Movie EV5

Movie EV6

Dataset EV1

PDF+

Source Data for Figure 1

Source Data for Figure 2

Source Data for Figure 3

Source Data for Figure 4

## Data Availability

This study includes no data deposited in external repositories.
