# Author Correction: Microglia-synapse engulfment via PtdSer-TREM2 ameliorates neuronal hyperactivity in Alzheimer’s disease models

**DOI:** 10.1038/s44318-024-00159-5

**Published:** 2024-07-31

**Authors:** Javier Rueda-Carrasco, Dimitra Sokolova, Sang-Eun Lee, Thomas Childs, Natália Jurčáková, Gerard Crowley, Sebastiaan De Schepper, Judy Z Ge, Joanne I Lachica, Christina E Toomey, Oliver J Freeman, John Hardy, Samuel J Barnes, Tammaryn Lashley, Beth Stevens, Sunghoe Chang, Soyon Hong

**Affiliations:** 1 UK Dementia Research Institute, Institute of Neurology, University College London, London, UK; 2 Neuroscience BioPharmaceuticals R&D, AstraZeneca, Cambridge, UK; 3 Department of Physiology and Biomedical Sciences, Seoul National University College of Medicine, Seoul, South Korea; 4 Department of Neuroscience, Physiology and Pharmacology, University College London, London, UK; 5 The Queen Square Brain Bank for Neurological Disorders, UCL Queen Square Institute of Neurology, London, UK; 6 Department of Clinical and Movement Neurosciences, UCL Queen Square Institute of Neurology, London, UK; 7 UK Dementia Research Institute, Department of Brain Sciences, Imperial College London, London, UK; 8 Department of Neurodegenerative Diseases, UCL Queen Square Institute of Neurology, London, UK; 9 F.M. Kirby Neurobiology Center, Boston Children’s Hospital, Boston, MA USA; 10 Harvard Medical School, Boston, MA USA; 11 Stanley Center for Psychiatric Research, Broad Institute of MIT and Harvard, Cambridge, MA USA; 12 Howard Hughes Medical Institute, Boston Children’s Hospital, Boston, MA USA

## Abstract

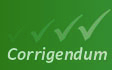

**Correction to:** *The EMBO Journal* (2023) 42:e113246. 10.15252/embj.2022113246 | Published online 14 August 2023

**Figure 3H and 4E statistical analysis are corrected**.

**Figure 1I is corrected**.

**Figure 2A is corrected**.

**Figure 1D and Figure 3B are corrected**.

**Source data for Figure** **1C**, **1D**, **3B** **and** **4E** **is published with this correction**.

The Journal contacted the authors after becoming aware of potential statistical errors in the paper. The authors conducted an internal analysis of the manuscript. Based on the exchanges with the authors, and the provided statistical data, following peer review, the Journal corrects the following figures. All relevant source data is published with this correction notice.

Author Statement.

**Figure 3H and 4E:**

Both figures relate to analysis of synaptic marker quantification via AiryScan super-resolution confocal microscopy and Imaris software. In both figures, the aim was to address whether Trem2-R47H genetic background makes a difference in the ability of the hAPP-NLF mutation to induce synapse loss (Fig. 3H: colocalization of Synaptotagmin1/2- and Homer1-immunoreactive synaptic puncta density; Fig. 4E: Bassoon‐immunoreactive synaptic puncta density). To address this, we first measured synapse numbers in hippocampus of APP mouse model (i.e., hAPP-NLF mice vs. age- and sex-matched WT mice), then in a second set of experiments, we measured synapse numbers in NL-F;Trem2-R47H mice vs. age- and sex-matched Trem2-R47H mice. Hence, we designed the experiments in a paired method, and we compared the effect of hAPP-NL-F mutation normalized to their own genetic background.

For statistical analysis for these two figures, we originally used two-way ANOVA. The essence of post hoc comparisons following ANOVA requires a significant Interaction. ANOVA for Fig. 3H did have a significant interaction, so we applied a post hoc analysis. But ANOVA for Fig. 4E did not have a significant interaction, so it was incorrect for us to apply a post hoc analysis. When we realized that we had made an error for Fig. 4E statistical test, we consulted a biostatistician (who is independent to all co-authors’ labs) to ensure we are performing the most appropriate statistical tests for the experiments in our paper. During this consultation, we were advised that two-way ANOVA was in fact not the appropriate statistical test for Fig. 3H and Fig. 4E in the first place. This is because our paired experimental design for these two figures which contains inherent technical limitations and normalized data points to respective non-APP mice, violate the a priori assumptions of two-way ANOVA (i.e., independence of observations). Instead, the most appropriate statistical test for these two figures is multiple unpaired t-test (with a further correction for multiple comparisons using Bonferroni-Dunn).

To clarify: All experiments for Fig. 3H and Fig. 4E were performed in a paired manner, i.e., measuring the dependent variable (i.e., synapse number) in response to one independent variable (i.e., hAPP-NLF mutation). We already knew from previous experiments and published literature that there are less co-localized synapse numbers in hAPP-NLF mice versus sex-matched WT littermates. We assess synapse numbers in NLF vs. WT mice in one set of experiments, where brains of App^WT^ and App^NL-F^ mice (both mice on Trem2-CV background) were sectioned, immunostained, and imaged on the same slides using super-resolution AiryScan confocal imaging side-by-side with experimenters blinded to genotype. The imaging and quantification are described in detail in the “*Super‐resolution imaging and analysis*” method section in the published paper. In order to remove the technical inter-slide, inter-day, and inter-experiment variability, each transgenic animal was paired with its respective control animal (sex-matched littermates) for all experimental procedures and analysis was performed on 3D Imaris software, with experimental still blinded to genotype, and the result for the experimental condition (hAPP-NLF mutation) was normalized with respect to the result from the control mouse of each pair. Then in a separate set of experiments, we asked whether hAPP-NLF mutation can still induce synapse loss in Trem2-R47H background. We performed another set of paired experiments and analysis but this time in App^WT^ and App^NL-F^ mice where both groups of mice were on the Trem2-R47H genetic background (i.e., App^WT^; Trem2^R47H^ vs. App^NL-F^; Trem2^R47H^).

Hence, given that this technically paired experimental design produces data that can strictly not be seen as independent observations (given the data points are normalized to the respective non-APP mice), it is most appropriate to perform a statistical test as two separate experiments, with 1 variable (APP mutation), i.e., multiple unpaired t-tests. To reduce the likelihood of a type I error, we also applied the Bonferroni-Dunn multiple comparison correction on the acquired *p*-values.

With the corrected statistical test, the analysis of the data comparisons now result to:

**Figure 3H:**
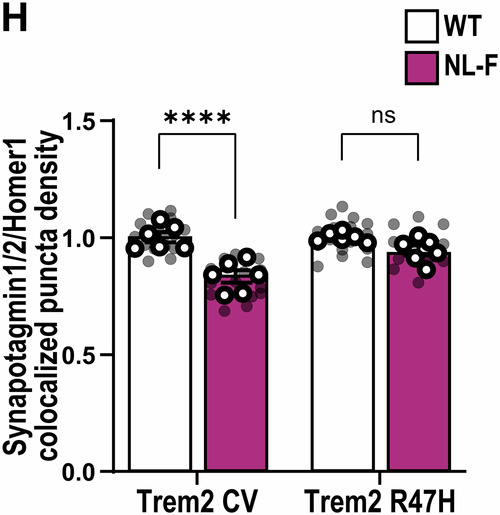
**Original**.

**Figure 3H:**
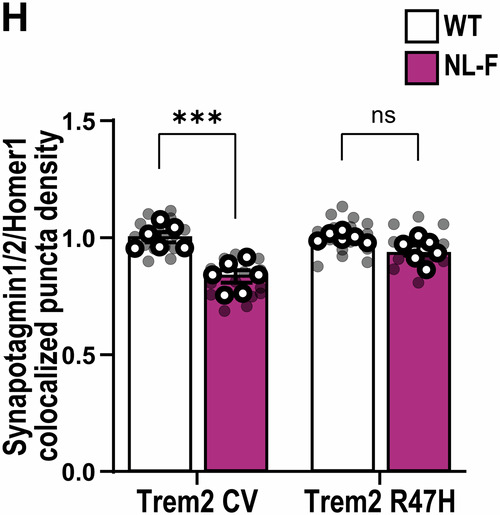
**Corrected**.

**Figure 4E:**
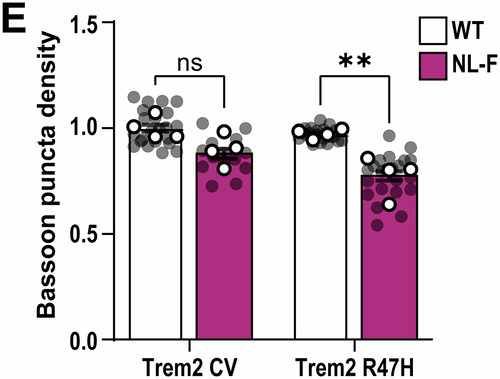
**Original**.

**Figure 4E:**
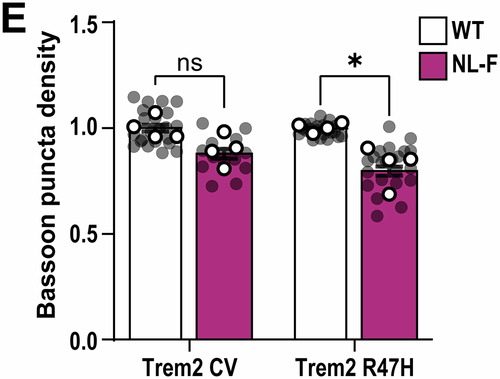
**Corrected**. Source data are available online for this figure.

Hence, with the corrected tests, the interpretations for both Fig. 3H and Fig. 4E are consistent to what were published. We therefore conclude that the reanalysis of these two figures does not affect the conclusions of the paper.

**Figure 1C, 1D and Figure 3B:**

Figures 1C, 1D and 3B share one Source data file (Fig. 1C). We have 20 paired data points for pHrodo-fluorescence intensity with time for in vitro engulfment paradigm for engulfed pHrodo-labeled Control-synaptosome (Ctrl-SN) vs. Abeta-synaptosome (oAβ-SN). We found that 1 paired data point was accidentally duplicated during the transfer process from ImageJ to an Excel sheet.

To clarify: 1 set of data reflects 1 paired time-course experiment comparing the fluorescence intensity coming from engulfed pHrodo-labeled oAβ-synaptosomes vs. engulfed pHrodo-labeled control-synaptosomes inside primary mouse microglia in wells. The data was acquired using CD7 microscopy then using Plot Z-axis profile plug-in on ImageJ, which gives a measure of channel intensity at every given time frame. Each experiment is analyzed per batch, i.e., multiple ROIs and wells are analyzed simultaneously. The analysis then is self-automated; images are acquired and then batch processed using this plug-in which automatically generates multiple individual columns. Each column is then manually copied and pasted onto an Excel sheet. It is during this transfer of data onto Excel sheet that we accidentally duplicated one paired ROI. Specifically, Column O has been duplicated to Column Q for control-synaptosome, and the respective pairing of experiment for oAβ-synaptosome was Column AI to Column AK.

**Figure 1C:**
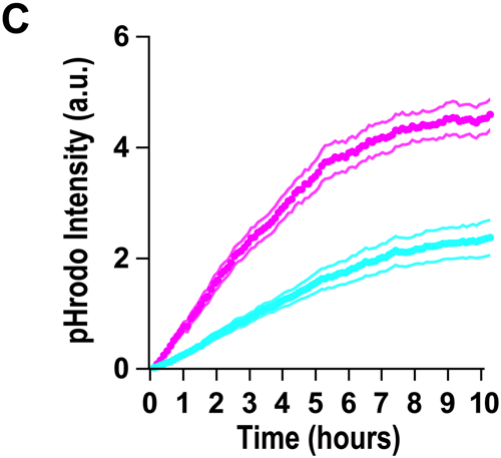
**Original**.

**Figure 1C:**
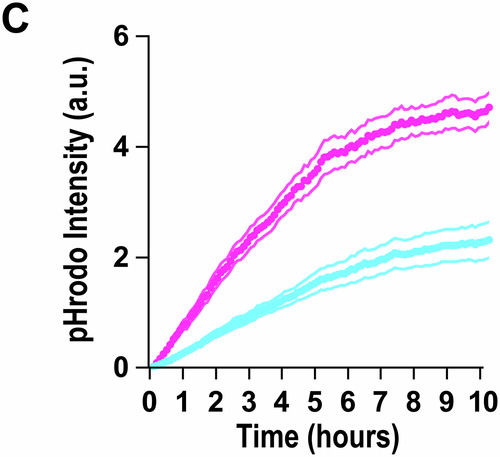
**Corrected**. Source data are available online for this figure.

**- Figure 1C**

Figure 1C displays the mean of those values. Due to the high number of data points, removing one duplicate does not substantially change the presentation of the graph.

**Figure 1D:**
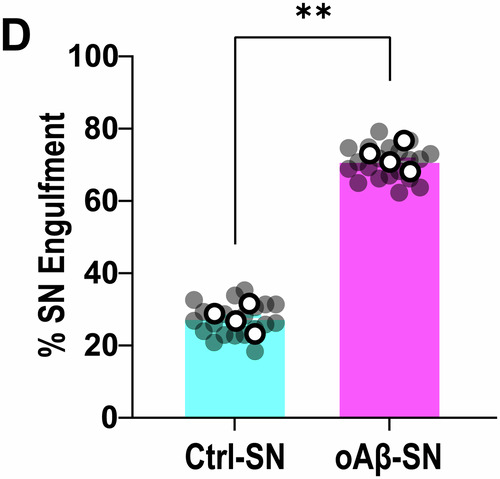
**Original**.

**Figure 1D:**
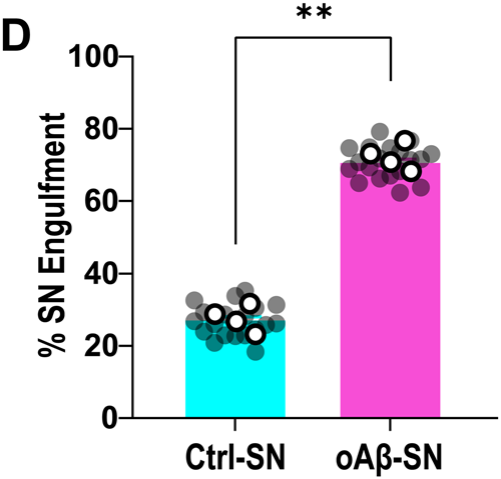
**Corrected**. Source data are available online for this figure.

**- Figure 1D**

Changes are very minor due to the large number of data points; they are best reflected by the gray dots.

**Figure 3B:**
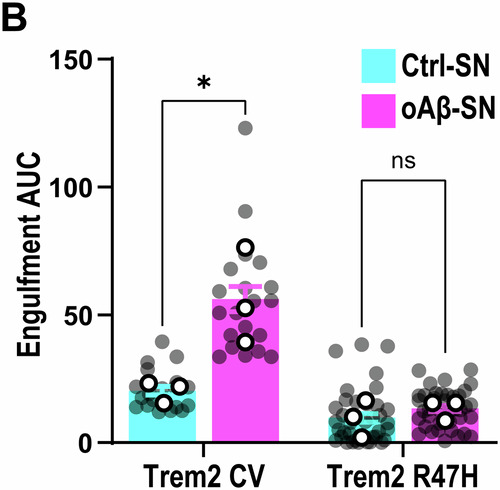
**Original**.

**Figure 3B:**
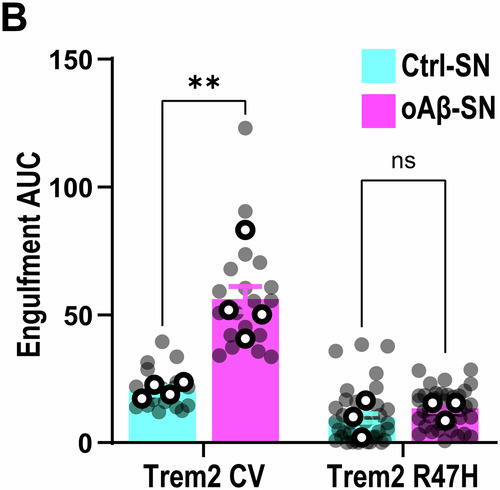
**Corrected**. Source data are available online for this figure.

**- Figure 3B**

These data reflect area under curve using the source data from Figure 1C. We made an error as experiments were performed 4 times, not 3 times. The significance/non-significance of changes still remain intact, although now from *P* < 0.05 to *P* < 0.01 between Ctrl-SN vs. oAβ-SN in Trem2 sufficient mice.

We also found other minor mistakes during our analysis of the published manuscript and apologize for overlooking these errors prior to publication.

**Figure 1I:**
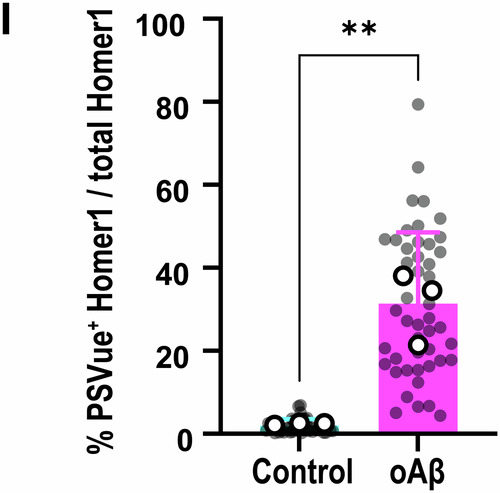
**Corrected**.

**Figure 1I:**
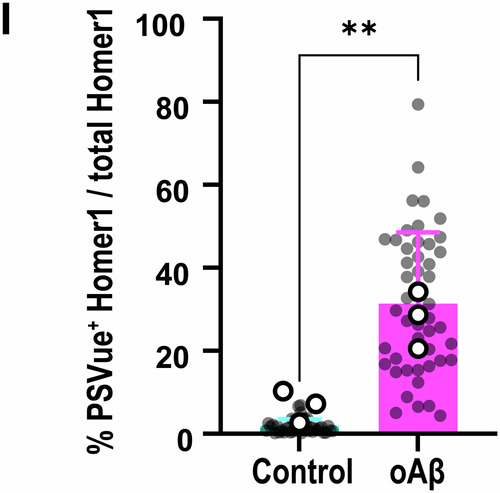
**Original**.

**Figure 1l**

We found that the data points reflecting the means of each independent experiment (open circles) were not correctly represented. Everything else in the figure, including ROIs and stats, are correct.

**Figure 2A:**
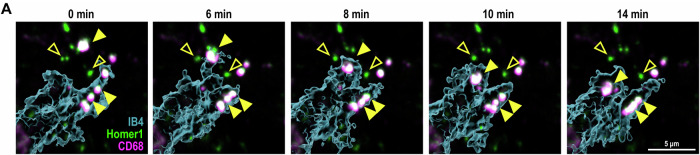
**Original**.

**Figure 2A:**
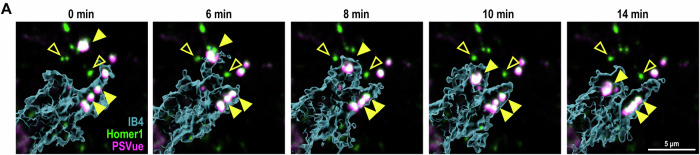
**Corrected**.

**Figure 2A**

We found we have mislabelled the magenta in the figure, it should be PSVue, not CD68. Figure legend, text, and others are correct.

**Corrections in the figure legends**.

**- Figure 1E**, missing ROI information (stated above but to avoid confusion):

‘pHrodo fluorescence with time shown as AUC at 3 h normalized to respective control. AUC of engulfed mouse oAβ-SN versus Ctrl-SN with and without AnnxV pretreatment’

Is corrected to: (Changes in bold)

‘pHrodo fluorescence with time shown as AUC at 3 h normalized to respective control. AUC of engulfed mouse oAβ-SN versus Ctrl-SN with and without AnnxV pretreatment. ***∼40 microglia per ROI, two ROIs per well, 2–3 wells per experiment, n = 3 independent experiments’***

**- Figure 3B**, updating ROI information:

‘pHrodo fluorescence with time shown as AUC at 3 h. AUC of oAβ-SN is higher compared with Ctrl-SN in Trem2 CV but not in Trem2 R47H KI microglia. *∼40 microglia per ROI, two ROIs per well, 2–3 wells per experiment, n = 3 independent experiments’*

Is corrected to: (Changes in bold)

‘pHrodo fluorescence with time shown as AUC at 3 h. AUC of oAβ-SN is higher compared with Ctrl-SN in Trem2 CV but not in Trem2 R47H KI microglia. ***∼40 microglia per ROI, two-three ROIs per well, 2–5 wells per experiment, n = 3–4 independent experiments***’

**- Figure 3** data information, updating statistical test information:

‘Data information: Data shown as mean ± SEM. Each shaded point represents one ROI, and each open point represents the mean of each independent experiment. Two-way ANOVA followed by Bonferroni’s post hoc test. *P*-values shown as ns *P* > 0.05; *P < 0.05; ***P < 0.001; ****P < 0.0001.’

Is corrected to: (Changes in bold)

‘Data information: Data shown as mean ± SEM. Each shaded point represents one ROI, and each open point represents the mean of each independent experiment. Two-way ANOVA followed by Bonferroni’s post hoc test **(B, C and F), or Multiple t-test comparisons corrected by Bonferroni-Dunn method (H)**. *P*-values shown as ns *P* > 0.05; **P **<** **0.01**; ***P < 0.001.’

**- Figure EV5** data information, updating statistical test information:

‘Data information: Data shown as mean ± SEM. Each shaded point represents one ROI, and each open point represents the average per experimental replicate. Two-way ANOVA followed by Bonferroni’s post hoc test. *P*-values shown as ns *P* > 0.05; *P < 0.05; **P < 0.01.’

Is corrected to: (Changes in bold)

‘Data information: Data shown as mean ± SEM. Each shaded point represents one ROI, and each open point represents the average per experimental replicate. **Unpaired t-test (B–D and F–H) or one-way ANOVA followed by Bonferroni’s post hoc test (I)**. *P*-values shown as ns *P* > 0.05; *P < 0.05; **P < 0.01.’

**- Figure 4** data information, updating statistical test information:

Data information: Data shown as mean ± SEM. Each shaded point represents one ROI, and each open point represents the mean (or median for GCaMP studies) of each independent experiment. Central bands of the violin plot (D) represent median and quartiles. The top and the bottom of the box plot (H) represent the 75th and 25th percentiles, respectively, and the line represents the median. The whiskers represent the highest and lowest values that are not outliers. Unpaired t-test (B), Kruskal–Wallis test followed by Dunn’s test (D), two-way (E), or one-way (H) ANOVA followed by Bonferroni’s post hoc test. *P*-values shown as ns *P* > 0.05; *P < 0.05; **P < 0.01, ****P < 0.0001.

Is corrected to: (Changes in bold)

Data information: Data shown as mean ± SEM. Each shaded point represents one ROI, and each open point represents the mean (or median for GCaMP studies) of each independent experiment. Central bands of the violin plot (D) represent median and quartiles. The top and the bottom of the box plot (H) represent the 75th and 25th percentiles, respectively, and the line represents the median. The whiskers represent the highest and lowest values that are not outliers. Unpaired t-test (B), **Kruskal–Wallis test (D and H), or Multiple t-test comparisons corrected by Bonferroni-Dunn method (E)**. *P*-values shown as ns *P* > 0.05; *P < 0.05; **P < 0.01; ****P < 0.0001.

These errors do not affect the conclusions of the original paper.

All authors agree with this corrigendum.

## Supplementary information

Figure 1C source data

Figure 1D source data

Figure 3B source data

Figure 4E source data

