# Supplementary material for: Microglia‐synapse engulfment via PtdSer‐TREM2 ameliorates neuronal hyperactivity in Alzheimer's disease models
Source: EMBO J. 2023 Aug 14;42(19):e113246. doi: 10.15252/embj.2022113246 (PMC10548173; doi:10.15252/embj.2022113246)
Supplement: Supplementary file 1 — Appendix S1 [file EMBJ-42-e113246-s006.pdf]

Appendix Table of Contents

Appendix Figure S1……………………………………………………………………………2

Appendix Figure S2……………………………………………………………………………3

Appendix Figure S3……………………………………………………………………………4

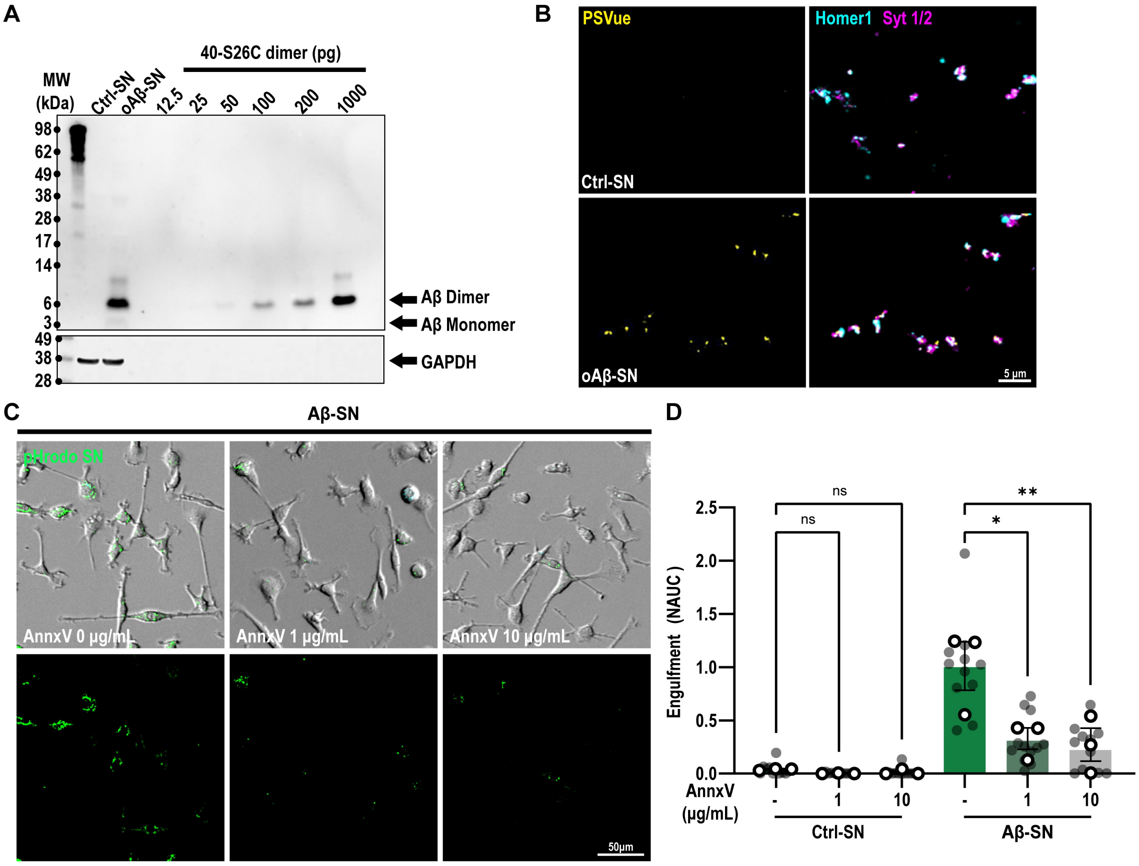

Appendix Figure S1. Aβ oligomers induce ePtdSer on fresh mouse synaptosomes which is necessary for microglial engulfment.

A. Western blot validating levels of Aβ on freshly isolated synaptosomes treated with 50nM synthetic humanized Aβ oligomers 40-S26C compared to standard curve and GAPDH loading control. Aβ oligomers were mostly dimers (6.5 kDa) with some monomers (3 kDa) in Aβ-synaptosomes (Aβ-SN) with no immunoreactivity in control synaptosomes (Ctrl-SN).
B. Immunocytochemistry showing increased levels of ePtdSer using PSVue 643 labelling (yellow) on oAβ-SN versus control Ctrl-SN immunostained with pre- and post-synaptic Synaptotagamin 1/2 (magenta) and Homer1 (cyan) respectively. Scale bar 5 µm.
C-D. Microglia simultaneously treated with oAβ-synaptosomes (oAβ-SN) (green) conjugated to pHrodo red and control synaptosomes (Ctrl-SN) conjugated to pHrodo deep red pre-treated with either 0, 1 or 10 µg/mL of Annexin-V (AnnxV). Scale bar 50 µm.
D. pHrodo fluorescence intensity with time shown as area under curve (AUC) at 3 h. AnnxV treatment significantly decreases AUC of oAβ-SN but not Ctrl-SN. Data shown as normalized to non-treated oAβ-SN (NAUC). ~40 microglia per ROI, 2 ROIs per well, 2-3 wells per experiment, n=3 independent experiments.

Data information: Data shown as mean ± SEM. Each shaded point represents 1 ROI, and each open point represents the average per experimental replicate. Two-way ANOVA followed by Bonferroni post-hoc test. P-values shown as ns $P>0.05$; *$P<0.05$; **$P<0.01$.

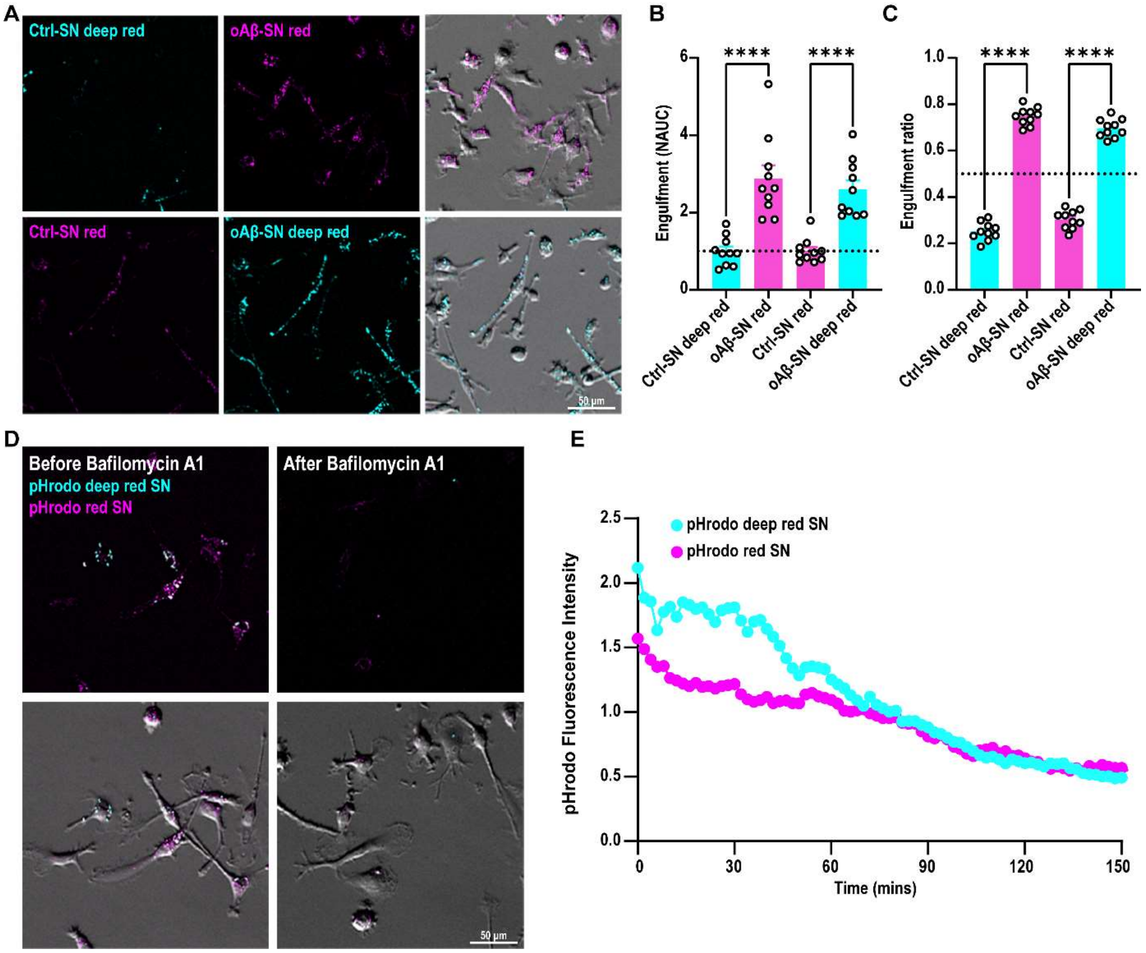

Appendix Figure S2. Microglia preferentially engulf Aβ oligomer^{+}-synaptosomes regardless of pHrodo dye.

A. Primary microglia (P0-P4) treated with simultaneously added oAβ-synaptosomes (oAβ-SN) and control synaptosomes (Ctrl-SN). Top panel: Ctrl-SN (cyan) and oAβ-SN (magenta) conjugated to deep red and red pHrodo respectively. Bottom panel: Ctrl-SN (magenta) and oAβ-SN (cyan) conjugated to red and deep red pHrodo respectively. See movie EV1. Scale bar 20 µm

B. pHrodo fluorescence intensity with time shown as area under curve (AUC) at 3 h normalized to respective control (NAUC). AUC of oAβ-SN is higher than Ctrl-SN irrespective of pHrodo paradigm. 5 ROIs per experiment, from 2 experimental replicates per paradigm.

C. Engulfment ratio (oAβ-SN or Ctrl-SN/ total sum pHrodo fluorescence) at a single time point**.** 5 ROIs per experiment, from 2 experimental replicates per paradigm.

D. Primary microglia treated simultaneously with synaptosomes conjugated to pHrodo red (red pHrodo SN, magenta) and deep red (deep red pHrodo SN, cyan) before and after 2 h treatment with bafilomycin A1 at the end of a microglia-synaptosome engulfment experiment. Scale bar 20 µm

E. Bafilomycin, which prevents acidification of lysosomes, decreases pHrodo fluorescence intensity of both pHrodo red and deep red with time (2 min intervals, bafilomycin added at t=0).

Data information: Data shown as mean ± SEM. Each point represents 1 ROI. Two-way ANOVA followed by Bonferroni post-hoc test. P-values shown as ****P<0.001.

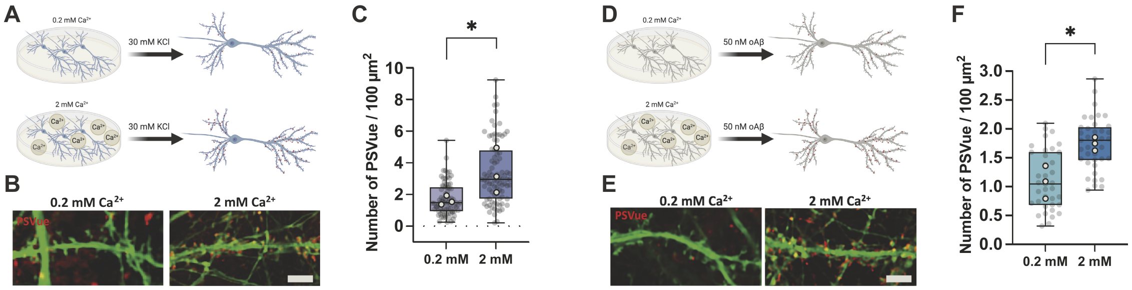

Appendix Figure S3. Aβ oligomer induced-PtdSer externalization is dependent on depolarization and calcium influx.

A. Schematic figures of depolarization experiments at 0.2 or 2 mM calcium concentration.
B. Representative images of externalized PtdSer labelled with PSVue (red) in dendritic spines after depolarization with 30 mM KCl at each calcium concentration. Scale bar, 5 µm.
C. Number of PSVue puncta per 100 μm^2 at each condition. ~20-40 ROIs per experiment, from 3 independent experiments.
D. Schematic figures of experiments treated with Aβ oligomer at 0.2 or 2 mM calcium concentration.
E. Representative images of externalized PtdSer after 50 nM Aβ oligomer treatment at each calcium concentration. Scale bar, 5 µm.
F. Number of PSVue puncta per 100 μm^2 at each condition. ~10-20 ROIs per experiment, from 3 independent experiments.

Data information: Data shown as box plots (**C** and **F**). The top and the bottom of the boxes represent the 75th and 25th percentiles, respectively, and the lines in the middle represent the median. The whiskers represent the highest and lowest values that are not outliers. Each shaded point represents 1 ROI and each open point represents the mean for each independent experiment. Unpaired *t*-test P-values shown as *P<0.05.
