# Supplementary material for: Microglia‐synapse engulfment via PtdSer‐TREM2 ameliorates neuronal hyperactivity in Alzheimer's disease models
Source: EMBO J. 2023 Aug 14;42(19):e113246. doi: 10.15252/embj.2022113246 (PMC10548173; doi:10.15252/embj.2022113246)
Supplement: Supplementary file 2 — Expanded View Figures PDF [file EMBJ-42-e113246-s004.pdf]

# Expanded View Figures

**Figure EV1. Annexin-V pretreatment of AD synaptosomes decreases microglial engulfment.**

A Crude synaptosomes were prepared using a protocol that yields electrically functional synaptosomes for several hours post-isolation, which can be depolarized and stimulated with KCl and NMDA, respectively. Electron microscopy of crude synaptosome preparation showing intact pre- (**) and postsynaptic sites (*). Scale bar 500 nm.

B Western blot showing enrichment of synaptic markers PSD-95 (green, 95 kDa) in synaptosomes (SN) compared with total homogenate (THF) fractions with respect to GAPDH (red, 37 kDa) loading control. One lane represents one mouse.

C Primary microglia grown with TGFβ express high mRNA levels of microglial genes including *Cx3cr1*, *Itgam*, *Trem2* but also homeostatic *Tmem119* with little contamination from neurons (*Map2*), astrocytes (*Gfap*), and oligodendrocytes (*Mag*). Gene expression normalized to the geomean of three housekeeping genes (*Actb*, *Gapdh*, and *Rpl32*).

D Microglia treated with NDC (NDC SN), AD (AD SN), or AD synaptosomes pretreated with 10 μg/ml Annexin-V (AD SN + AnnxV) conjugated to pHrodo red (magenta). Scale bar 50 μm.

E All isolated human synaptosomes (SN) used in this study have been tested for the presence of Aβ oligomers by western blotting. Exemplar western blot showing higher levels of Aβ trimer (14 kDa), dimer (6.5 kDa), and monomer (3 kDa) in synaptosomes prepared from the frontal cortex of AD (AD SN) patients and NDC (NDC SN) with respect to GAPDH loading control (38 kDa) loading control. One lane represents one patient.

Data information: Data shown as mean ± SEM. Each point represents the average per experimental replicate.

© 2023 The Authors

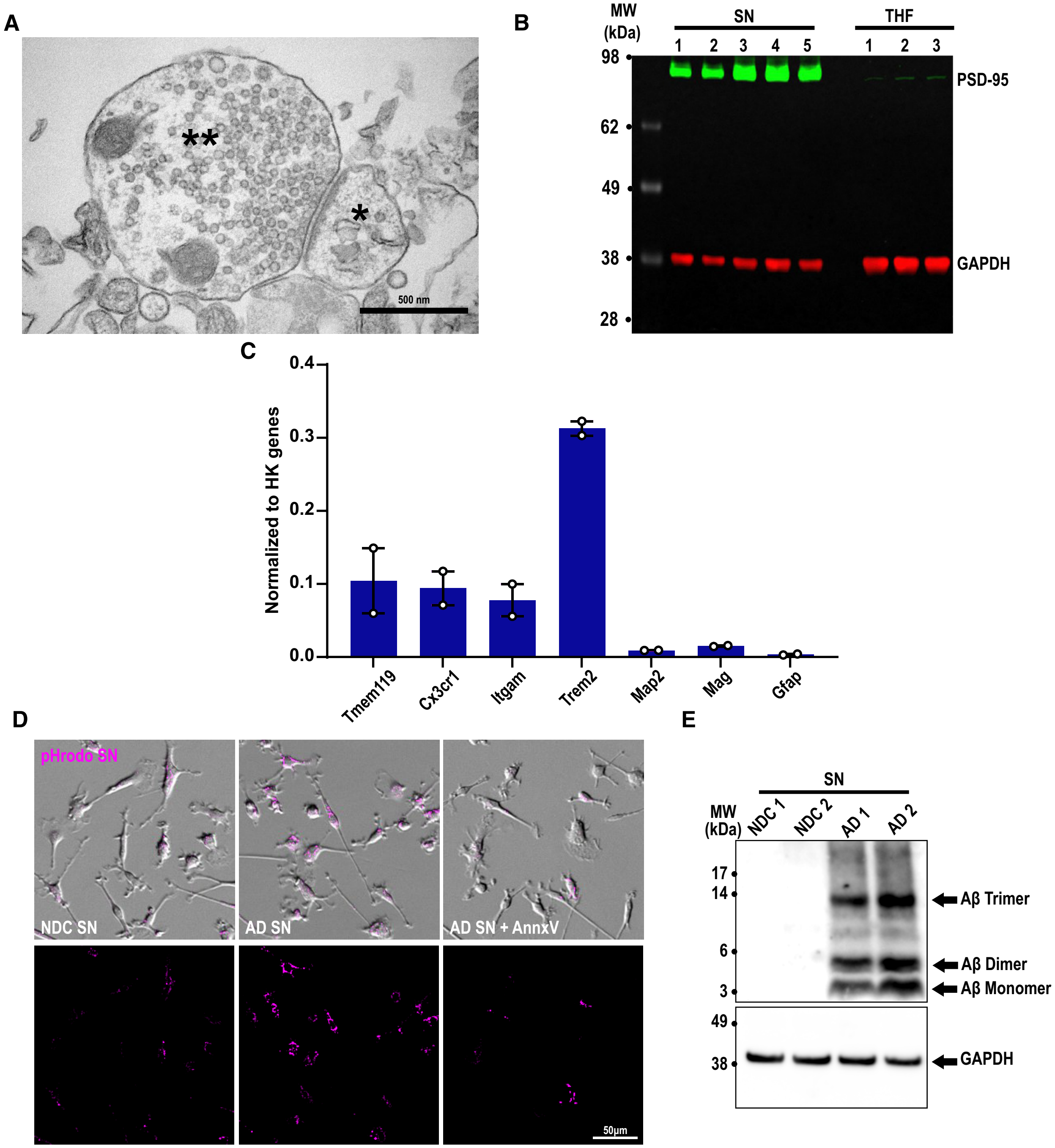


**Figure EV1.**

 © 2023 The Authors

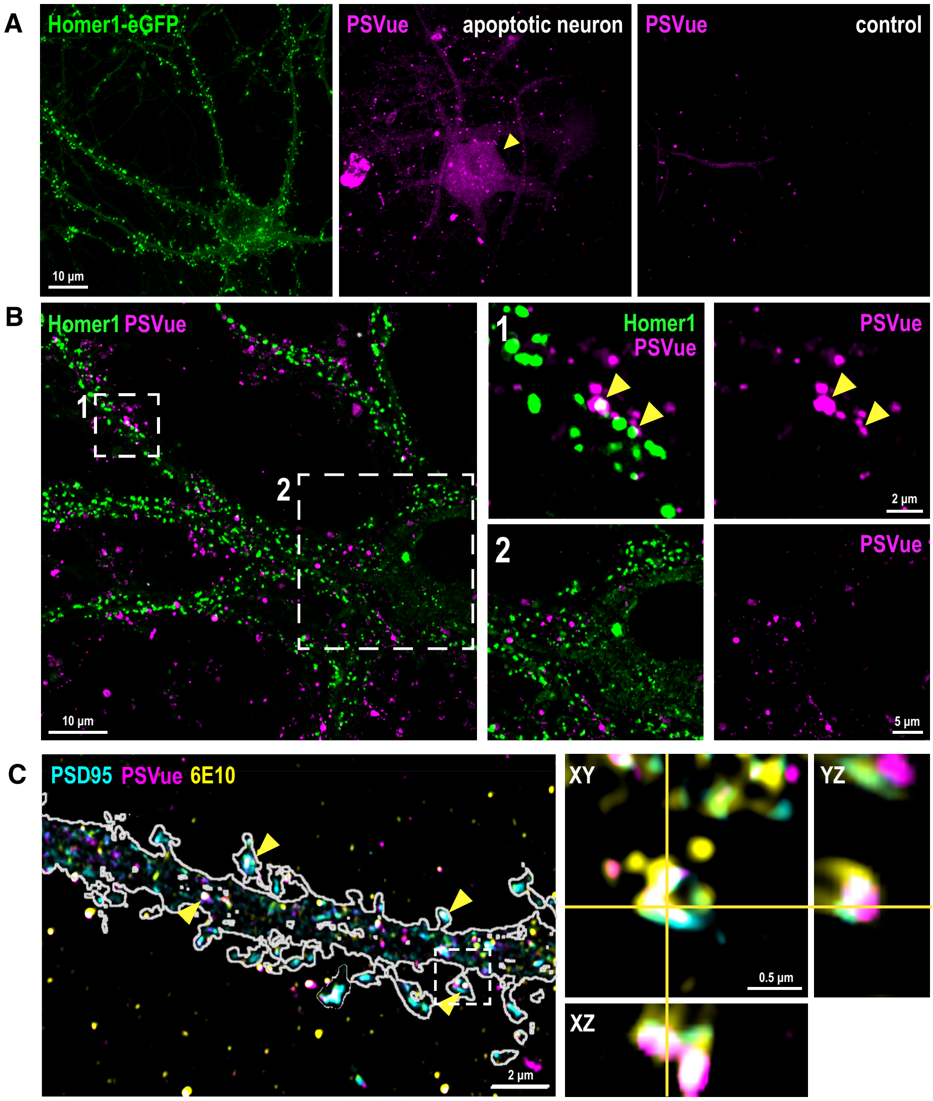


**Figure EV2. Aβ oligomers induce focal PtdSer externalization in dendritic spines.**

A Representative image of Homer1-eGFP neuron used in our studies here (green, left panel). In contrast to an apoptotic neuron (magenta, middle panel), where PSVue signals are observed in whole parts of the dying cell, the neurons used in our studies are nonapoptotic with negligent PSVue signal in the soma (right panel). Scale bar 10 µm.

B Super-resolution Airyscan images of neuronal PSvue (magenta) staining after 1-h treatment of 50 nM Aβ oligomer. Note PSVue signals in insets 1 and 2 are not random but are colocalized with or in close vicinity to Homer1-eGFP signal as indicated by yellow arrowheads. Scale bar 10, 2, and 5 µm.

C Super-resolution Airyscan images of PSD95 (cyan), PSVue (magenta) and 6E10 anti-Aβ antibody (yellow) after 1-h treatment with Aβ oligomer. Yellow arrowheads indicate the spines showing colocalized signal of PSD95, PSVue, and 6E10. Magnified orthogonal view of a single spine with PSD95, PSVue, and 6E10 colocalization in the inset. Scale bar 2 and 0.5 µm.

© 2023 The Authors

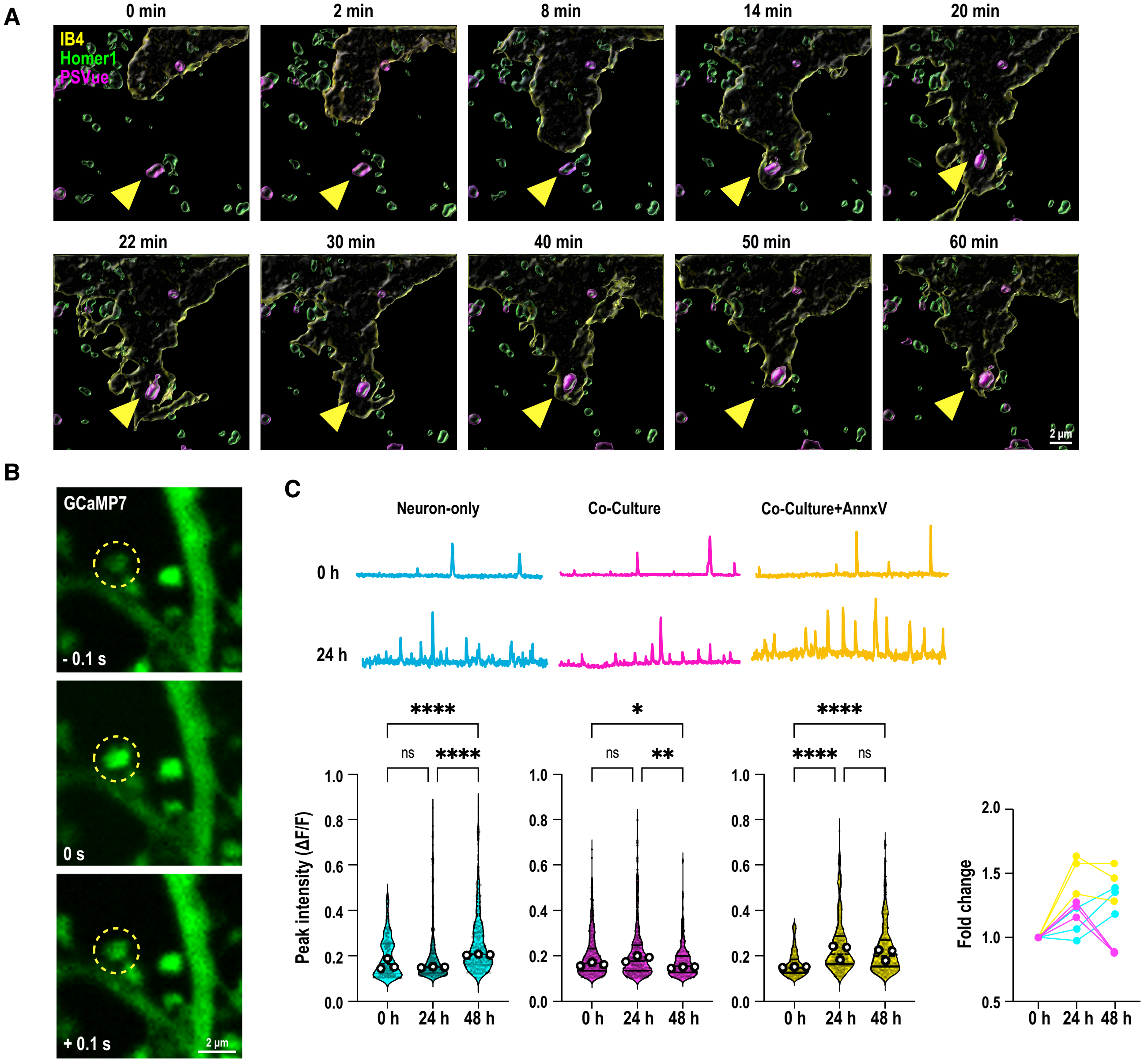


**Figure EV3. Primary microglia contact and remove ePtdSer[+] dendritic spines to resolve neuronal hyperactivity upon acute Aβ oligomer challenge.**

A Time-lapse sequence images of 10 consecutive optical sections (Δz = 0.4 μm) showing primary Homer1-eGFP neurons (green), PSVue (magenta) and microglia (yellow, labeled with Isolectin B4-647, IB4-647). Yellow arrowheads indicate PSVue[+] Homer1-eGFP dendritic spines contacted by microglia over 1 h. See Movie EV2. Scale bar 2 μm.

B Representative image of transfected GCaMP7 signal on dendritic spines at 48-h post-oAβ challenge. Scale bar 2 μm.

C (Top panel) Representative traces of GCaMP7 signal in dendritic spines at 0-h and 24-h post-treatment with Aβ oligomer from neuron-only culture (cyan), neuron–microglia co-culture (magenta), and neuron–microglia co-culture treated with Annexin-V (AnnxV, yellow). (Bottom panel) Violin plots showing normalized peak intensities ($\Delta F/F_0$) of spontaneous GCaMP7 signal in dendritic spines before (0 h), at 24-h and 48-h post-treatment with Aβ oligomer. (Bottom right) Relative fold change. ~10 spines per neuron, ~10 neurons per experiment, from three independent experiments.

Data information: Data shown as violin plot, central bands represent median and quartiles. Each shaded point represents one ROI, and each open point represents the median of each independent experiment. Kruskal–Wallis test followed by Dunn's multiple comparisons test. *P*-values shown as ns $P > 0.05$; *$P < 0.05$; **$P < 0.01$; ****$P < 0.0001$.

 © 2023 The Authors

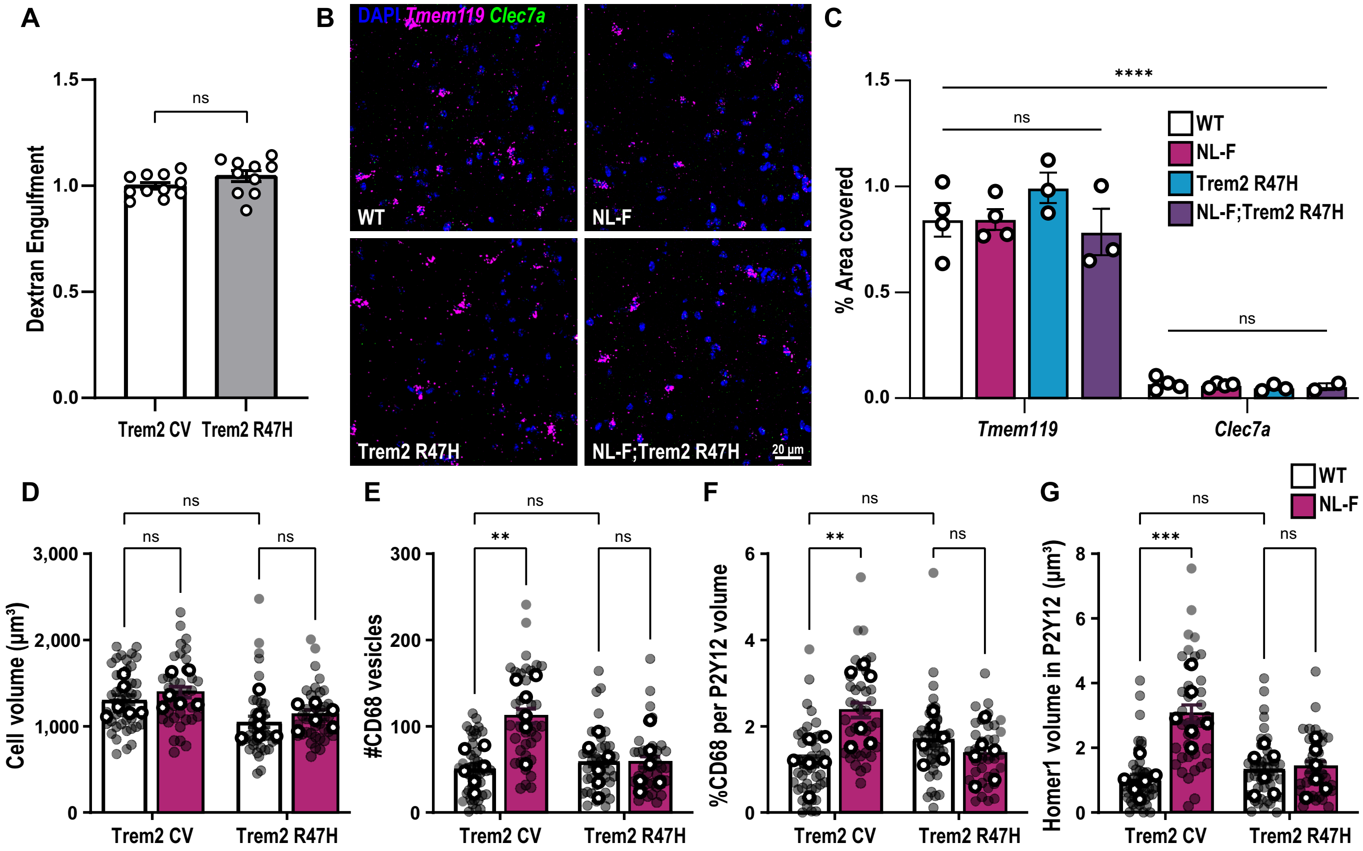


**Figure EV4. Microglia require functional TREM2 to engulf synapses *in vivo*.**

A Primary microglia (P0-P4) prepared from either Trem2 CV or Trem2 R47H KI mice treated with dextran conjugated to pHrodo red to compare engulfment of inert particles, showing no difference between the two genotypes.

B Representative images from hippocampal CA1 stratum lacunosum-moleculare from 6-month-old WT, Trem2 R47H KI, NL-F KI, and NL-F KI; Trem2 R47H KI mice probing for *Tmem119* (magenta) and *Clec7a* (green) by *in situ* hybridization (RNAScope). DAPI shown in blue. Scale bar, 20 μm.

C Quantification of the percent of area covered either by *Tmem119* or *Clec7a* spots showing higher levels of *Tmem119* compared with *Clec7a* across all genotypes with no difference between genotypes. $n$ = 2–4 animals.

D–G Hippocampal CA1 stratum radiatum sections from 6-month-old WT, Trem2 R47H KI, NL-F KI, and NL-F KI; Trem2 R47H KI mice immunostained for P2Y12 (red), CD68 (magenta), and Homer1 (green). 3D surface rendering reconstructions of microglia showing increased Homer1 signal inside microglia in NL-F KI but not NL-F KI; Trem2 R47H KI compared with WT and Trem2 R47H KI, respectively. Quantification of microglial P2Y12$^+$ cell volume (D), number of CD68$^+$ lysosomal vesicles per microglia (E), percentage of P2Y12$^+$ volume occupied by CD68$^+$ immunoreactive vesicles (F), and total volume of Homer1$^+$ material within P2Y12$^+$ microglia (G). All volumes are represented in $\mu m^3$. Six to nine microglia per animal, $n$ = 6 animals per genotype.

Data information: Data shown as mean ± SEM. Each shaded point represents one ROI, and each open point represents the average per experimental replicate. Unpaired $t$-test (A) or two-way ANOVA followed by Bonferroni's *post hoc* test. $P$-values shown as ns $P > 0.05$; $^{**}P < 0.01$; $^{***}P < 0.001$; $^{****}P < 0.0001$.

© 2023 The Authors

**Figure EV5. Trem2 loss-of-function exacerbates synaptic ePtdSer in the NL-F model of amyloidosis.**

A Super-resolution images from the hippocampal CA1 stratum radiatum of 6-month-old NL-F KI and NL-F KI; Trem2 R47H KI mice immunostained for presynaptic Synaptotagmin 1/2 (Syt1/2, red) and postsynaptic Homer1 (green). PSVue 643 (magenta) is ICV injected. Upper panels show Syt1/2, Homer1, and PSVue. Lower panels show Homer1 and PSVue only. Scale bar 1 μm.

B PSVue volume represented in $\mu m^3$. Three ROIs per animal, $n$ = 3–4 per genotype.

C Percentage of synaptic Synaptotagmin 1/2 puncta within 0.25 μm of PSVue showing increased percentage of $PSVue^+$ synapses in NL-F KI; Trem2 R47H KI mice compared with NL-F KI. Three ROIs per animal, $n$ = 3–4 animals per genotype.

D Percentage of PSVue volume within 0.25 μm of synaptic Synaptotagmin 1/2 puncta. Three ROIs per animal, $n$ = 3–4 animals per genotype.

E Super-resolution images from the hippocampal CA1 dentate gyrus hilus of 4-month-old WT and J20 Tg mice immunostained for pre-Synaptotagmin 1/2 (Syt1/2, red) and postsynaptic Homer1 (green). PSVue 643 (magenta) is ICV injected. Top panels show Syt1/2, Homer1, and PSVue. Bottom panels show Homer1 and PSVue only. Insets show either triple (Syt1/2, Homer1 and PSVue; top panel) or double colocalization (Homer1 and PSVue; bottom panel). Scale bar 1 μm.

F PSVue total volume per ROI (7,500 $\mu m^3$) showing increased PSVue in J20 Tg mice compared with WT. Three ROIs per animal, $n$ = 3–4 animals per genotype.

G Percentage of synaptic Homer1 puncta within 0.25 μm of PSVue showing an increase in $PSVue^+$ synapses in J20 Tg compared with WT. Three ROIs per animal, $n$ = 3–4 animals per genotype.

H Percentage of synaptic Synaptotagmin 1/2 puncta within 0.25 μm of PSVue showing an increase in $PSVue^+$ synapses in J20 Tg compared with WT. Three ROIs per animal, $n$ = 3–4 animals per genotype.

I Percentage of PSVue volume within 0.25 μm of synaptic Homer1 puncta. Three ROIs per animal, $n$ = 3–4 animals per genotype.

Data information: Data shown as mean ± SEM. Each shaded point represents one ROI, and each open point represents the average per experimental replicate. Two-way ANOVA followed by Bonferroni's *post hoc* test. $P$-values shown as ns $P > 0.05$; $^*P < 0.05$; $^{**}P < 0.01$.


© 2023 The Authors

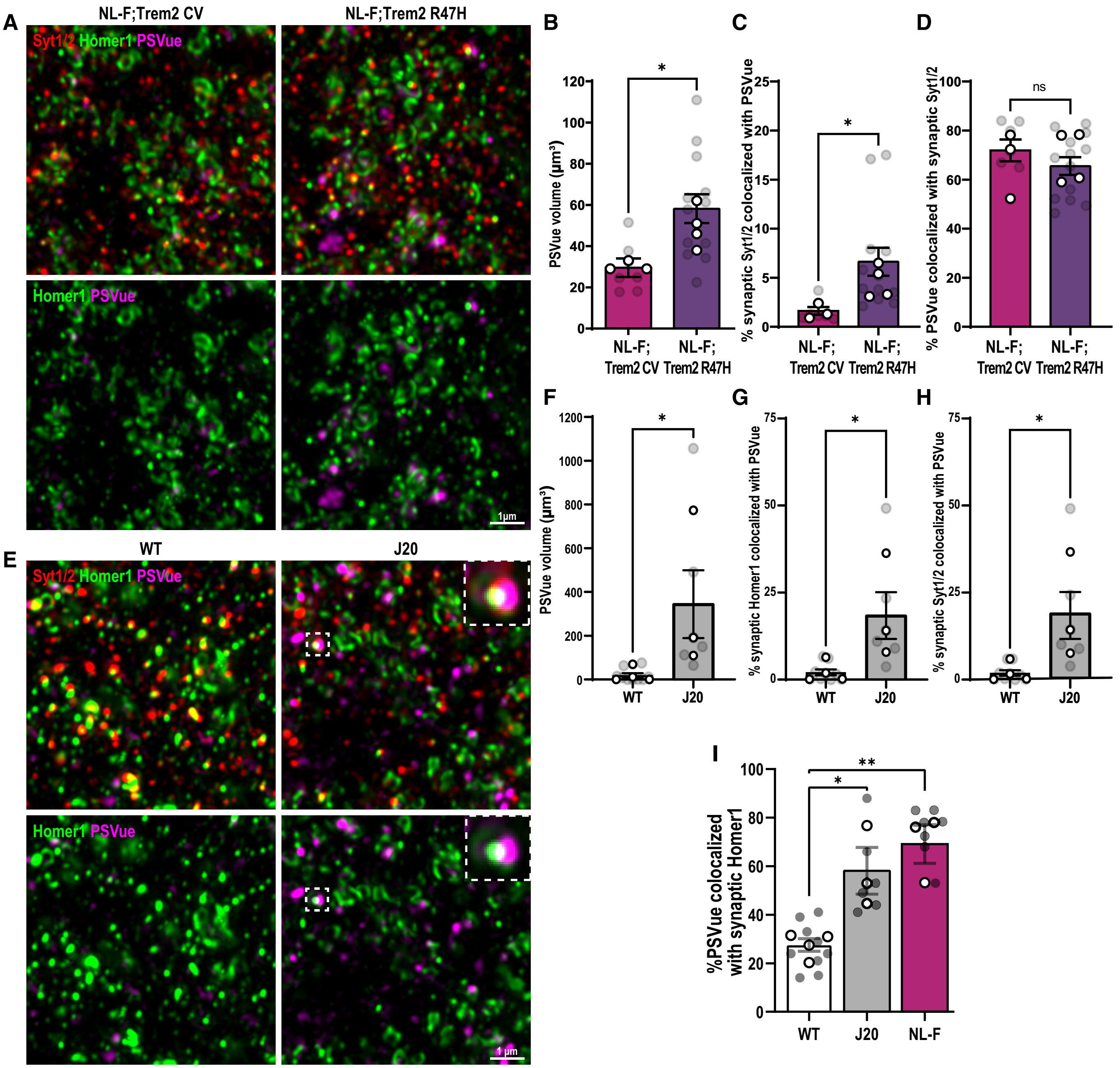

A
NL-F;Trem2 CV
NL-F;Trem2 R47H
Syt1/2 Homer1 PSVue
Homer1 PSVue
1μm
B
PSVue volume (μm³)
NL-F; Trem2 CV
NL-F; Trem2 R47H
C
% synaptic Syt1/2 colocalized with PSVue
D
% PSVue colocalized with synaptic Syt1/2
ns
E
WT
J20
F
PSVue volume (μm³)
G
% synaptic Homer1 colocalized with PSVue
H
% synaptic Syt1/2 colocalized with PSVue
I
%PSVue colocalized with synaptic Homer1
NL-F


Figure EV5.
