# Supplementary material for: Microglia‐synapse engulfment via PtdSer‐TREM2 ameliorates neuronal hyperactivity in Alzheimer's disease models
Source: EMBO J. 2023 Aug 14;42(19):e113246. doi: 10.15252/embj.2022113246 (PMC10548173; doi:10.15252/embj.2022113246)
Supplement: Supplementary file 3 — Movie EV1 [file EMBJ-42-e113246-s011.zip › Movie EV1.docx]

Movie EV1. Microglia preferentially engulf Aβ oligomer^+^-synaptosomes over control.

Time-lapse video of primary microglia preferentially engulfing Aβ-synaptosomes in pHrodo red (magenta) over control synaptosomes in pHrodo deep red (cyan) over 10 h (2-5 min intervals). Scale bar 50 μm.
