# Supplementary material for: Microglia‐synapse engulfment via PtdSer‐TREM2 ameliorates neuronal hyperactivity in Alzheimer's disease models
Source: EMBO J. 2023 Aug 14;42(19):e113246. doi: 10.15252/embj.2022113246 (PMC10548173; doi:10.15252/embj.2022113246)
Supplement: Supplementary file 4 — Movie EV2 [file EMBJ-42-e113246-s014.zip › Movie EV2.docx]

Movie EV2. Microglia contact ePtdSer^+^ dendritic spines.

Time-lapse video of microglia (yellow, labelled with IB4-647, 3D rendered) co-cultured with Homer1-eGFP hippocampal neurons (green) treated with 50 nM Aβ oligomers. Microglia contact PSVue^+^ (magenta) Homer-1eGFP dendritic spines. Scale bar 5 μm.
