# Supplementary material for: Microglia‐synapse engulfment via PtdSer‐TREM2 ameliorates neuronal hyperactivity in Alzheimer's disease models
Source: EMBO J. 2023 Aug 14;42(19):e113246. doi: 10.15252/embj.2022113246 (PMC10548173; doi:10.15252/embj.2022113246)
Supplement: Supplementary file 5 — Movie EV3 [file EMBJ-42-e113246-s008.zip › Movie EV3.docx]

Movie EV3. Microglia internalize ePtdSer^+^ dendritic spines.

Time-lapse video of microglia (blue, labelled with IB4-647, 3D rendered) co-cultured with Homer1-eGFP hippocampal neurons (green) treated with 50 nM Aβ oligomers. Microglia internalize PSVue^+^ (magenta) Homer-1eGFP dendritic spines. Scale bar 5 μm.
