# Supplementary material for: Microglia‐synapse engulfment via PtdSer‐TREM2 ameliorates neuronal hyperactivity in Alzheimer's disease models
Source: EMBO J. 2023 Aug 14;42(19):e113246. doi: 10.15252/embj.2022113246 (PMC10548173; doi:10.15252/embj.2022113246)
Supplement: Supplementary file 6 — Movie EV4 [file EMBJ-42-e113246-s009.zip › Movie EV4.docx]

Movie EV4. Spontaneous calcium transients in GCaMP7 transfected hippocampal neurons.

Normalized fluorescence of dendritic spines of neurons transfected with GCaMP7. A non-linear scale was used to show the fluorescence intensity, ranged from low (dark blue) to high values (white). Scale bar 5 μm.
