# Supplementary material for: Microglia‐synapse engulfment via PtdSer‐TREM2 ameliorates neuronal hyperactivity in Alzheimer's disease models
Source: EMBO J. 2023 Aug 14;42(19):e113246. doi: 10.15252/embj.2022113246 (PMC10548173; doi:10.15252/embj.2022113246)
Supplement: Supplementary file 7 — Movie EV5 [file EMBJ-42-e113246-s007.zip › Movie EV5.docx]

**Movie EV5. Increased ePtdSer on hyperactive spines.**

Spontaneous calcium transients in GCaMP7 (green) transfected hippocampal neurons stained with PSVue (magenta) showing that hyperactive spines are PSVue^+^ upon acute challenge with Aβ oligomers. Scale bar 5 μm.
