# Supplementary material for: Microglia‐synapse engulfment via PtdSer‐TREM2 ameliorates neuronal hyperactivity in Alzheimer's disease models
Source: EMBO J. 2023 Aug 14;42(19):e113246. doi: 10.15252/embj.2022113246 (PMC10548173; doi:10.15252/embj.2022113246)
Supplement: Supplementary file 8 — Movie EV6 [file EMBJ-42-e113246-s003.zip › Movie EV6.docx]

Movie EV6. No overt changes in motility and morphology in Trem2 R47 KI microglia.

Time-lapse video of primary microglia prepared from Trem2 R47H KI mice showing no overt changes microglial morphology and motility. Scale bar 50 μm.
