# Supplementary figures and images for: Microglia‐synapse engulfment via PtdSer‐TREM2 ameliorates neuronal hyperactivity in Alzheimer's disease models
Source: EMBO J. 2023 Aug 14;42(19):e113246. doi: 10.15252/embj.2022113246 (PMC10548173; doi:10.15252/embj.2022113246)

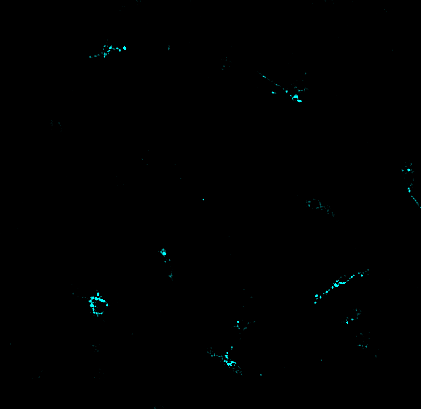

Supplement: Supplementary file 11 — Source Data for Figure 1 [file EMBJ-42-e113246-s012.zip › Figure 1/1B/Ctrl-SN.tif]

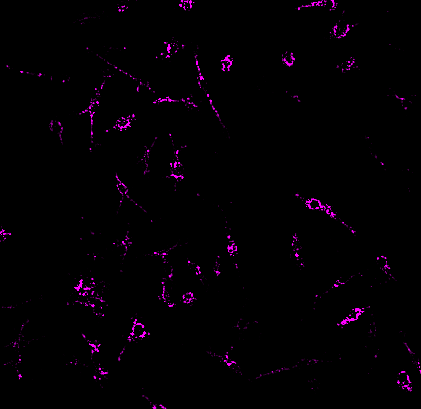

Supplement: Supplementary file 11 — Source Data for Figure 1 [file EMBJ-42-e113246-s012.zip › Figure 1/1B/oABeta-SN.tif]

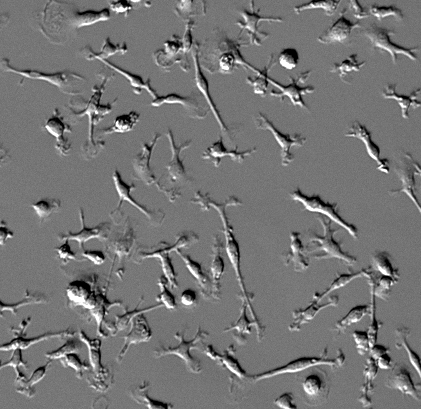

Supplement: Supplementary file 11 — Source Data for Figure 1 [file EMBJ-42-e113246-s012.zip › Figure 1/1B/Primary microglia brightfield.tif]

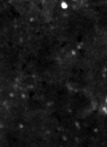

Supplement: Supplementary file 11 — Source Data for Figure 1 [file EMBJ-42-e113246-s012.zip › Figure 1/1F/control_0 min.tif]

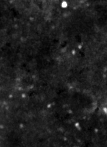

Supplement: Supplementary file 11 — Source Data for Figure 1 [file EMBJ-42-e113246-s012.zip › Figure 1/1F/control_15 min.tif]

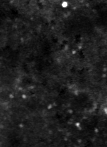

Supplement: Supplementary file 11 — Source Data for Figure 1 [file EMBJ-42-e113246-s012.zip › Figure 1/1F/control_20 min.tif]

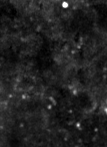

Supplement: Supplementary file 11 — Source Data for Figure 1 [file EMBJ-42-e113246-s012.zip › Figure 1/1F/control_30 min.tif]

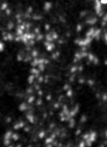

Supplement: Supplementary file 11 — Source Data for Figure 1 [file EMBJ-42-e113246-s012.zip › Figure 1/1F/oABeta treated_0min.tif]

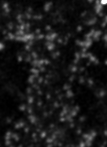

Supplement: Supplementary file 11 — Source Data for Figure 1 [file EMBJ-42-e113246-s012.zip › Figure 1/1F/oABeta treated_15min.tif]

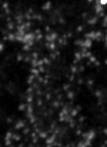

Supplement: Supplementary file 11 — Source Data for Figure 1 [file EMBJ-42-e113246-s012.zip › Figure 1/1F/oABeta treated_30min.tif]

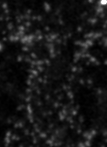

Supplement: Supplementary file 11 — Source Data for Figure 1 [file EMBJ-42-e113246-s012.zip › Figure 1/1F/oABeta treated_45min.tif]

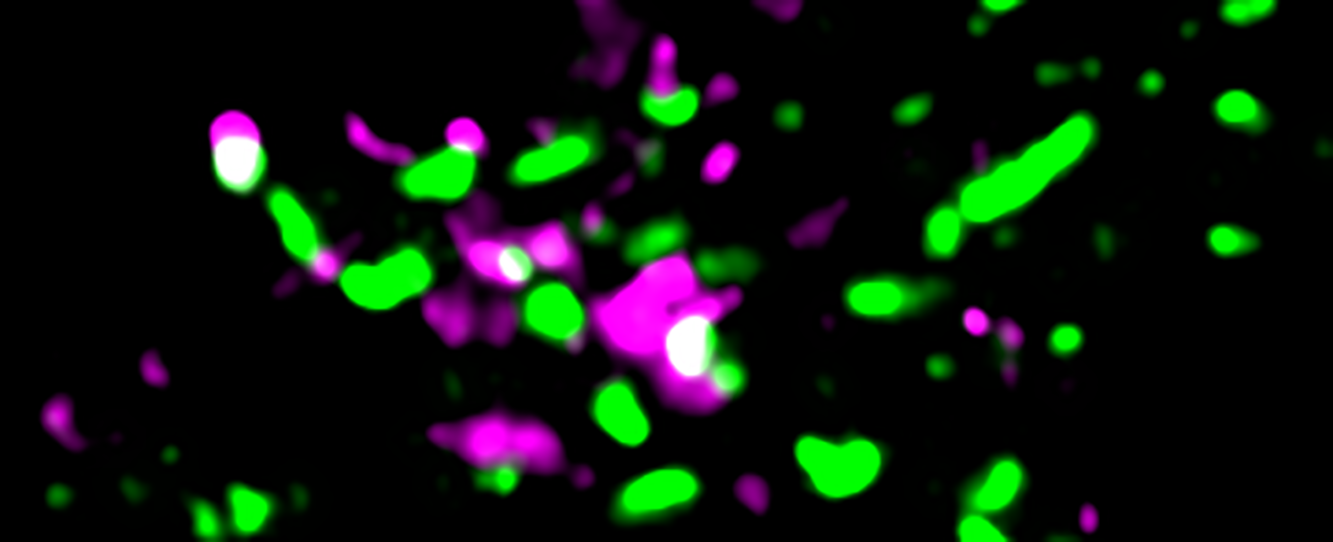

Supplement: Supplementary file 11 — Source Data for Figure 1 [file EMBJ-42-e113246-s012.zip › Figure 1/1H/1H_XY.tif]

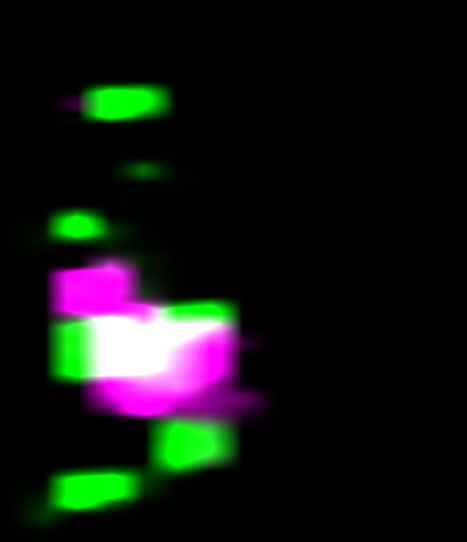

Supplement: Supplementary file 11 — Source Data for Figure 1 [file EMBJ-42-e113246-s012.zip › Figure 1/1H/1H_XZ.tif]

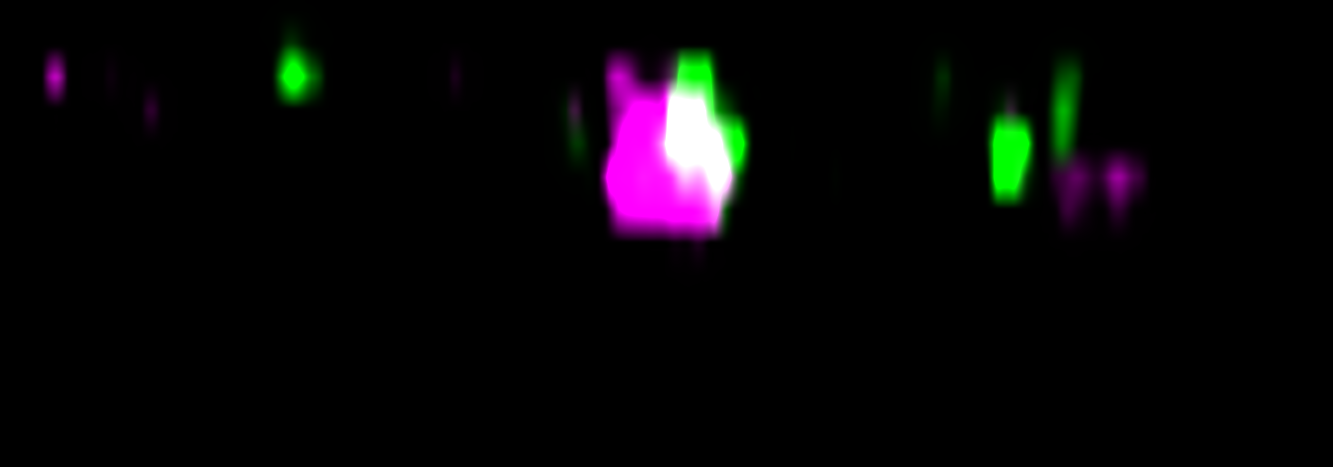

Supplement: Supplementary file 11 — Source Data for Figure 1 [file EMBJ-42-e113246-s012.zip › Figure 1/1H/H_XZ.tif]

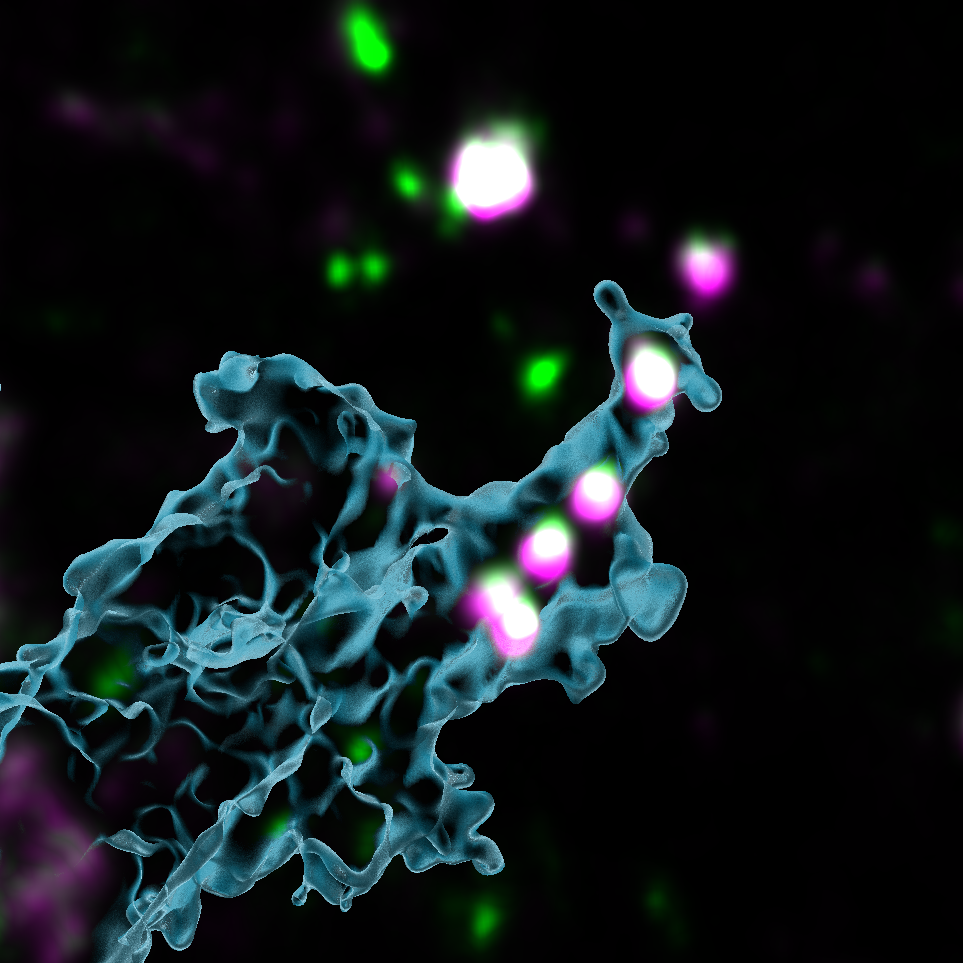

Supplement: Supplementary file 12 — Source Data for Figure 2 [file EMBJ-42-e113246-s001.zip › Figure 2/2A/Fig2_A-0 min.tif]

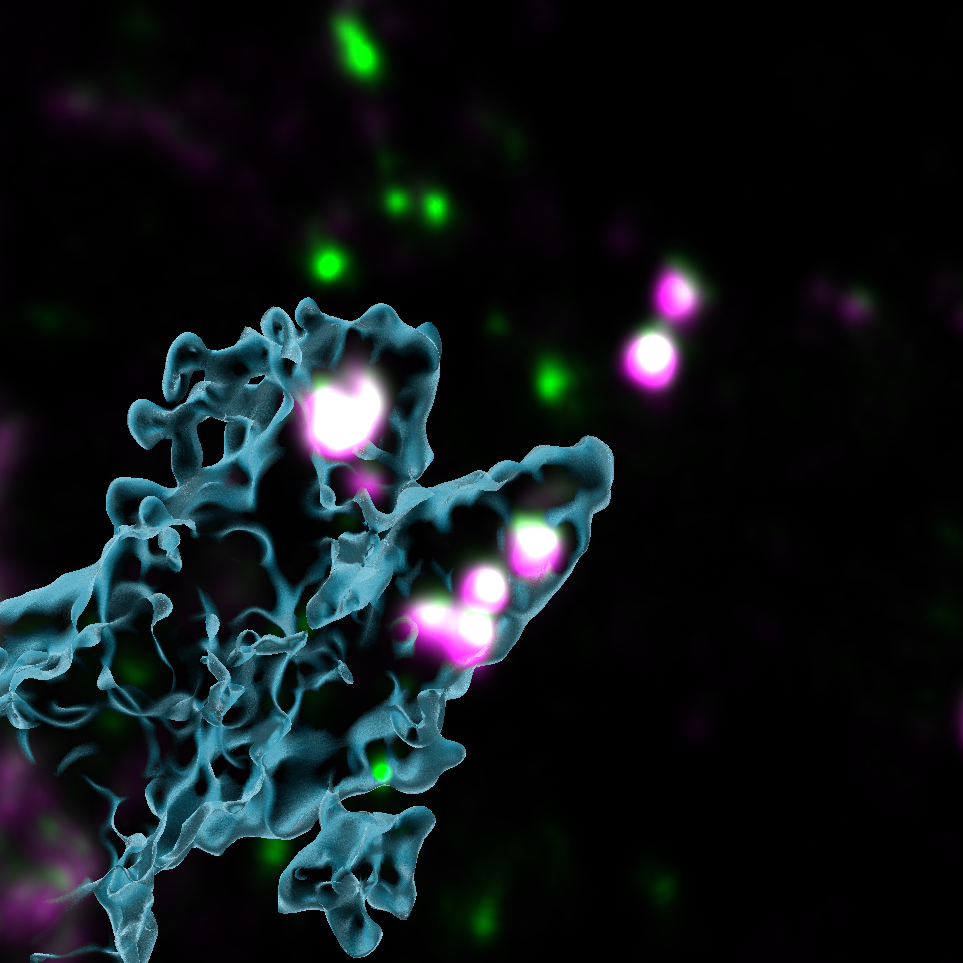

Supplement: Supplementary file 12 — Source Data for Figure 2 [file EMBJ-42-e113246-s001.zip › Figure 2/2A/Fig2_A-10 min.tif]

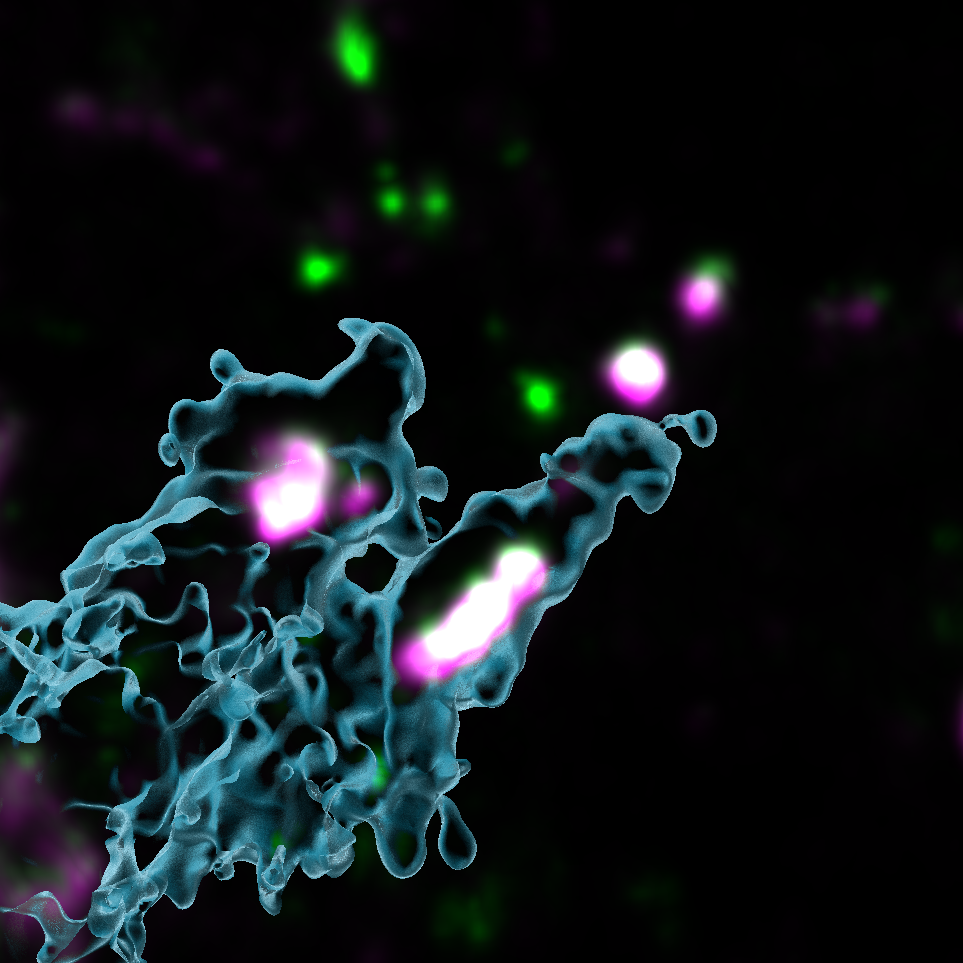

Supplement: Supplementary file 12 — Source Data for Figure 2 [file EMBJ-42-e113246-s001.zip › Figure 2/2A/Fig2_A-14 min.tif]

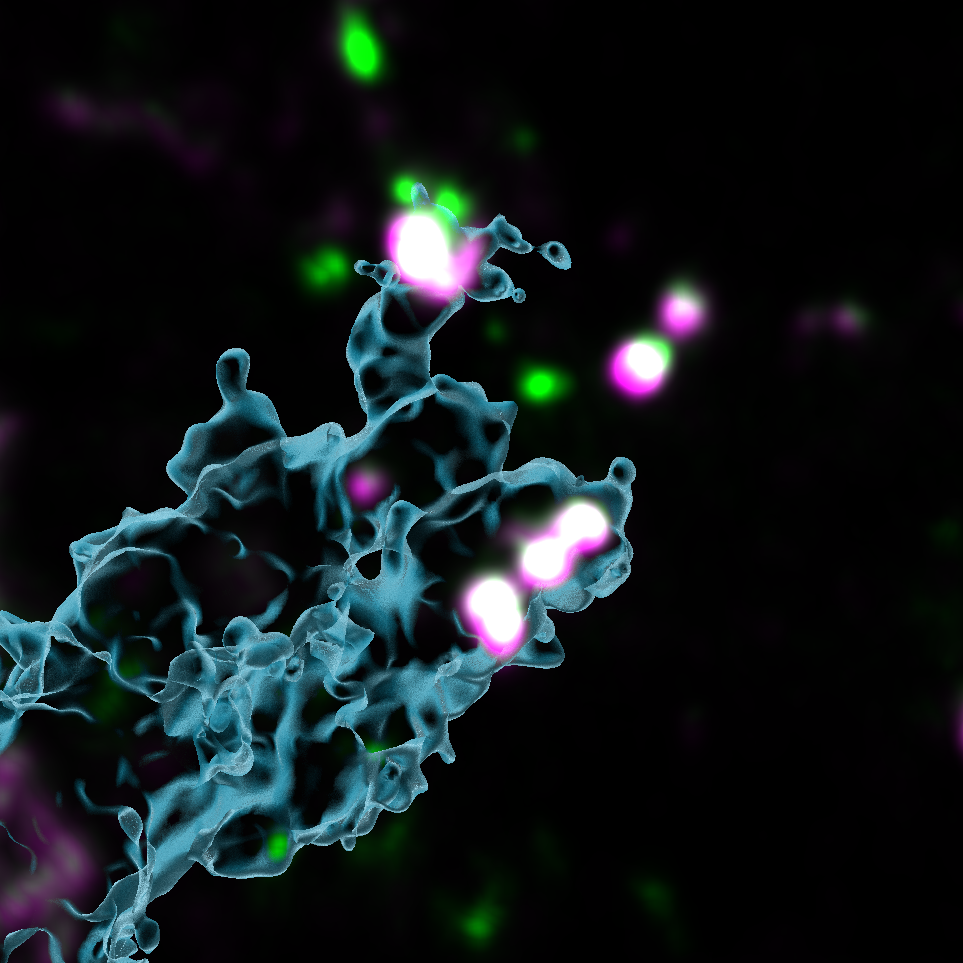

Supplement: Supplementary file 12 — Source Data for Figure 2 [file EMBJ-42-e113246-s001.zip › Figure 2/2A/Fig2_A-6 min.tif]

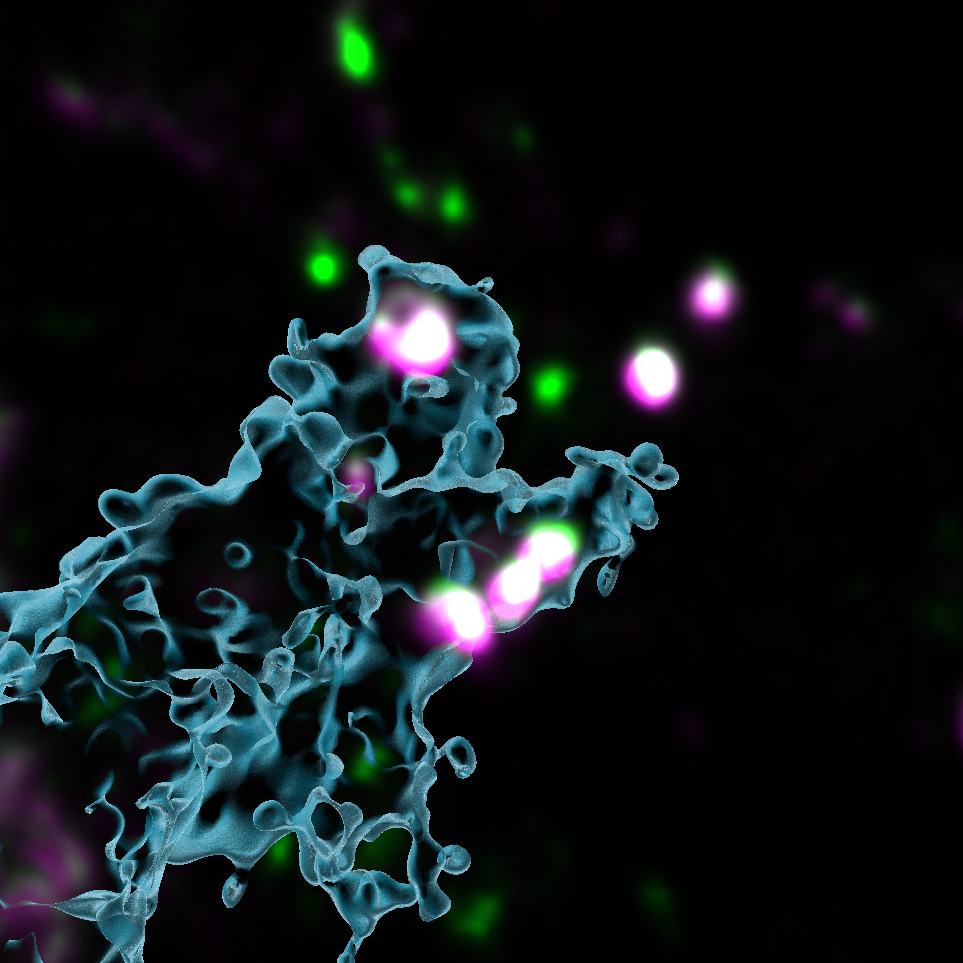

Supplement: Supplementary file 12 — Source Data for Figure 2 [file EMBJ-42-e113246-s001.zip › Figure 2/2A/Fig2_A-8 min.tif]

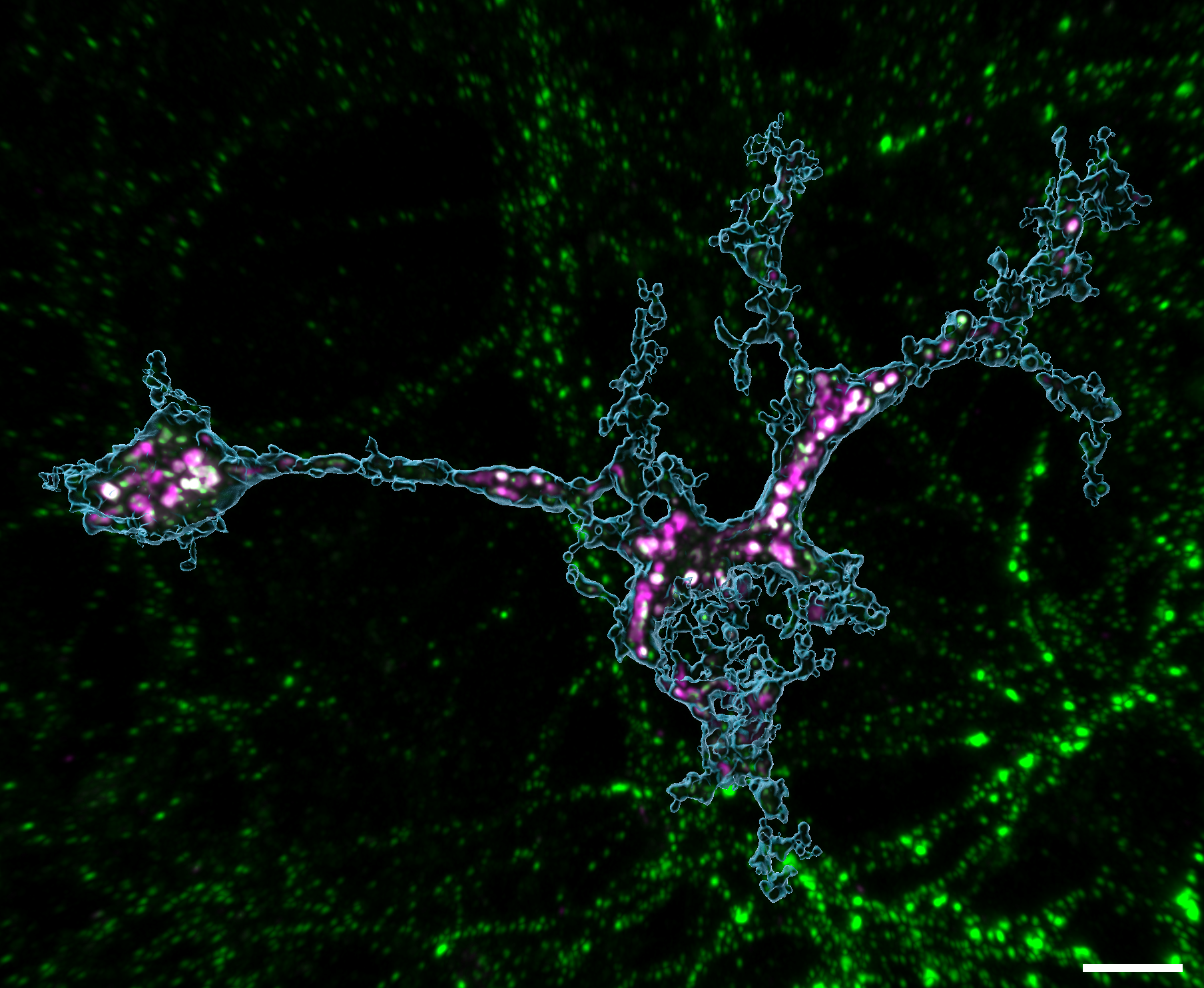

Supplement: Supplementary file 12 — Source Data for Figure 2 [file EMBJ-42-e113246-s001.zip › Figure 2/2C/Figure 2C.tif]

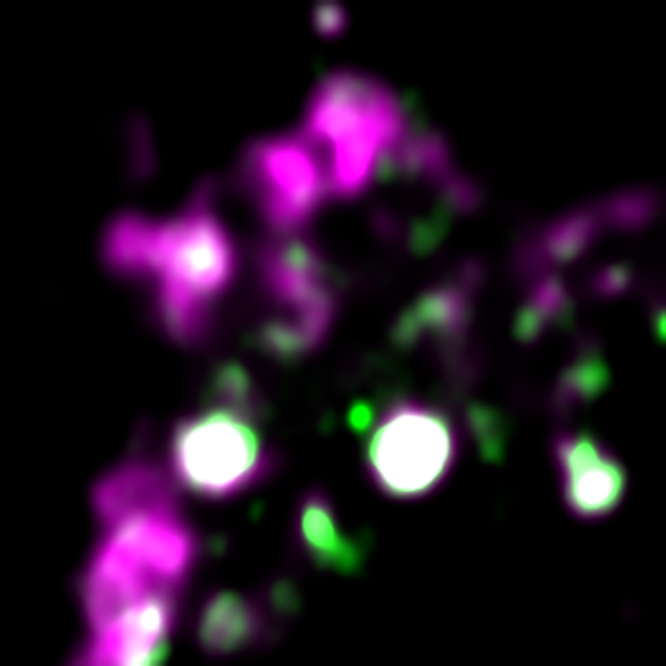

Supplement: Supplementary file 12 — Source Data for Figure 2 [file EMBJ-42-e113246-s001.zip › Figure 2/2C/Figure 2C_XY.tif]

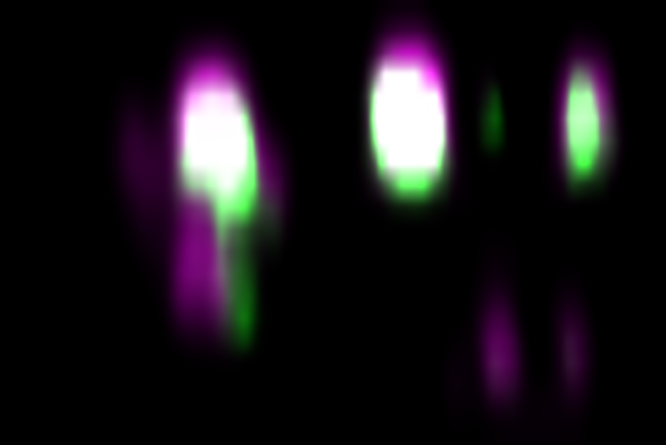

Supplement: Supplementary file 12 — Source Data for Figure 2 [file EMBJ-42-e113246-s001.zip › Figure 2/2C/Figure 2C_XZ.tif]

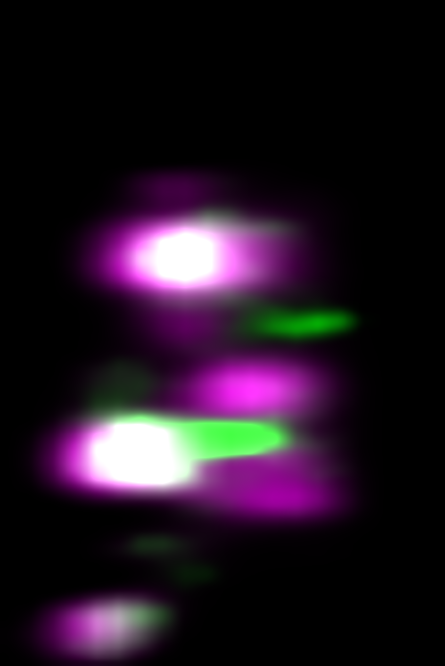

Supplement: Supplementary file 12 — Source Data for Figure 2 [file EMBJ-42-e113246-s001.zip › Figure 2/2C/Figure 2C_YZ.tif]

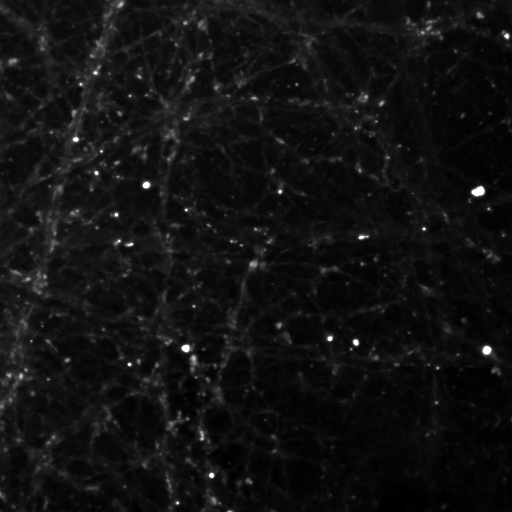

Supplement: Supplementary file 12 — Source Data for Figure 2 [file EMBJ-42-e113246-s001.zip › Figure 2/2D/Figure 2_Raw.tif]

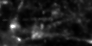

Supplement: Supplementary file 12 — Source Data for Figure 2 [file EMBJ-42-e113246-s001.zip › Figure 2/2D/Figure 2D.tif]

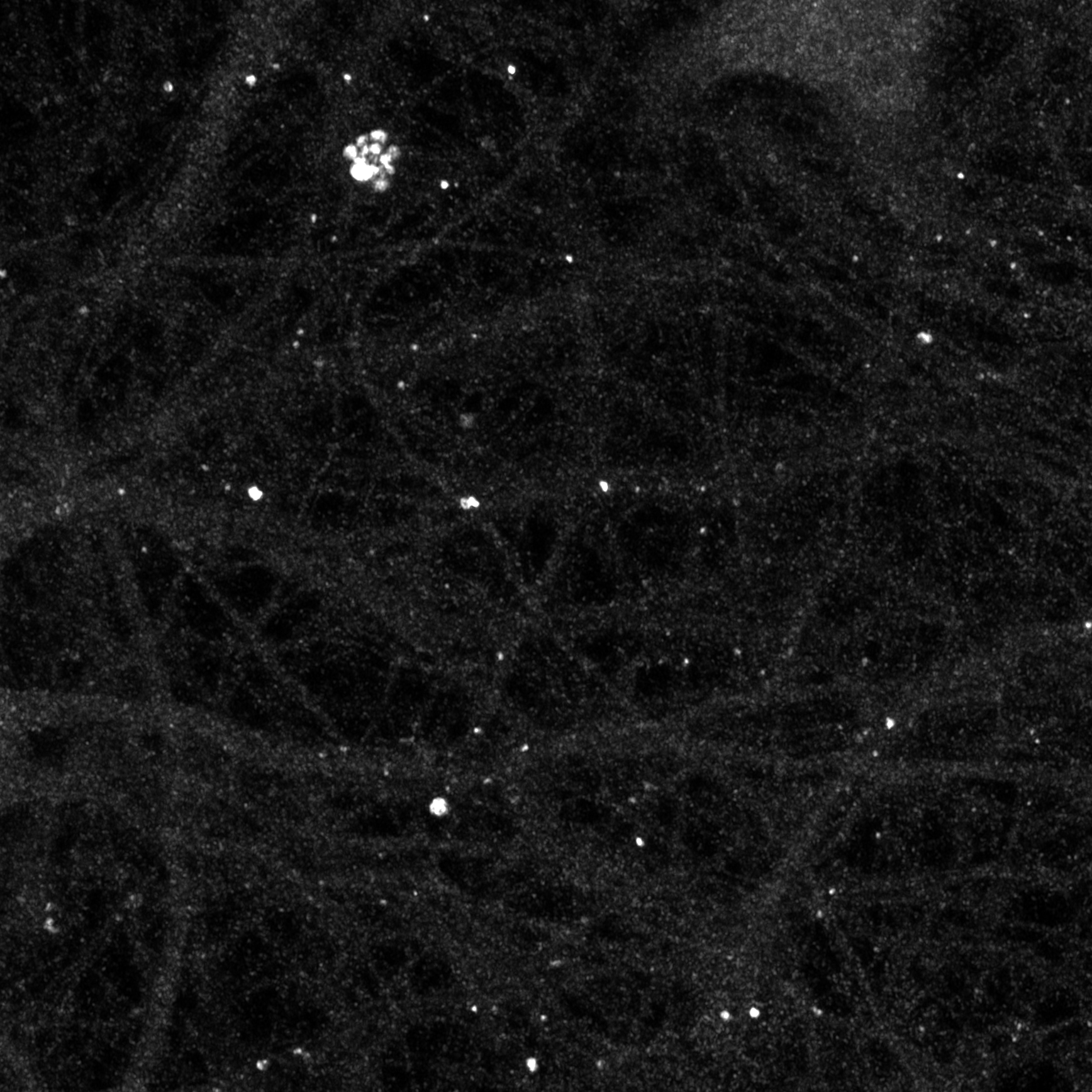

Supplement: Supplementary file 12 — Source Data for Figure 2 [file EMBJ-42-e113246-s001.zip › Figure 2/2I/Co-Culture.tif]

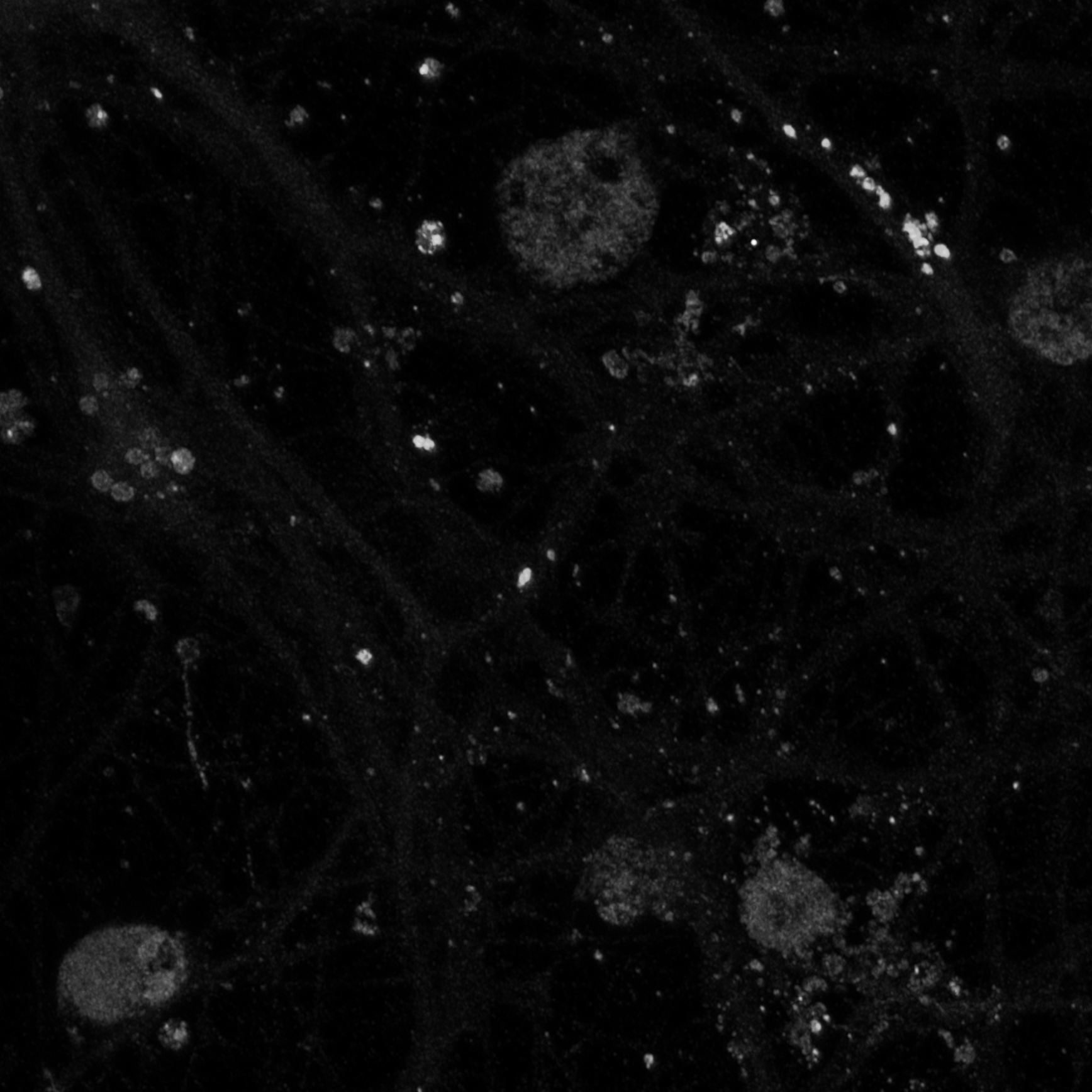

Supplement: Supplementary file 12 — Source Data for Figure 2 [file EMBJ-42-e113246-s001.zip › Figure 2/2I/Co-Culture+AnnV.tif]

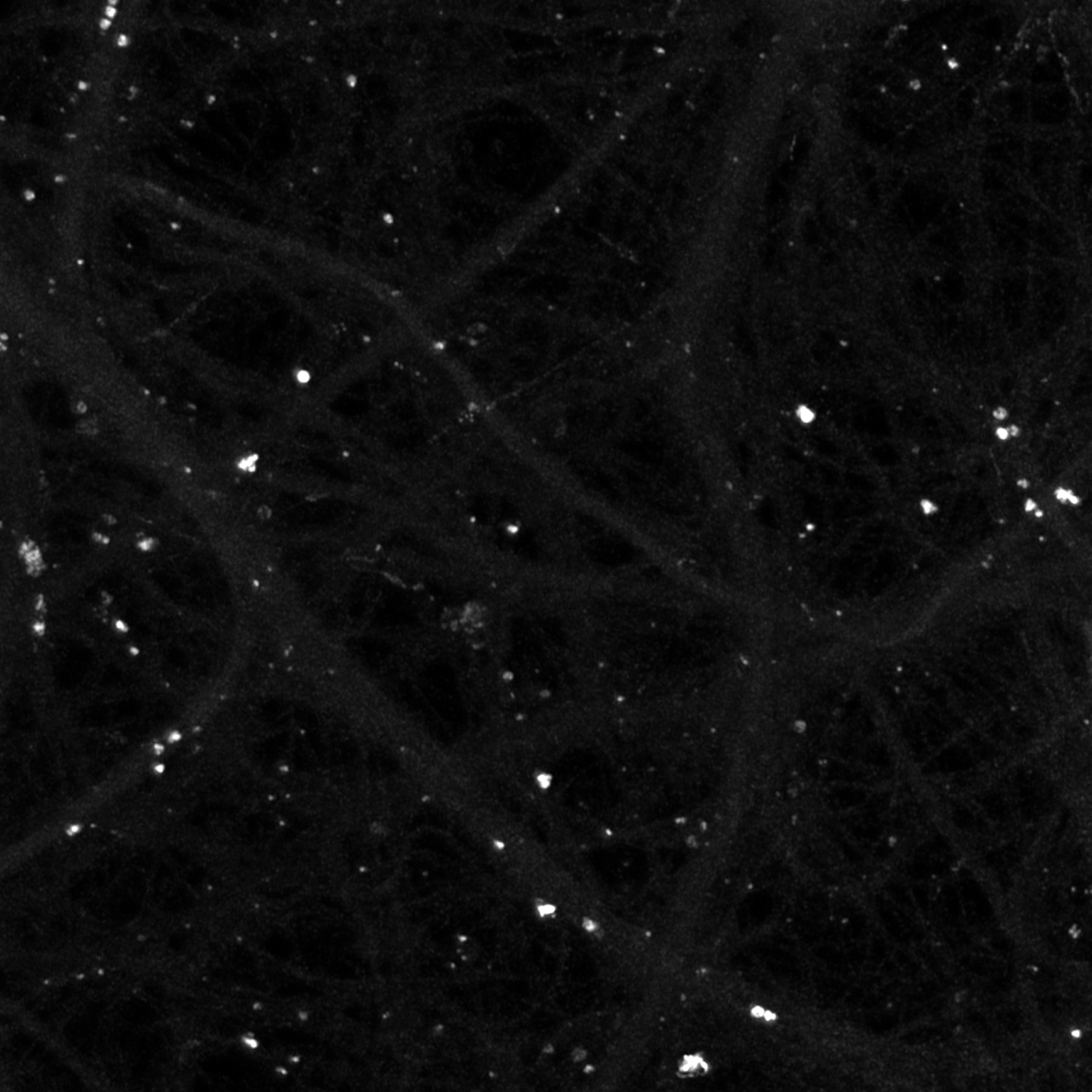

Supplement: Supplementary file 12 — Source Data for Figure 2 [file EMBJ-42-e113246-s001.zip › Figure 2/2I/Neuron-only.tif]

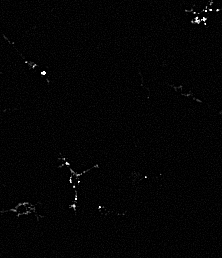

Supplement: Supplementary file 13 — Source Data for Figure 3 [file EMBJ-42-e113246-s015.zip › Figure 3/3A/Trem2 CV Ctrl-SN.tif]

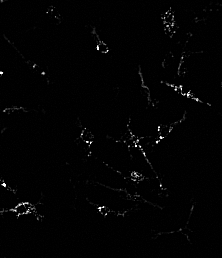

Supplement: Supplementary file 13 — Source Data for Figure 3 [file EMBJ-42-e113246-s015.zip › Figure 3/3A/Trem2 CV oABeta-SN.tif]

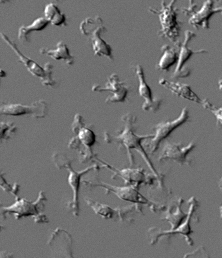

Supplement: Supplementary file 13 — Source Data for Figure 3 [file EMBJ-42-e113246-s015.zip › Figure 3/3A/Trem2 CV primary microglia brightfield.tif]

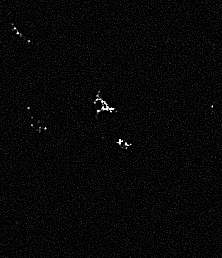

Supplement: Supplementary file 13 — Source Data for Figure 3 [file EMBJ-42-e113246-s015.zip › Figure 3/3A/Trem2 R47H Ctrl-SN.tif]

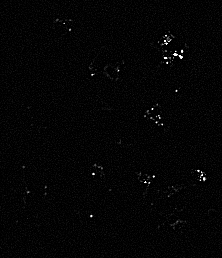

Supplement: Supplementary file 13 — Source Data for Figure 3 [file EMBJ-42-e113246-s015.zip › Figure 3/3A/Trem2 R47H oABeta-SN.tif]

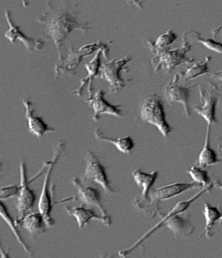

Supplement: Supplementary file 13 — Source Data for Figure 3 [file EMBJ-42-e113246-s015.zip › Figure 3/3A/Trem2 R47H primary microglia brightfield.tif]

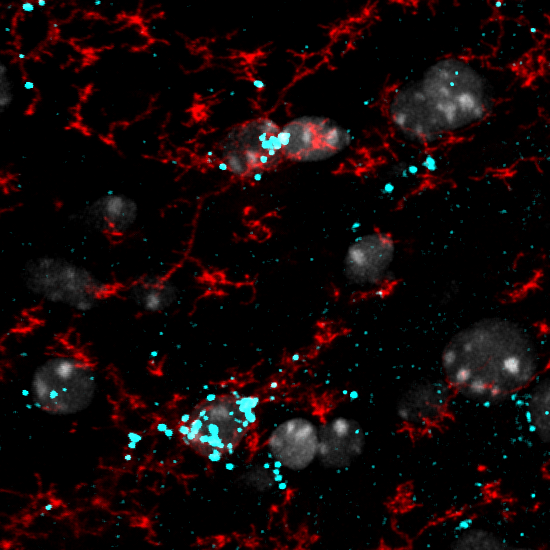

Supplement: Supplementary file 13 — Source Data for Figure 3 [file EMBJ-42-e113246-s015.zip › Figure 3/3D/Fig3D_NLF;CV_CSR1_Iba1;C1q.tif]

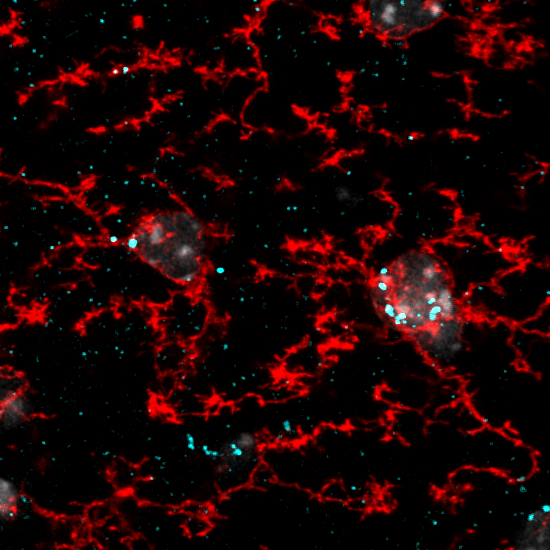

Supplement: Supplementary file 13 — Source Data for Figure 3 [file EMBJ-42-e113246-s015.zip › Figure 3/3D/Fig3D_NLF;R47H_CSR1_Iba1;C1q.tif]

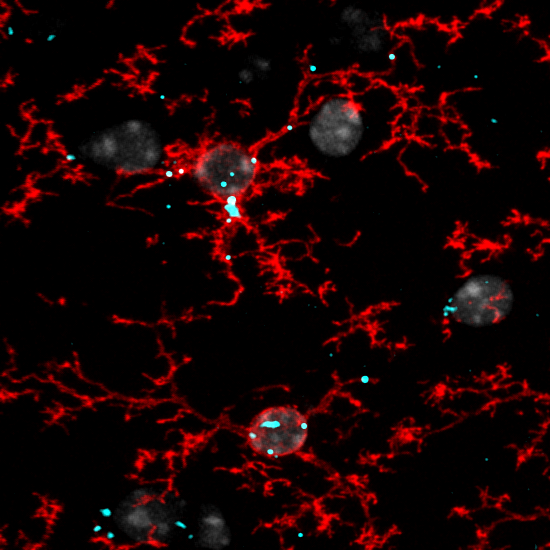

Supplement: Supplementary file 13 — Source Data for Figure 3 [file EMBJ-42-e113246-s015.zip › Figure 3/3D/Fig3D_WT;CV_CSR1_Iba1;C1q.tif]

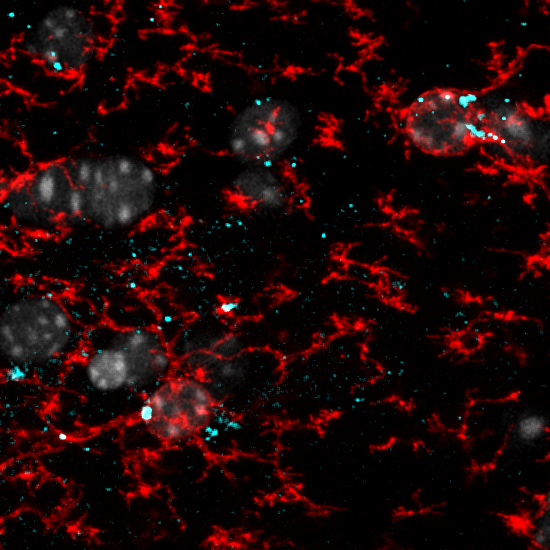

Supplement: Supplementary file 13 — Source Data for Figure 3 [file EMBJ-42-e113246-s015.zip › Figure 3/3D/Fig3D_WT;R47H_CSR1_Iba1;C1q.tif]

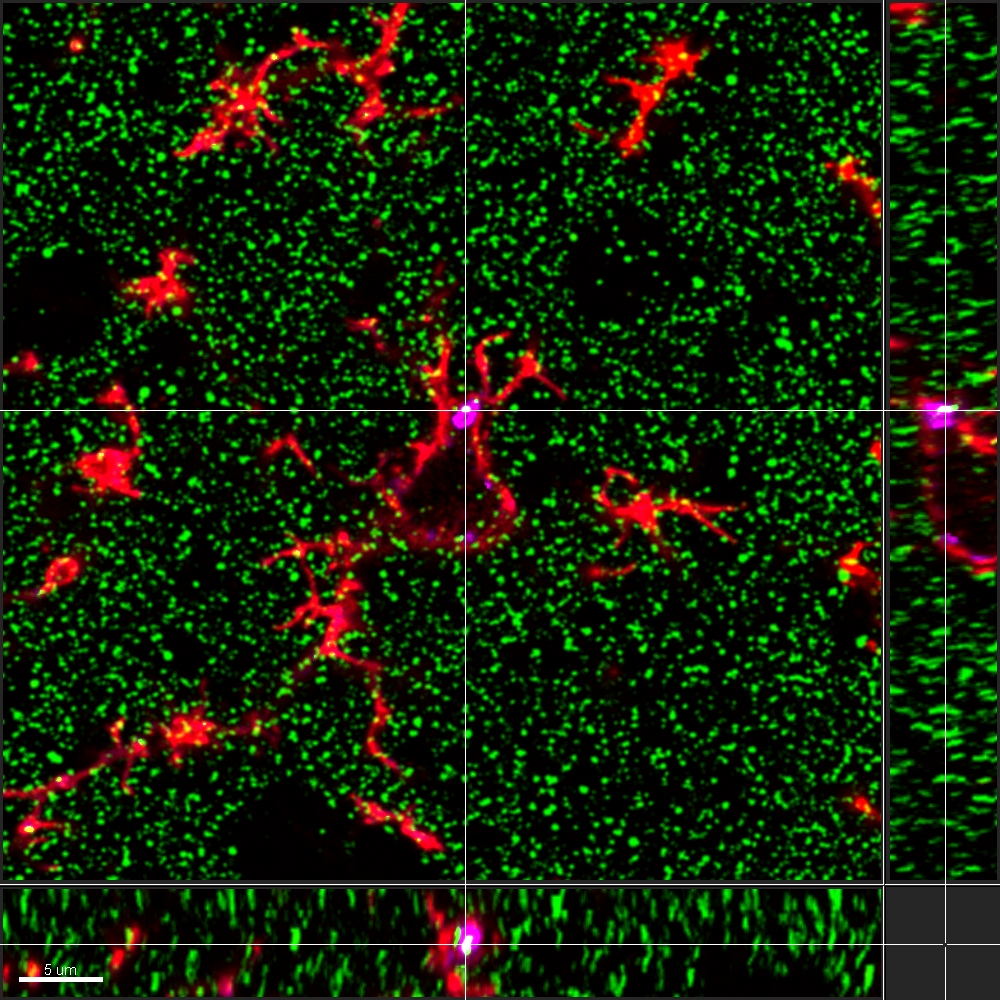

Supplement: Supplementary file 13 — Source Data for Figure 3 [file EMBJ-42-e113246-s015.zip › Figure 3/3E/Fig3E_Homer1;P2Y12;CD68_Overview.jpg]

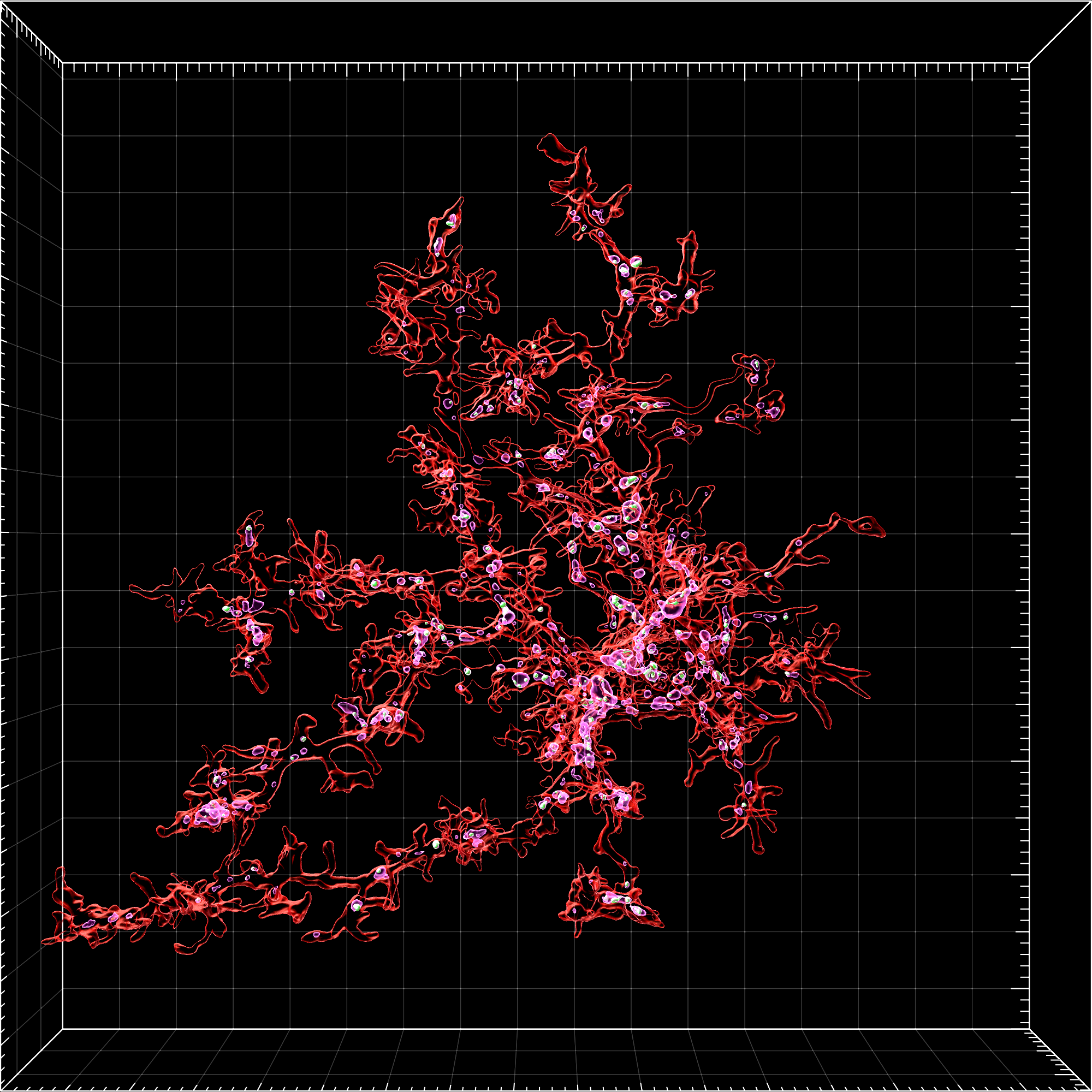

Supplement: Supplementary file 13 — Source Data for Figure 3 [file EMBJ-42-e113246-s015.zip › Figure 3/3G/Fig3G_NLF;CV_Homer1;P2Y12;CD68.tif]

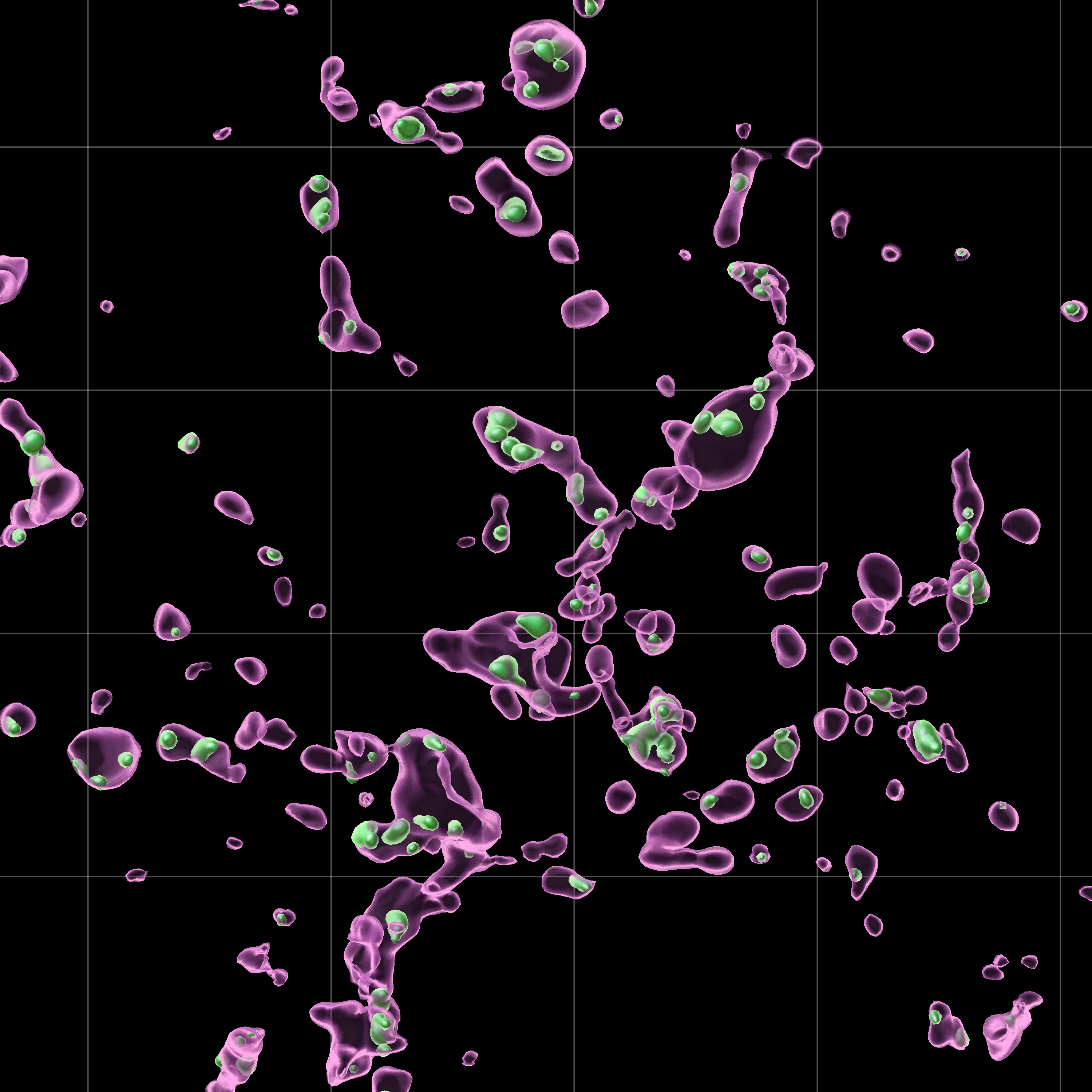

Supplement: Supplementary file 13 — Source Data for Figure 3 [file EMBJ-42-e113246-s015.zip › Figure 3/3G/Fig3G_NLF;CV_Inset_Homer1;CD68.tif]

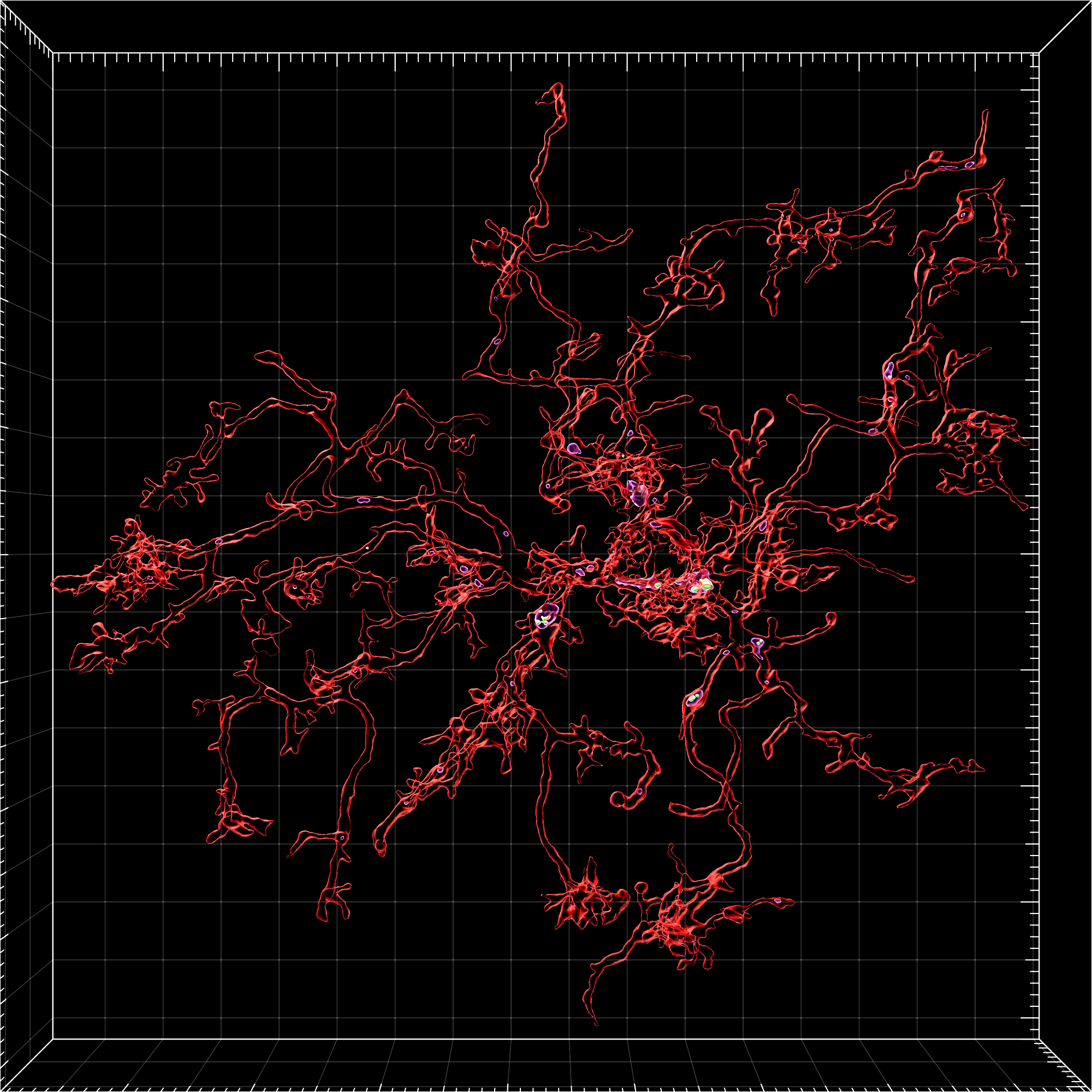

Supplement: Supplementary file 13 — Source Data for Figure 3 [file EMBJ-42-e113246-s015.zip › Figure 3/3G/Fig3G_NLF;R47H_Homer1;P2Y12;CD68.tif]

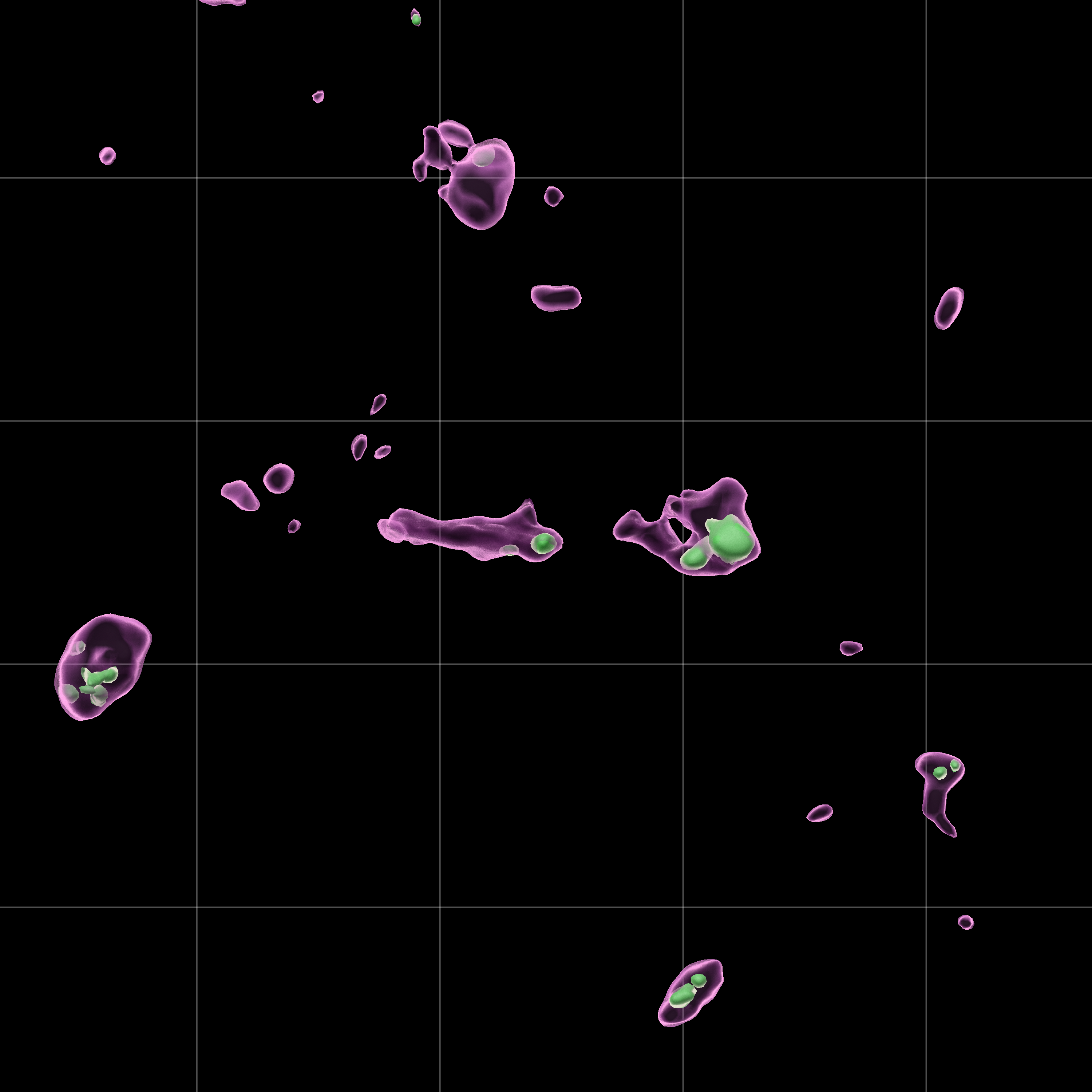

Supplement: Supplementary file 13 — Source Data for Figure 3 [file EMBJ-42-e113246-s015.zip › Figure 3/3G/Fig3G_NLF;R47H_Inset_Homer1;CD68.tif]

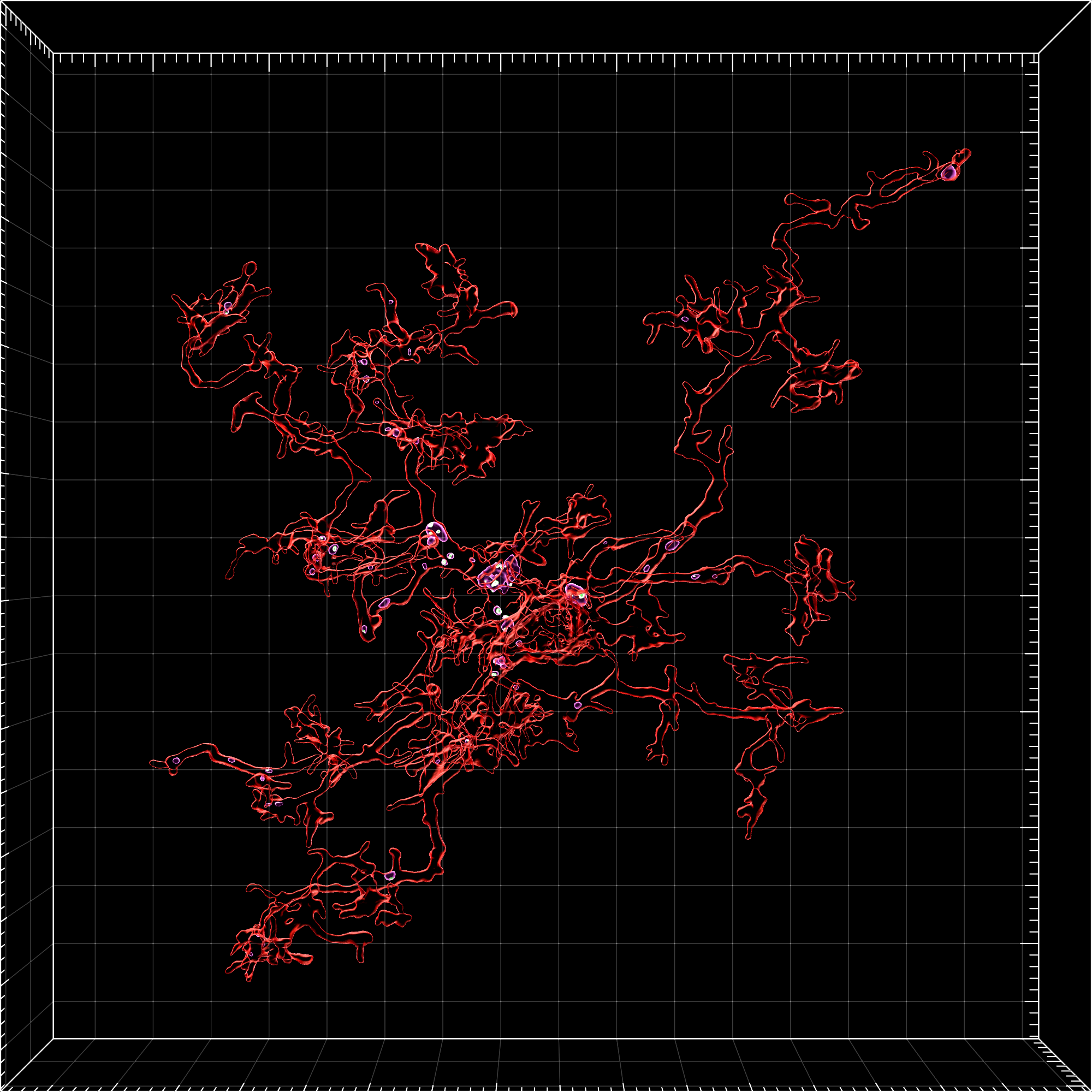

Supplement: Supplementary file 13 — Source Data for Figure 3 [file EMBJ-42-e113246-s015.zip › Figure 3/3G/Fig3G_WT;CV_Homer1;P2Y12;CD68.tif]

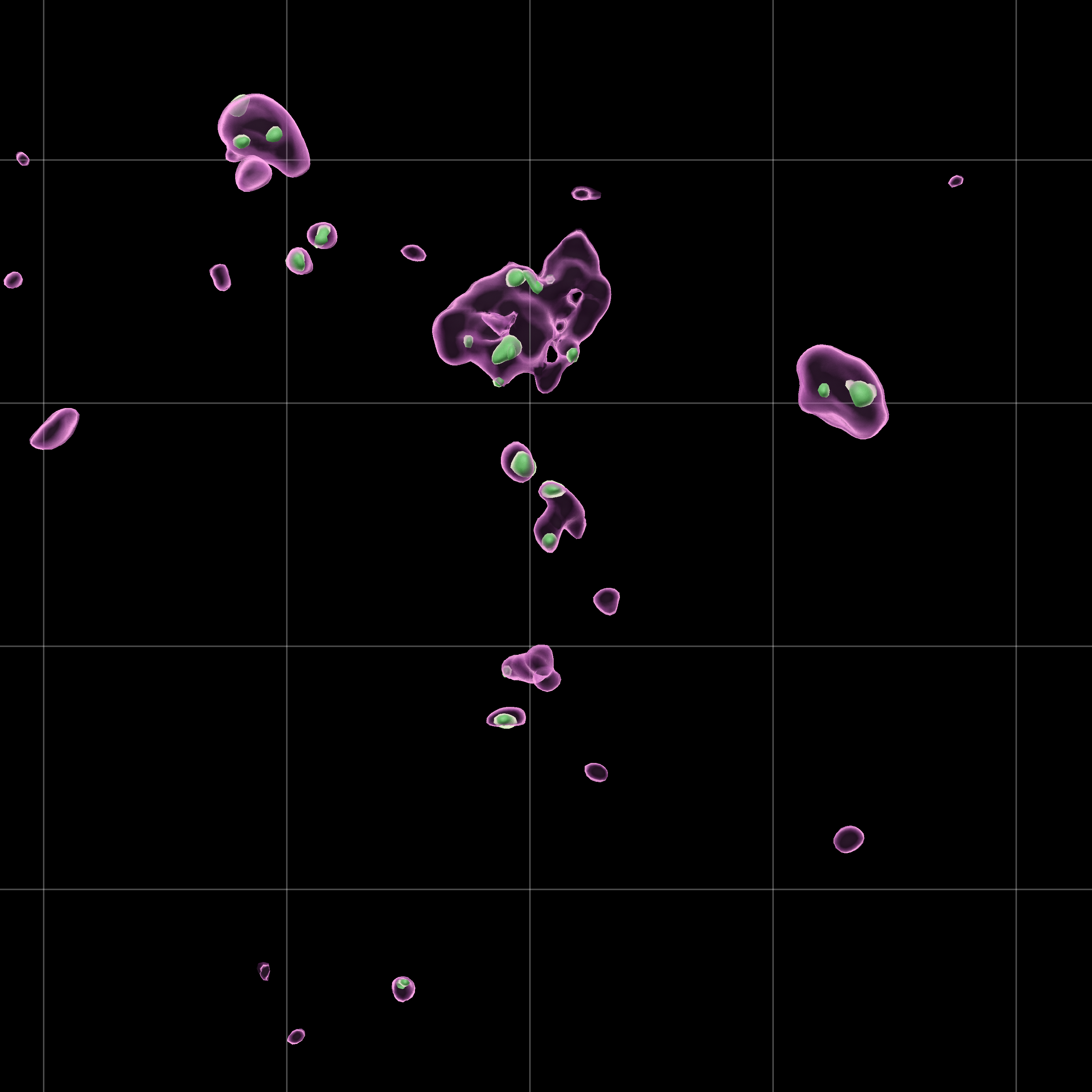

Supplement: Supplementary file 13 — Source Data for Figure 3 [file EMBJ-42-e113246-s015.zip › Figure 3/3G/Fig3G_WT;CV_Inset_Homer1;CD68.tif]

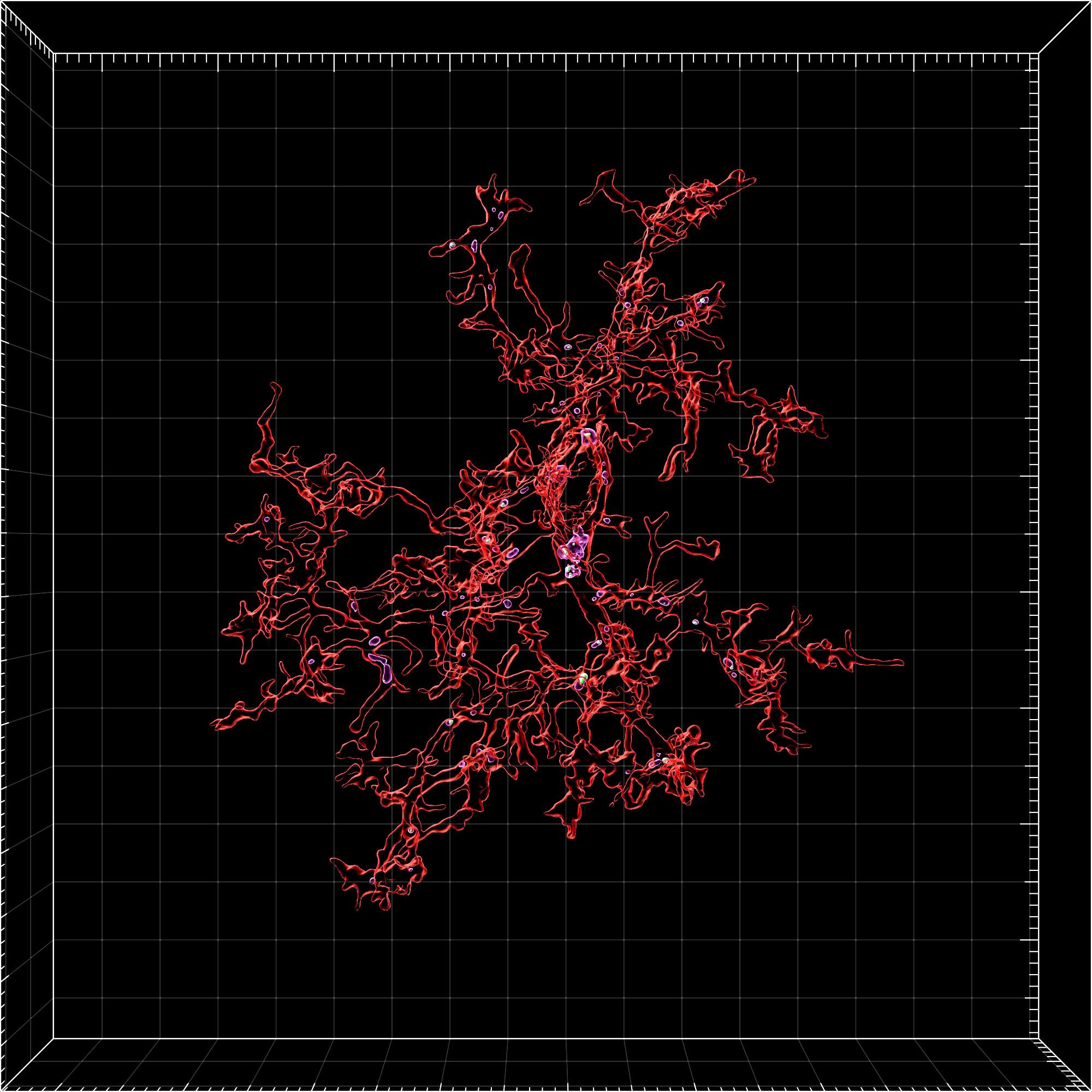

Supplement: Supplementary file 13 — Source Data for Figure 3 [file EMBJ-42-e113246-s015.zip › Figure 3/3G/Fig3G_WT;R47H_Homer1;P2Y12;CD68.tif]

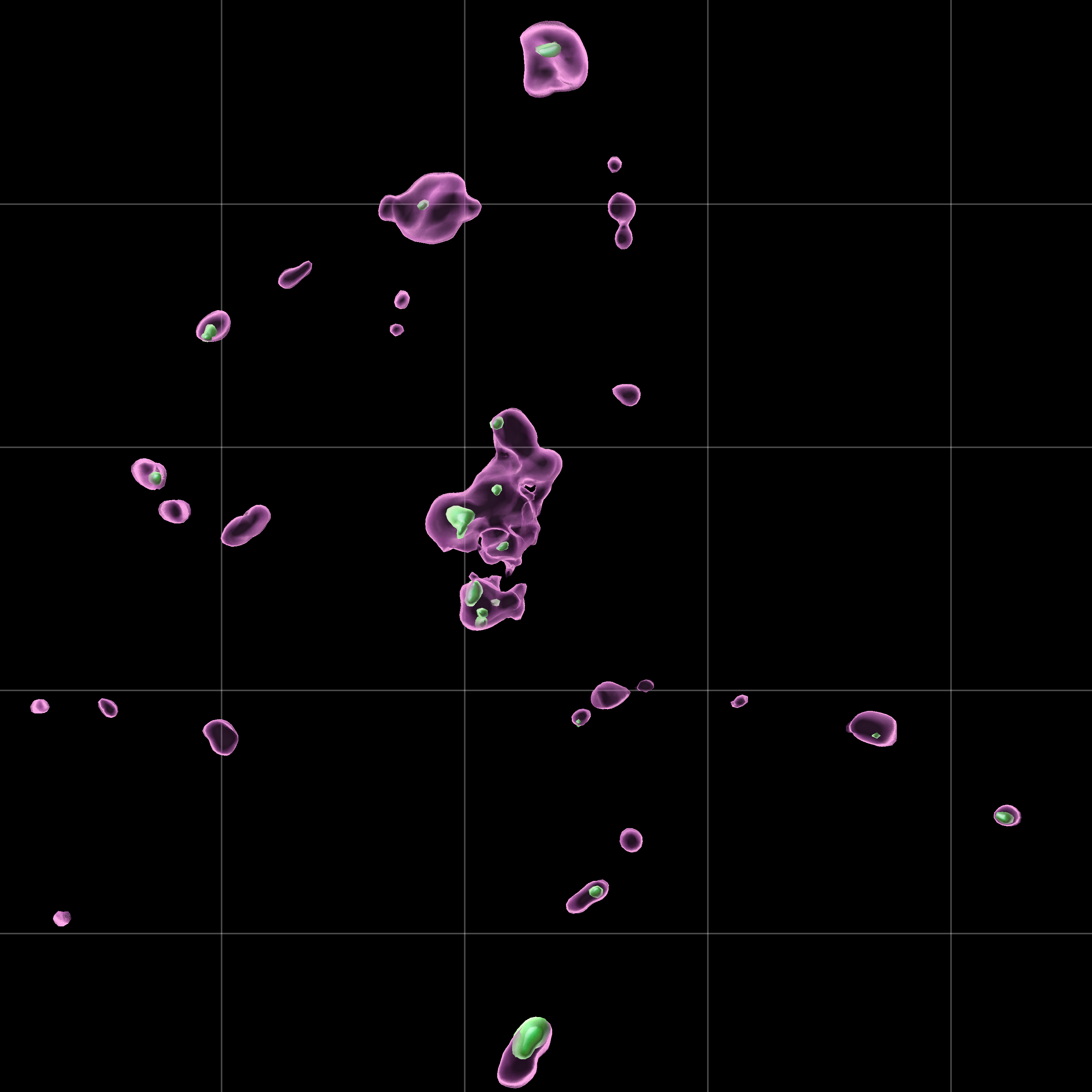

Supplement: Supplementary file 13 — Source Data for Figure 3 [file EMBJ-42-e113246-s015.zip › Figure 3/3G/Fig3G_WT;R47H_Inset_Homer1;CD68.tif]

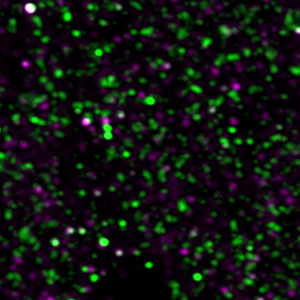

Supplement: Supplementary file 13 — Source Data for Figure 3 [file EMBJ-42-e113246-s015.zip › Figure 3/3I/Fig3I_MAX_NLF;CV_Homer1;Tagmin(RGB).tif]

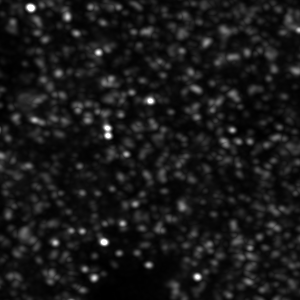

Supplement: Supplementary file 13 — Source Data for Figure 3 [file EMBJ-42-e113246-s015.zip › Figure 3/3I/Fig3I_MAX_NLF;CV_Homer1;Tagmin.tif]

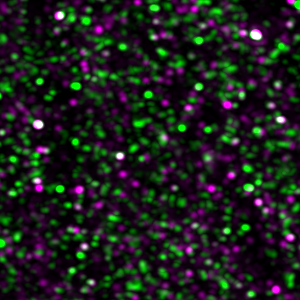

Supplement: Supplementary file 13 — Source Data for Figure 3 [file EMBJ-42-e113246-s015.zip › Figure 3/3I/Fig3I_MAX_NLF;R47H_Homer1;Tagmin(RGB).tif]

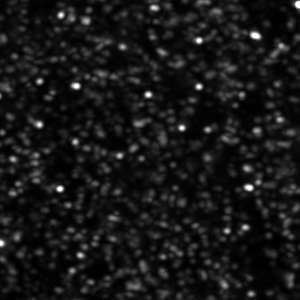

Supplement: Supplementary file 13 — Source Data for Figure 3 [file EMBJ-42-e113246-s015.zip › Figure 3/3I/Fig3I_MAX_NLF;R47H_Homer1;Tagmin.tif]

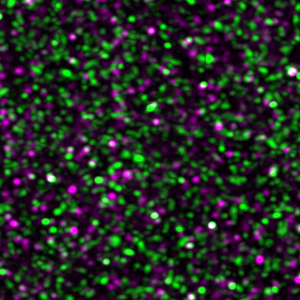

Supplement: Supplementary file 13 — Source Data for Figure 3 [file EMBJ-42-e113246-s015.zip › Figure 3/3I/Fig3I_MAX_WT;CV_Homer1;Tagmin(RGB).tif]

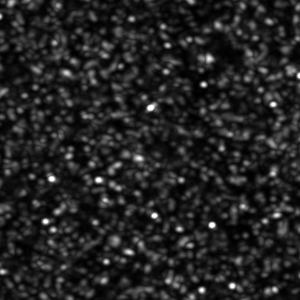

Supplement: Supplementary file 13 — Source Data for Figure 3 [file EMBJ-42-e113246-s015.zip › Figure 3/3I/Fig3I_MAX_WT;CV_Homer1;Tagmin.tif]

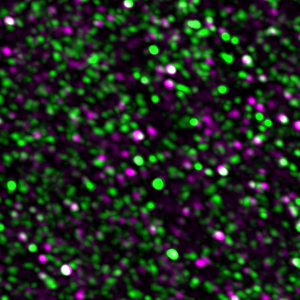

Supplement: Supplementary file 13 — Source Data for Figure 3 [file EMBJ-42-e113246-s015.zip › Figure 3/3I/Fig3I_MAX_WT;R47H_Homer1;Tagmin(RGB).tif]

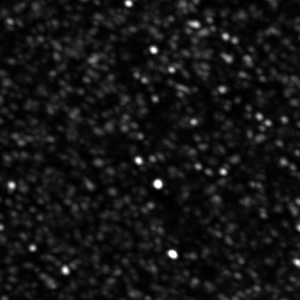

Supplement: Supplementary file 13 — Source Data for Figure 3 [file EMBJ-42-e113246-s015.zip › Figure 3/3I/Fig3I_MAX_WT;R47H_Homer1;Tagmin.tif]

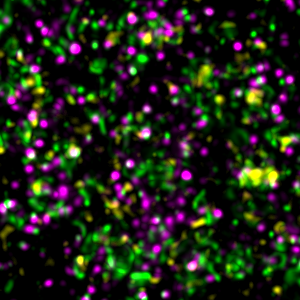

Supplement: Supplementary file 14 — Source Data for Figure 4 [file EMBJ-42-e113246-s005.zip › Figure 4/4A/Fig4A_MAX_NLF;CV_Homer1;Tagmin;PSVue(GMY).tif]

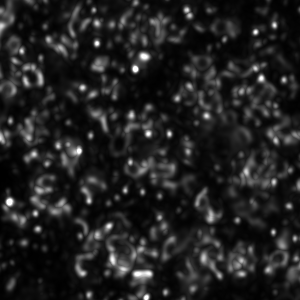

Supplement: Supplementary file 14 — Source Data for Figure 4 [file EMBJ-42-e113246-s005.zip › Figure 4/4A/Fig4A_MAX_NLF;CV_Homer1;Tagmin;PSVue.tif]

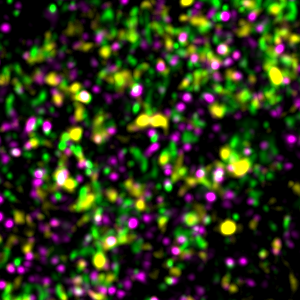

Supplement: Supplementary file 14 — Source Data for Figure 4 [file EMBJ-42-e113246-s005.zip › Figure 4/4A/Fig4A_MAX_NLF;R47H_Homer1;Tagmin;PSVue(GMY).tif]

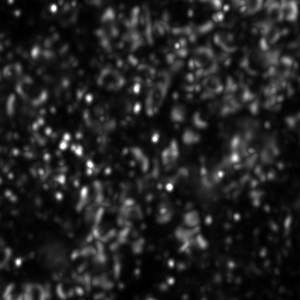

Supplement: Supplementary file 14 — Source Data for Figure 4 [file EMBJ-42-e113246-s005.zip › Figure 4/4A/Fig4A_MAX_NLF;R47H_Homer1;Tagmin;PSVue.tif]

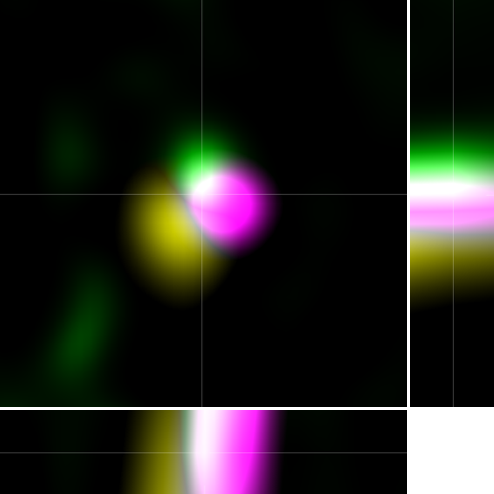

Supplement: Supplementary file 14 — Source Data for Figure 4 [file EMBJ-42-e113246-s005.zip › Figure 4/4C/Figure 4C.tif]

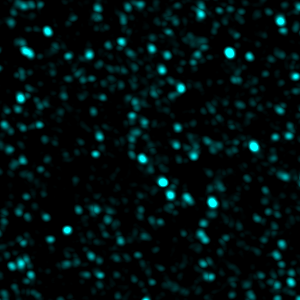

Supplement: Supplementary file 14 — Source Data for Figure 4 [file EMBJ-42-e113246-s005.zip › Figure 4/4F/Fig4F_MAX_NLF;CV_Bassoon.tif]

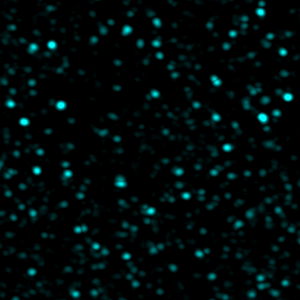

Supplement: Supplementary file 14 — Source Data for Figure 4 [file EMBJ-42-e113246-s005.zip › Figure 4/4F/Fig4F_MAX_NLF;R47H_Bassoon.tif]

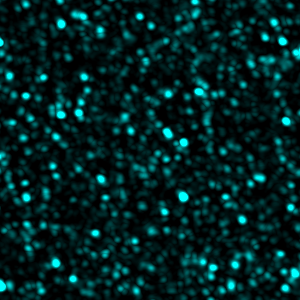

Supplement: Supplementary file 14 — Source Data for Figure 4 [file EMBJ-42-e113246-s005.zip › Figure 4/4F/Fig4F_MAX_WT;CV_Bassoon.tif]

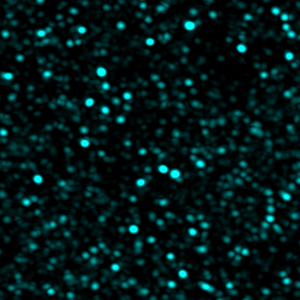

Supplement: Supplementary file 14 — Source Data for Figure 4 [file EMBJ-42-e113246-s005.zip › Figure 4/4F/Fig4F_MAX_WT;R47H_Bassoon.tif]

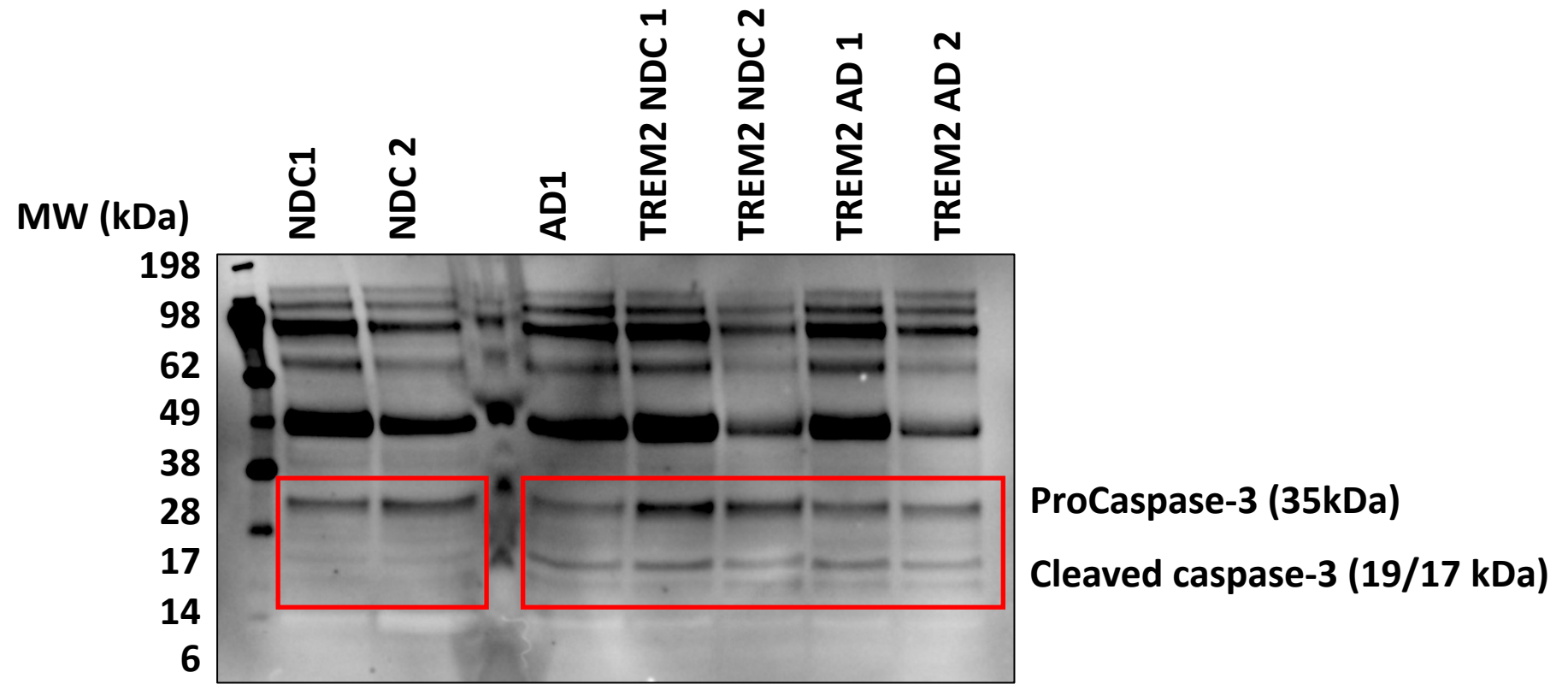

Supplement: Supplementary file 14 — Source Data for Figure 4 [file EMBJ-42-e113246-s005.zip › Figure 4/4G/Caspase-3 annotated.pdf]

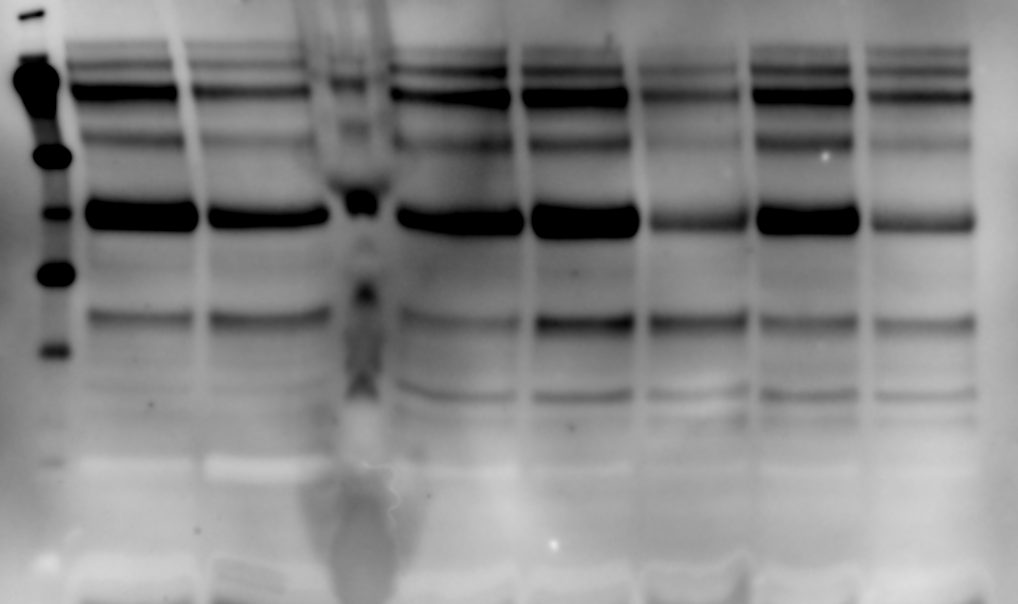

Supplement: Supplementary file 14 — Source Data for Figure 4 [file EMBJ-42-e113246-s005.zip › Figure 4/4G/Caspase-3.tif]

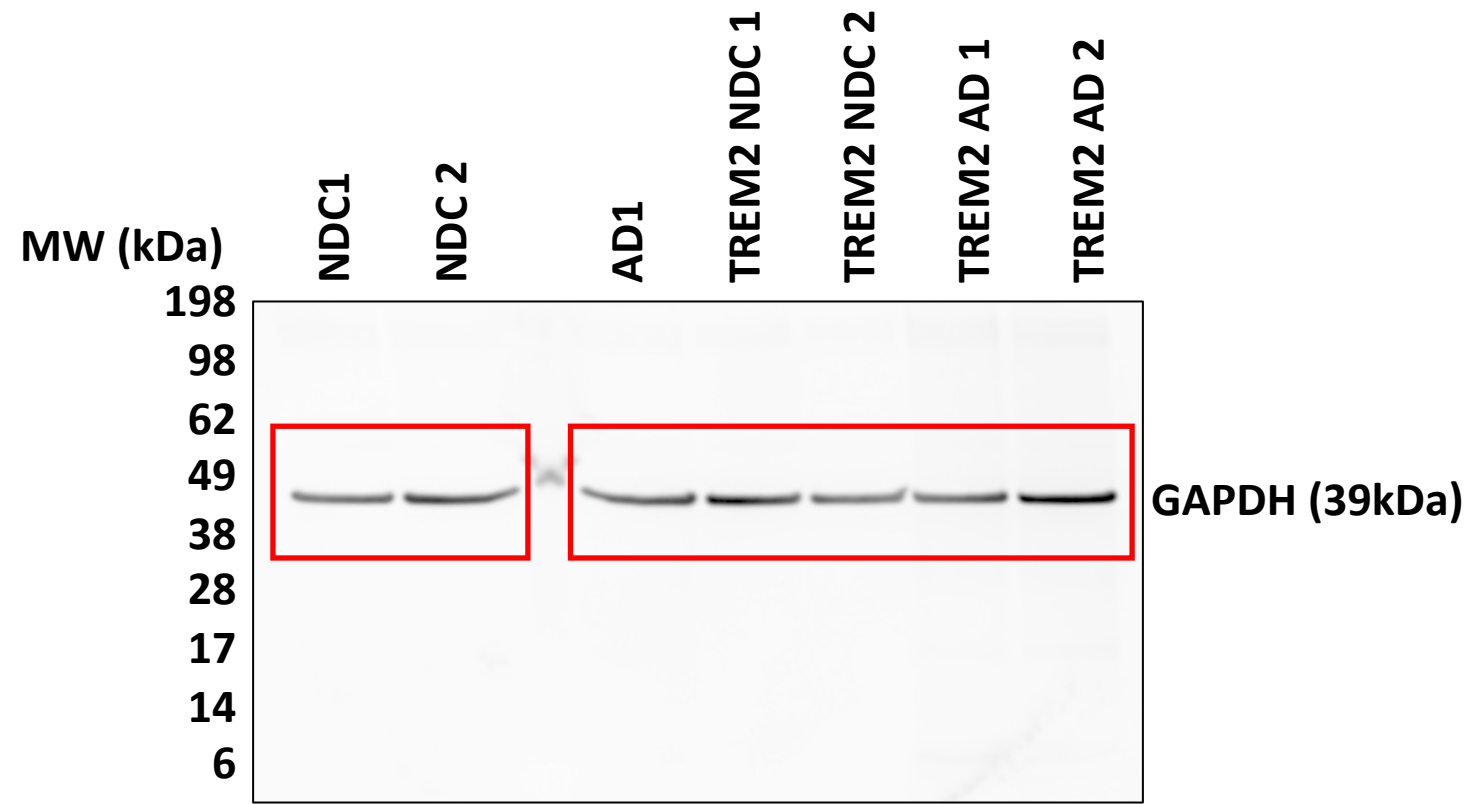

Supplement: Supplementary file 14 — Source Data for Figure 4 [file EMBJ-42-e113246-s005.zip › Figure 4/4G/GAPDH annotated.pdf]

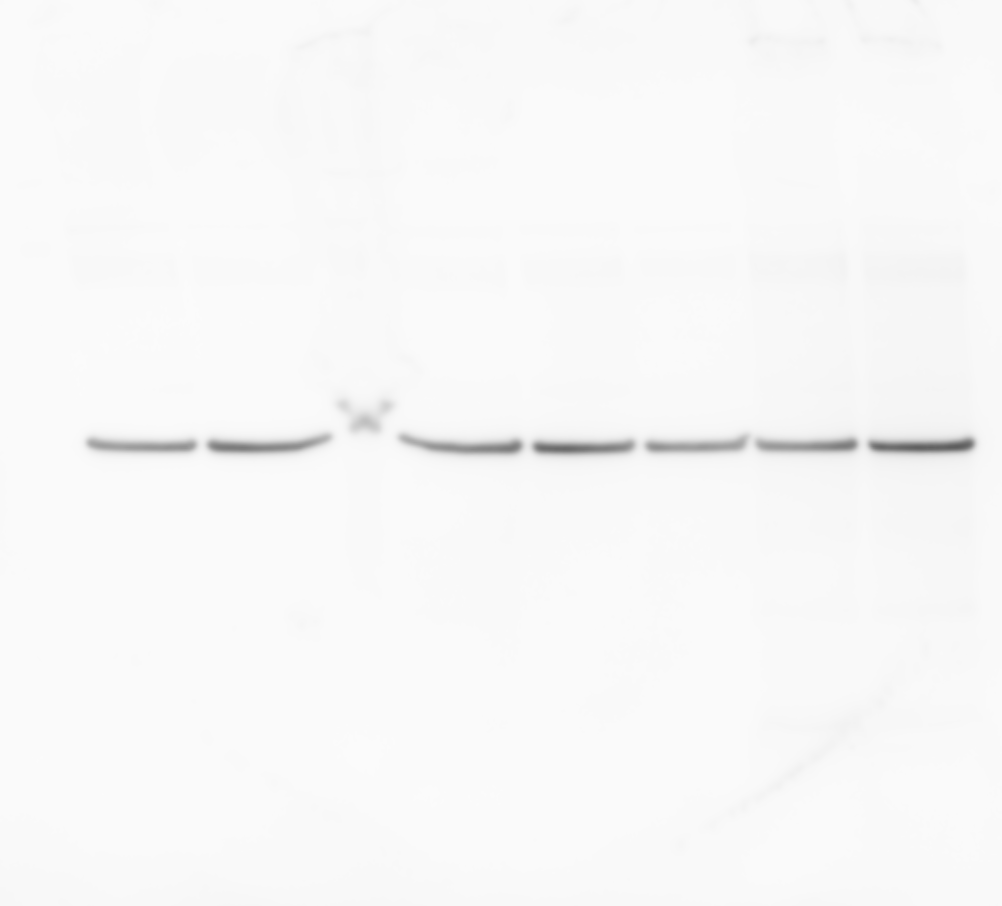

Supplement: Supplementary file 14 — Source Data for Figure 4 [file EMBJ-42-e113246-s005.zip › Figure 4/4G/GAPDH.tif]
