# Supplementary material for: Microglia‐synapse engulfment via PtdSer‐TREM2 ameliorates neuronal hyperactivity in Alzheimer's disease models
Source: EMBO J. 2023 Aug 14;42(19):e113246. doi: 10.15252/embj.2022113246 (PMC10548173; doi:10.15252/embj.2022113246)
Supplement: Supplementary file 15 [file EMBJ-42-e113246-s002.pdf]

THE EMBO JOURNAL

# Microglia-synapse engulfment via PtdSer-TREM2 ameliorates neuronal hyperactivity in Alzheimer's disease models

Javier Rueda-Carrasco, Dimitra Sokolova, Sang-Eun Lee, Thomas Childs, Natália Jurčáková, Gerard Crowley, Sebastiaan De Schepper, Judy Z. Ge, Joanne I. Lachica, Christina E. Toomey, Oliver J. Freeman, John Hardy, Samuel J. Barnes, Tammaryn Lashley, Beth Stevens, Sunghoe Chang and Soyon Hong
**DOI: 10.15252/embj.2022113246**

*Corresponding author(s): Soyon Hong (soyon.hong@ucl.ac.uk) , Javier Rueda-Carrasco (j.rueda-carrasco@ucl.ac.uk)*

## Review Timeline:

| | |
|---|---|
| Submission Date: | 12th Dec 22 |
| Editorial Decision: | 23rd Jan 23 |
| Revision Received: | 25th May 23 |
| Editorial Decision: | 25th Jun 23 |
| Revision Received: | 20th Jul 23 |
| Accepted: | 25th Jul 23 |

*Editor: Karin Dumstrei*

## Transaction Report:

(Note: With the exception of the correction of typographical or spelling errors that could be a source of ambiguity, letters and reports are not edited. Depending on transfer agreements, referee reports obtained elsewhere may or may not be included in this compilation. Referee reports are anonymous unless the Referee chooses to sign their reports.)

Dear Soyon,

Thank you for submitting your manuscript to The EMBO Journal. Your study has now been seen by two referees and their comments are provided below.

As you can see, both referees appreciate the analysis and support publication here. They raise some constructive comments that would strengthen the analysis.

I think it would be helpful to discuss the raised points further and I am available to do so via email or video.

When preparing your letter of response to the referees' comments, please bear in mind that this will form part of the Review Process File, and will therefore be available online to the community. For more details on our Transparent Editorial Process, please visit our website: https://www.embopress.org/page/journal/14602075/authorguide#transparentprocess

I thank you for the opportunity to consider your work for publication. I look forward to discussing your revisions further.

Yours sincerely,

Karin

Karin Dumstrei, PhD
Senior Editor
The EMBO Journal

Instructions for preparing your revised manuscript:

Please make sure you upload a letter of response to the referees' comments together with the revised manuscript.

Please also check that the title and abstract of the manuscript are brief, yet explicit, even to non-specialists.

When assembling figures, please refer to our figure preparation guideline in order to ensure proper formatting and readability in print as well as on screen:
https://bit.ly/EMBOPressFigurePreparationGuideline
See also guidelines for figure legends: https://www.embopress.org/page/journal/14602075/authorguide#figureformat

At EMBO Press we ask authors to provide source data for the main manuscript figures. Our source data coordinator will contact you to discuss which figure panels we would need source data for and will also provide you with helpful tips on how to upload and organize the files.

IMPORTANT: When you send the revision we will require
- a point-by-point response to the referees' comments, with a detailed description of the changes made (as a word file).
- a word file of the manuscript text.
- individual production quality figure files (one file per figure)
- a complete author checklist, which you can download from our author guidelines (https://www.embopress.org/page/journal/14602075/authorguide).
- Expanded View files (replacing Supplementary Information)

Please see out instructions to authors
https://www.embopress.org/page/journal/14602075/authorguide#expandedview

Please remember: Digital image enhancement is acceptable practice, as long as it accurately represents the original data and conforms to community standards. If a figure has been subjected to significant electronic manipulation, this must be noted in the figure legend or in the 'Materials and Methods' section. The editors reserve the right to request original versions of figures and the original images that were used to assemble the figure.

Further information is available in our Guide For Authors: https://www.embopress.org/page/journal/14602075/authorguide

We realize that it is difficult to revise to a specific deadline. In the interest of protecting the conceptual advance provided by the work, we recommend a revision within 3 months (23rd Apr 2023). Please discuss the revision progress ahead of this time with

the editor if you require more time to complete the revisions.

As a matter of policy, competing manuscripts published during this period will not negatively impact on our assessment of the conceptual advance presented by your study. However, we request that you contact the editor as soon as possible upon publication of any related work, to discuss how to proceed.

------------------------------------------------

Referee #1:

This is a compelling manuscript that addresses the question of how synapses are targeted for phagocytic clearance. The present study is a logical extension of their previous work implicating ePtdSer in targeting of synapses for phagocytic removal. Inclusion of studies with human material provides an important validation of the underlying mechanism. However, while the study dissects the involvement of ePtdSer its does not dissect the mechanisms through which oA  acts to promote its appearance.
Overall, this substantive study that elicits considerable enthusiasm.

Specific comments:
The roles of complement have dominated the discussion of synaptosis in the context of both developmental pruning and in degenerative disease. Missing from the manuscript is any discussion of how the TREM2/ePtdSer mechanisms intersect with those involving the complement system.

The authors, and others, have the documented the ability of C1q to associate with ePtsSer and the complex was described as an 'eat me' signal. The introduction would benefit from moving up the discussion of how and under what circumstances PtdSer is externalized on the membrane.

They adopted a clever experimental design whereby they demonstrated that spines exhibiting ePtdSer had greater calcium transient activity and are preferentially targeted for phagocytic clearance with a consequent reduction in calcium activity. Does complement play any role in this sequence of events - as might be expected from the literature and their previous work?

Is the ability of oA  to provoke the appearance of ePtsSer on synaptosomes a unique property of Ab or will other stimuli elicit the same effect, e.g. other agents that drive Ca transients and depolarization? Is the effect of oA  on PtdSer externalization reliant upon the Ca transients?

In Fig 3C/E they evaluated P2RY12+ microglia. Was the same elevation of lysosomal Homer + puncta also elevated in TREM2+/CLEC7A+ , microglia (which should be found in the 6 mo mice) or is this a unique effect in the homeostatic microglia.

In Fig 4F/G the conclusion that TREM2 LOF variants leads to increased apoptotic synapses is based on examination of 2 individuals that are stated to carry TREM2 variants. The variants are not identified and it is unknown. whether they were similar in the two samples. I think it is inappropriate on this basis to conclude that overall brain active caspase 3 levels in synaptosomes are associated with the abundance of ePtdSer + synapses. These data should be removed from the manuscript.

Minor points:
The quality of the images provided in Figs 3E and S1D is poor. It is not clear whether this is due to incorporation of the panels into the pdf or that the original images also lack contrast.

Fig S1 is problematic. There is a large variance in Ab levels between the AD samples shown. The standard curve in panel E appears to be non-linear, and compares poorly with a comparable blot in Fig S2 Panel D. There is no signal visible in the lower panels in the images provided to me. The image size is too small to resolve the cells in the upper panel.

Referee #2:

In this manuscript, Rueda-Carrasco, Sokolova, and Lee et al. aim to show how microglia resolve neuronal hyperactivity by selectively engulfing damaged synapses in the earliest stages of Alzheimer's disease. Although synaptic loss induced by oligomeric Aß and synaptic engulfment by microglia occurs in mouse models of amyloidosis, the molecular mechanisms by which healthy microglia preferentially target synapses for removal in the pre-plaque-enriched AD brain are not well understood.

Using a combination of super-resolution microscopy and live imaging approaches on in vitro co-cultures and in vivo genetic mouse models, the authors show that dendrites externalize phosphatidylserine (PtdSer) upon exposure to oligomeric Aß. They link microglia engulfment of PtdSer+ synapses in response to Aß exposure to TREM2 and aim to demonstrate this resolves Aß-induced neuronal hyperactivity. The work is largely compelling and answers a key question that is of broad interest to the general neuroscience and AD fields. There are two major and a few minor concerns that if addressed would greatly improve the impact of their findings and better support their conclusions.

Major Concerns:

1. Some of these images do not compellingly demonstrate the selective nature of elimination of hyperactive spines. While preferential engulfment of PSVue+ spines are striking in Figure 2A, contact and targeting appears non-discriminatory in Figure S5A perhaps because these still images do not illustrate the 3D nature of the data. As it is currently represented, there appear to be more PSVue- dendritic spines that are targeted while the microglia is homing to the PSVue+ spine. It may further emphasize the point to mark the hyperactive spines with an arrow in the movies. This applies to Figure S5A and Movie S2 & S3.
2. Collectively, the data presented in this paper implicate TREM2 in resolution of Aß-induced neuronal hyperactivity by mediating selective engulfment of Aß damaged, PtdSer+ synapses in microglia. While the in vitro co-culture and the in vivo Trem2 R47H KI mouse experiments in Figure 3 & 4 demonstrate the importance of TREM2 for preferential engulfment of PtdSer+ synapses, data linking TREM2 to resolution of neuronal hyperactivity is lacking.
a. Can primary Trem2 R47H KI microglia, when co-cultured with GCaMP7 transfected neurons, resolve neuronal hyperactivity 48 hours post oligomeric Aß challenge?
b. The gold standard for measuring hyperexcitability in this type of model is electrophysiologically. The killer experiment would be to perform slice physiological studies of the hippocampi of mice from the NL-F/TREM2 mice compared with controls to show that this axis is true in vivo. This is not strictly necessary but would go far to strengthen the conclusions.

Minor Concerns:
-There is some concern for pseudoreplication. Please highlight how each N was considered for statistical tests for Figure 1, 4, S2, S3, S5. For statistics in Figure S5, how were significant tests done, as an N as each ROI or per each biological N?
-Recommend in the abstract/introduction replacing the term "apoptotic" synapses with "synaptotic" or "apoptotic-like" synapses consistently for clarity
-Formatting:
There are a few typos populated throughout the text (full stop on page 7 line 25, GCaMP7 on page 15 line 33, hours on page 35 line 4, Synaptotagmin on page 36 line 8)
Please introduce reagents (Annexin V, PSVue, etc.) as they first appear in the text (i.e. background statement on Annexin V appears on page 8 even though it first appeared on page 5)
Please add arrowheads to highlight a few representative PtdSer+ vs. PtdSer- spines in Movie S5.
Movies are currently mislabeled (i.e. Movie S1 was labeled and uploaded as Movie 3)

### Letter of response to the referees' comments regarding our manuscript:

### Rueda-Carrasco *et al.,* Microglia-synapse engulfment via PtdSer-TREM2 ameliorates neuronal hyperactivity in β-amyloid models

We would like to thank the reviewers for their positive reception of our work and for their in-depth and constructive review of our manuscript. We believe that our new data and edits to the revised paper address the main concerns and thus significantly strengthen the paper. Please see below our point-by-point response to each reviewer in blue. Please also find the amended versions of the manuscript, one clean version and one version with the amendments highlighted in yellow.

## Comments and Responses to Referee #1

This is a compelling manuscript that addresses the question of how synapses are targeted for phagocytic clearance. The present study is a logical extension of their previous work implicating ePtdSer in targeting of synapses for phagocytic removal. Inclusion of studies with human material provides an important validation of the underlying mechanism. However, while the study dissects the involvement of ePtdSer its does not dissect the mechanisms through which oAb acts to promote its appearance. Overall, this substantive study that elicits considerable enthusiasm.

We thank the Reviewer for this supporting comment.

**1**) The roles of complement have dominated the discussion of synaptosis in the context of both developmental pruning and in degenerative disease. Missing from the manuscript is any discussion of how the TREM2/ePtdSer mechanisms intersect with those involving the complement system.

We agree with the Reviewer the importance of this link; we have now added in Discussion as well as an additional experiment using RNAScope to further probe link between C1q and TREM2.

Re: TREM2 and C1q: Previous studies suggest that C1q upregulation in amyloid plaque-responsive microglia as well as tau models is downstream of TREM2 (Keren-Shaul et al., Cell 2017, doi: 10.1016/j.cell.2017.05.018; Wood et al., Cell Reports 2022, doi:10.1016/j.celrep.2022.111686; Gratuze et al., JCI 2020, doi: 10.1172/JCI138179). Here we specifically focus on pre-plaque stages when synapses are already vulnerable to microglial C1q deposition and elimination (De Schepper et al., Nat Neurosci 2023, doi: 10.1038/s41593-023-01257-z). To test whether C1q upregulation in pre-plaque amyloid pathology in hAPP-NL-F knockin (KI) mice (6 mo) is also downstream of TREM2 as has been shown in other stages and mouse models, we performed RNAScope, which allows for single molecule fluorescent *in situ* hybridization (smFISH) for *C1qa* mRNA levels, combined with immunohistochemistry (IHC) to stain for Iba1$^{+}$ microglia/macrophage cells including their soma and processes. We then quantified the levels of *C1qa* mRNA levels in the hippocampus of 4 genotypes at 6 months of age: WT, NL-F, Trem2 KI and NL-F;Trem2 KI mice. Whereas there is an increase of *C1qa* mRNA in 6 mo NL-F hippocampus compared to those of WT mice, akin to our recent observation (De Schepper et al., Nat Neurosci 2023, doi: 10.1038/s41593-023-01257-z), there is no difference in *C1qa* levels between Trem2 KI and NL-F;Trem2 KI mice (N=3-4 mice/genotype). We have now added these data as part of **Fig 3C** and **D**. This new data, along with synaptic engulfment data (**Fig 3E**-**G**), suggest that TREM2 function is required for microglia-synapse engulfment involving C1q.

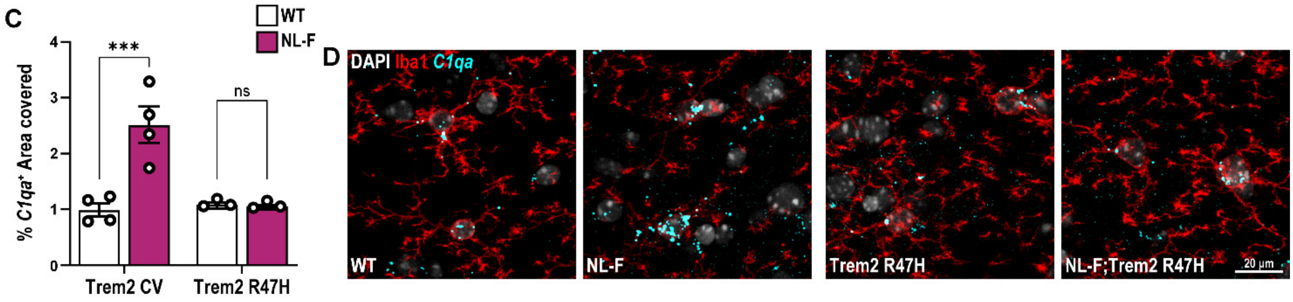


Re: C1q and ePtdSer: A previous elegant study has shown that there are apoptotic-like changes on C1q-bound synaptosomes (please see Györffy et al., PNAS 2018, doi: 10.1073/pnas.1722613115). As Reviewer suggested, we have now added this paragraph in Discussion on the link between C1q and ePtdSer/TREM2 (line 24 on page 13 in Main Text):

*"*Our data further suggest that TREM2 may also act upstream of C1q-mediated microglia-synapse engulfment in amyloid models, in line with previous study in a tau-model of AD (Gratuze et al., 2020). We and others have previously shown that microglia eliminate synapses in a complement-dependent manner in Aβ- and tau-based models (Hong et al., 2016; Dejanovic et al., 2018); however, upstream mediators of which synapses are targeted were not known. Interestingly, C1q has been highlighted as putative binding partner of ePtdSer (Païdassi et al., 2008) and proteomics analysis suggests that C1q-tagged synaptosomes are enriched in apoptotic-like features (Györffy et al., 2018). Altogether these studies suggest that TREM2-ePtdSer-C1q may converge on the same pathway to mediate microglia-synapse elimination. The exact mechanisms of how TREM2 modulates synapse phagocytosis as well as the functional consequences of ePtdSer-TREM2 microglia-synapse engulfment in the plaque-enriched, aged brains are to be determined*".*

**2**) The authors, and others, have the documented the ability of C1q to associate with ePtsSer and the complex was described as an 'eat me' signal. The introduction would benefit from moving up the discussion of how and under what circumstances PtdSer is externalized on the membrane.

Per Reviewer's suggestion, we have now moved up this text to be in Introduction as well as additional text to explain when and where PtdSer is externalised (line 23 on page 3 in Main Text):

"A fundamental role of macrophages is to selectively remove unwanted materials in their local microenvironment for proper maintenance of tissue homeostasis (Lemke, 2019). A conserved mechanism governing the selectivity of macrophage removal involves recognition of externalized phosphatidylserine (ePtdSer). PtdSer is normally asymmetrically localized to the inner leaflet of plasma membranes however, it can be externalized to the outer leaflet during caspase-mediated apoptosis for the removal by phagocytes (Segawa et al., 2014). Caspase-3 activation and PtdSer externalization can occur locally on isolated cell membranes in the absence of overt cell death (Ertürk, Wang and Sheng, 2014; Scott-Hewitt et al., 2020). Microglia express a plethora of receptors that recognize ePtdSer including AD-risk associated triggering receptor expressed on myeloid cells 2 (TREM2) (Wang et al., 2015). Here we hypothesized that local synaptic ePtdSer underlies vulnerability of synapses to microglial engulfment via TREM2".

Together with the additional text linking C1q, ePtdSer and TREM2 (in point **1)**, we hope there is now more clarity in our manuscript.

**3**) They adopted a clever experimental design whereby they demonstrated that spines exhibiting ePtdSer had greater calcium transient activity and are preferentially targeted for phagocytic clearance with a consequent reduction in calcium activity. Does complement play any role in this sequence of events - as might be expected from the literature and their previous work?

We thank the Reviewer for their comment. Based on published literature and our findings here, we can speculate on the sequence of events to propose the following model. Our data suggests that Aβ oligomers induce neuronal hyperactivity, which subsequently leads to local ePtdSer on dendritic spines. Furthermore, our data suggest that microglia selectively recognize the ePtdSer-positive spines via TREM2, which leads to microglial C1q reactivation and therefore synapse engulfment and synapse loss as we and others have previously shown (Hong et al., Science 2016, doi: 10.1126/science.aad8373, Dejanovic et al., Neuron 2018, doi: 10.1016/j.neuron.2018.10.014). Failure to remove these ePtdSer⁺ postsynaptic elements results in neuronal hyperactivity, suggesting a protective role for microglia in this process. We have condensed this sequence of events as part of the discussion linking TREM2/ePtdSer and C1q (detailed in point **1**).

**4**) Is the ability of oAb to provoke the appearance of ePtsSer on synaptosomes a unique property of Ab or will other stimuli elicit the same effect, e.g. other agents that drive Ca transients and depolarization? Is the effect of oAb on PtdSer externalization reliant upon the Ca transients?

Previous works in the developing brain altogether implicate synaptic ePtdSer in microglial-mediated synapse pruning (Scott-Hewitt et al., EMBO 2020, doi: 10.15252/embj.2020105380; Park et al., EMBO 2021, doi: 10.15252/embj.2020107121; Li et al., EMBO 2021, doi: 10.15252/embj.2021107915); all three studies are in context of brain development and do not involve additional challenge of Aβ oligomers. Hence, we do think the synaptic externalization of PtdSer as an 'eat me' signal by microglia is restricted to oligomeric Aβ, but that it may be a conserved mechanism to regulate specificity of engulfment throughout lifespan. To further address the Reviewer's question, however, especially regarding the potential reliance of $Ca^{2+}$ transients, we performed new experiments using *in vitro* primary neuronal cultures. We tested the effect of different concentrations of calcium in the buffer and KCl as a depolarising agent on ePtdSer upon Aβ challenge (please see Figure below; also added into the manuscript as **Fig S6**).

First, we examined whether ePtdSer relies on depolarization by high KCl treatment in normal or lower calcium concentration bath solution. Next, we also tested whether the externalization of PtdSer upon Aβ oligomer treatment is reliant on the calcium concentration. As expected, both high KCl treatment and Aβ oligomer treatment induced higher externalization of PtdSer in 2 mM calcium concentration compared to 0.2 mM. These results suggest that PtdSer externalization is dependent on calcium influx induced by either neuronal depolarization or Aβ oligomer and point in the direction that synaptic ePtdSer is not a unique property of Aβ oligomer but rather dependent on calcium influx that can be modulated by neuronal activity. We have also added more detail to the introduction on how and when PtdSer can be externalized (as described in point **2** above).

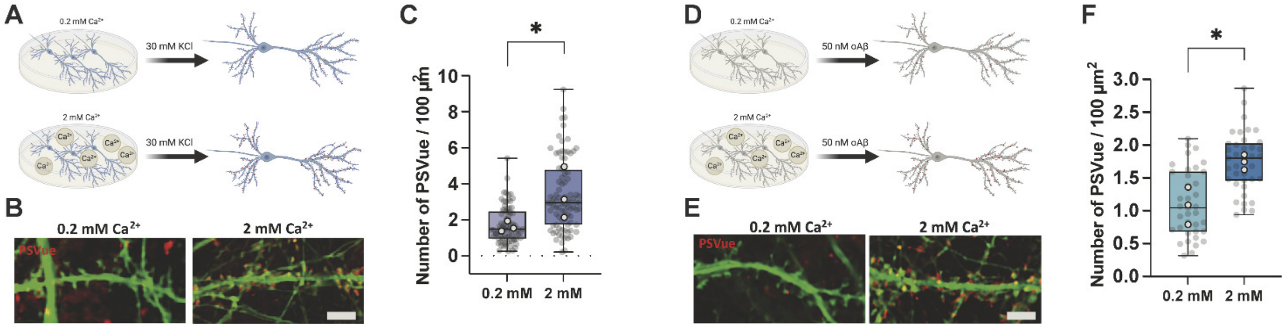

**5**) In Fig 3C/E they evaluated P2RY12+ microglia. Was the same elevation of lysosomal Homer+ puncta also elevated in TREM2+/CLEC7A+ , microglia (which should be found in the 6 mo mice) or is this a unique effect in the homeostatic microglia.

Our results here focus uniquely in early stages of amyloid pathology, i.e., 6 months old hAPP-NL-F KI mice before overt plaque deposition (please see Saito et al., Nat Neurosci 2014, doi: 10.1038/nn.3697); as such, we do not see any plaque-associated disease-associated microglia (DAM) cell clusters either using sc-RNA-seq or RNAScope/IHC (please also see De Schepper et al., Nat Neurosci 2023, doi: 10.1038/s41593-023-01257-z). This is as expected because various labs have shown that high *Clec7a* and low *Tmem119*/low *P2ry12* DAM appear at plaque stages in microglia that surround amyloid plaques (for e.g., Keren-Shaul et al., Cell 2017, doi: 10.1016/j.cell.2017.05.018; Krasemann et al., Immunity 2018, doi: 10.1016/j.immuni.2017.08.008; Sala Frigerio et al., 2019 Cell Rep, doi: 10.1016/j.celrep.2019.03.099; Chen et al., Cell 2020, doi: 10.1016/j.cell.2020.06.038; Wood et al., Cell Reports 2022, doi:10.1016/j.celrep.2022.111686). However, we performed additional experiments to confirm in these same mice for *Tmem119* (homeostatic gene) and *Clec7a* (DAM gene) expression using smFISH/RNAScope. As expected, we did not see any changes in *Tmem119* and *Clec7a* expression between the wild type and NL-F KI animals, which are both high and very low at these stages, respectively. We have added these data as **Fig S7B** and **C** in the revised manuscript.

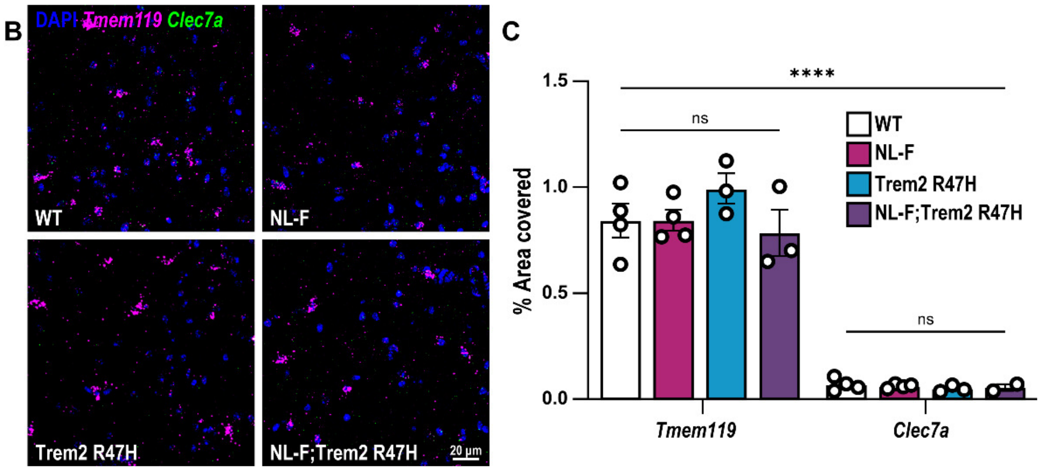


**6**) In Fig 4F/G the conclusion that TREM2 LOF variants leads to increased apoptotic synapses is based on examination of 2 individuals that are stated to carry TREM2 variants. The variants are not identified and it is unknown. whether they were similar in the two samples. I think it is inappropriate on this basis to conclude that overall brain active caspase 3 levels in synaptosomes are associated with the abundance of ePtdSer + synapses. These data should be removed from the manuscript.

We apologise for the confusion; we had originally detailed the LOF variants in Table S1. We have now made clearer in the Main Text where the human brain data are located. We also have increased the numbers of human brain samples now to: N=**9** for NDC, N=**11** for AD, N=**5** TREM2 NDC and N=**10** TREM2 AD. We understand that these numbers, in particular for TREM2 NDC, are still low; however, TREM2 NDC is rare and this is the maximum number we could obtain at the optimal brain conditions needed for our analysis from two major UK brain banks. Whereas we agree with the Reviewer that we should not overreach in our conclusions based on small sample size, there is a number of previous works on synaptic caspase 3 in AD (D'Amelio et al., Trends Neurosci, 2012, doi: 10.1016/j.tins.2012.06.004; Györffy et al., PNAS 2018, doi:10.1073/pnas.1722613115) and here, given our preclinical mouse data on TREM2, we hope that, now with the increased N and clarification,

the Reviewer agrees that this data can still be included in the manuscript. Please see amended **Fig 4H** and **Table S1**, which has all the patient information including the TREM2 variants. Please also see the amended text on line 5 on page 5 and on line 24 on page 11.

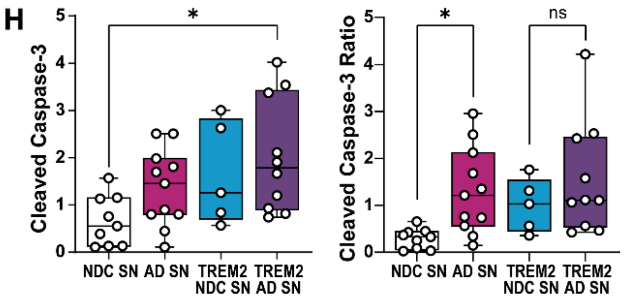


Minor Concerns:

**7**) The quality of the images provided in Figs 3E and S1D is poor. It is not clear whether this is due to incorporation of the panels into the pdf or that the original images also lack contrast.

We thank the Reviewer for pointing this out. We have now uploaded higher quality images. Please refer the amended **Figs 3E** (now **Fig 3G**) and **S1D** as well as the separate high-resolution document provided, which has the highest resolution images rather than the ones in the PDF document*.*

**Fig 3G**

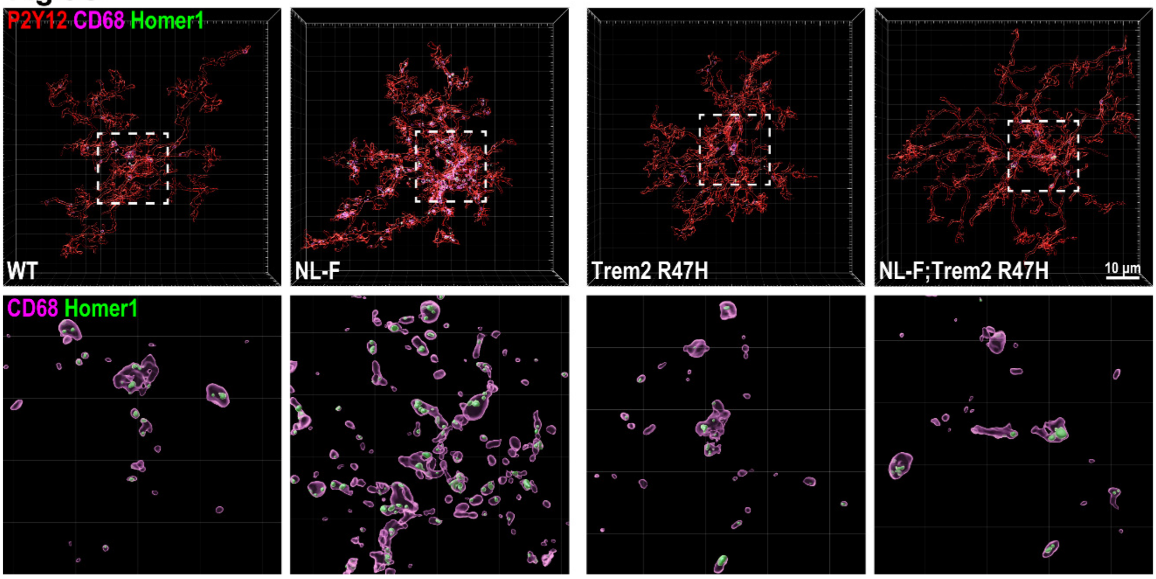


**Fig S1D**

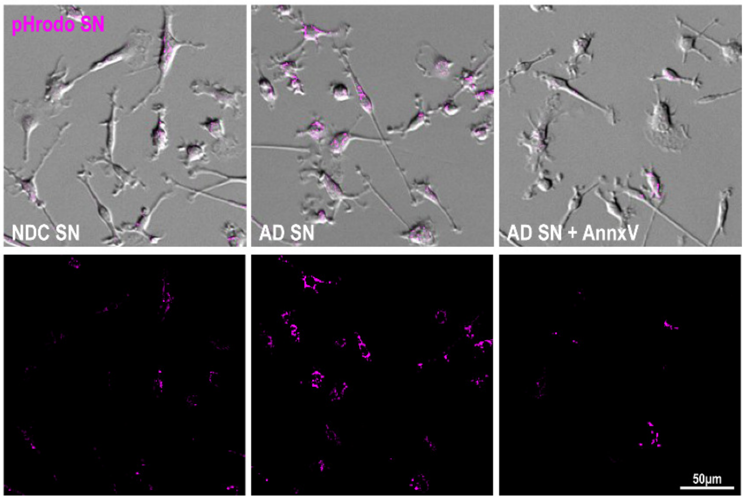

**8**) Fig S1 is problematic. There is a large variance in Ab levels between the AD samples shown. The standard curve in panel E appears to be non-linear, and compares poorly with a comparable blot in Fig S2 Panel D. There is no signal visible in the lower panels in the images provided to me. The image size is too small to resolve the cells in the upper panel.

We thank the Reviewer for pointing this out. We have now included revised western-blot which clearly demonstrates the presence of Aβ oligomers on isolated human AD synaptosomes. We have also increased the image size to resolve the cells better in both **Figs S1D**, please see above, and **S2C**. Please refer to the amended **Figs S1** and **S2C**.

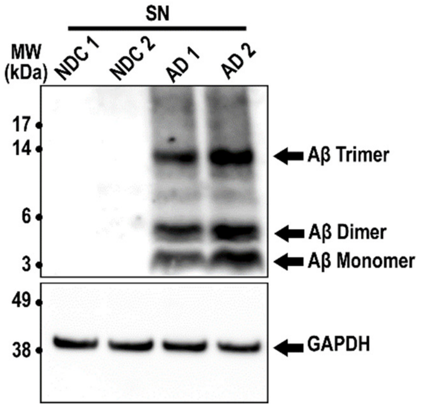


C

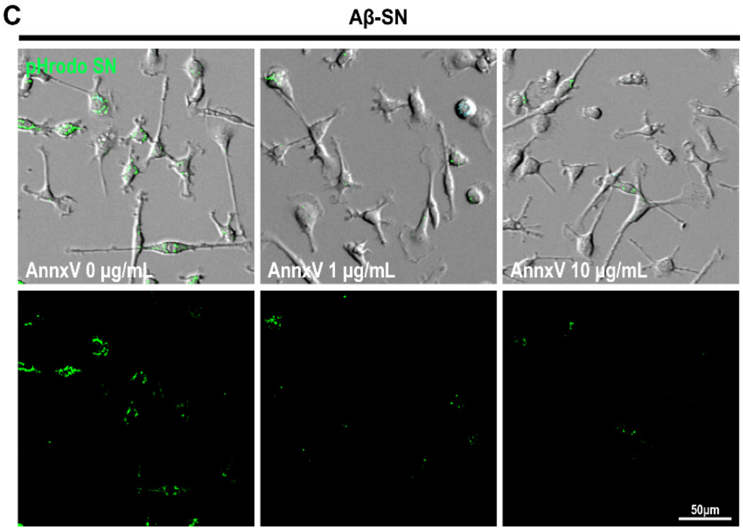

**Referee #2:**

In this manuscript, Rueda-Carrasco, Sokolova, and Lee et al. aim to show how microglia resolve neuronal hyperactivity by selectively engulfing damaged synapses in the earliest stages of Alzheimer's disease. Although synaptic loss induced by oligomeric Aß and synaptic engulfment by microglia occurs in mouse models of amyloidosis, the molecular mechanisms by which healthy microglia preferentially target synapses for removal in the pre-plaque-enriched AD brain are not well understood. Using a combination of super-resolution microscopy and live imaging approaches on in vitro co-cultures and in vivo genetic mouse models, the authors show that dendrites externalize phosphatidylserine (PtdSer) upon exposure to oligomeric Aß. They link microglia engulfment of PtdSer+ synapses in response to Aß exposure to TREM2 and aim to demonstrate this resolves Aß-induced neuronal hyperactivity. The work is largely compelling and answers a key question that is of broad interest to the general neuroscience and AD fields. There are two major and a few minor concerns that if addressed would greatly improve the impact of their findings and better support their conclusions.

We thank the Reviewer for this supporting comment.

Major Concerns:

**1**) Some of these images do not compellingly demonstrate the selective nature of elimination of hyperactive spines. While preferential engulfment of PSVue+ spines are striking in Figure 2A, contact and targeting appears non-discriminatory in Figure S5A perhaps because these still images do not illustrate the 3D nature of the data. As it is currently represented, there appear to be more PSVue- dendritic spines that are targeted while the microglia is homing to the PSVue+ spine. It may further emphasize the point to mark the hyperactive spines with an arrow in the movies. This applies to Figure S5A and Movie S2 & S3.

We thank the Reviewer for this important comment. We have taken these points into consideration and amended both the representation in **Fig S5A** as well as the accompanying supplementary movies. Please see the amended figures, which are now in 3D and hopefully demonstrate more clearly that the Homer1 spines that are targeted are PSVue$^{+}$. Please see the amended movies which now have arrows pointing to the hyperactive spines and the ones that are internalised which are PSVue$^{+}$. We also have modified the rendered image to change from transparent to solid when internalised into microglia to ease visualization. Please refer to the amended **Fig S5A** and **Movies S2** & **S3**.

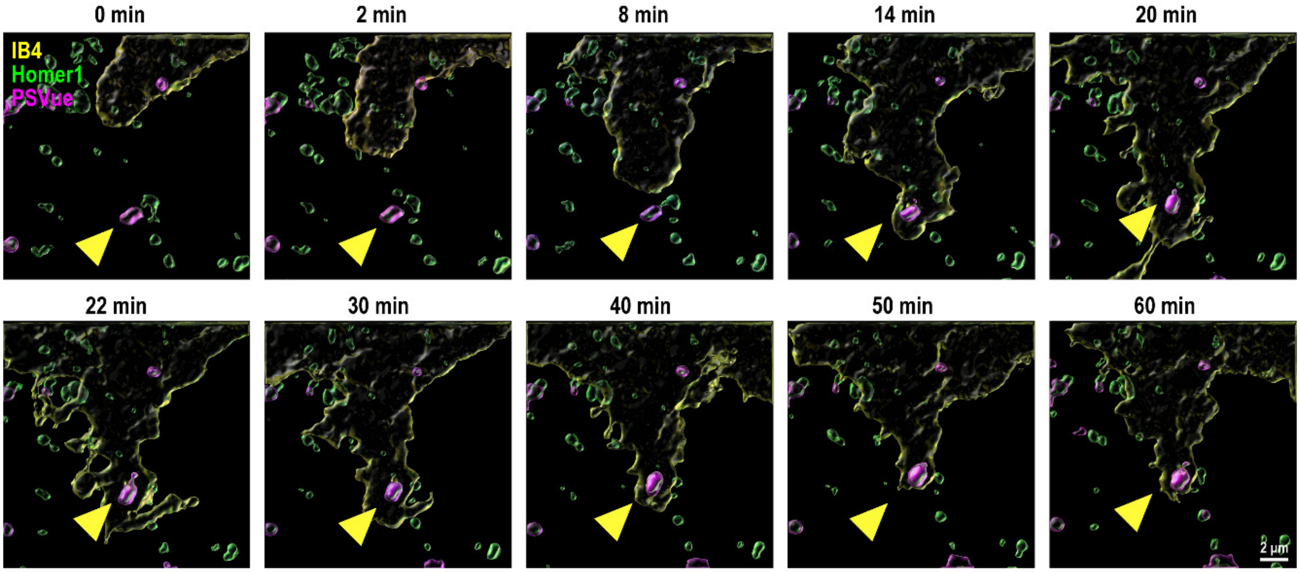

**2**) Collectively, the data presented in this paper implicate TREM2 in resolution of Aß-induced neuronal hyperactivity by mediating selective engulfment of Aß damaged, PtdSer+ synapses in microglia. While the in vitro co-culture and the in vivo Trem2 R47H KI mouse experiments in Figure 3 & 4 demonstrate the importance of TREM2 for preferential engulfment of PtdSer+ synapses, data linking TREM2 to resolution of neuronal hyperactivity is lacking.

a. Can primary Trem2 R47H KI microglia, when co-cultured with GCaMP7 transfected neurons, resolve neuronal hyperactivity 48 hours post oligomeric Aß challenge?
b. The gold standard for measuring hyperexcitability in this type of model is electrophysiologically. The killer experiment would be to perform slice physiological studies of the hippocampi of mice from the NL-F/TREM2 mice compared with controls to show that this axis is true in vivo. This is not strictly necessary but would go far to strengthen the conclusions.

We appreciate the Reviewer's comment and agree it is a critical point. Whereas the gold standard in the field of electrophysiology is to patch clamp individual cells *ex vivo* in acute slices, the main issue is that we are investigating the very early stages of the disease, and the percentage of affected synapses will be very low (1.5 and 4.8%, in NL-F and Trem2;NL-F brains, respectively, **Fig 4A** and **B**) to have a greater impact on the global neuron action potential. Hence we assessed instead for *in vitro* models, where we were able to assess individual synapses. Therefore, per Reviewer's advice (point **a**), we have now performed new co-culture experiments in order to link TREM2 with the resolution of Aβ oligomer induced neuronal hyperactivity. We found that whereas WT microglia can rescue neuronal hyperactivity, microglia cultured from Trem2 R47H KI mice fail to do so and neuronal hyperactivity persists at 48 h. This effect is similar to what we see when we mask ePtdSer with AnnxV. Therefore, this new data suggests that functional TREM2 is necessary for the resolution of neuronal hyperactivity. Please see amended **Fig 4D**.

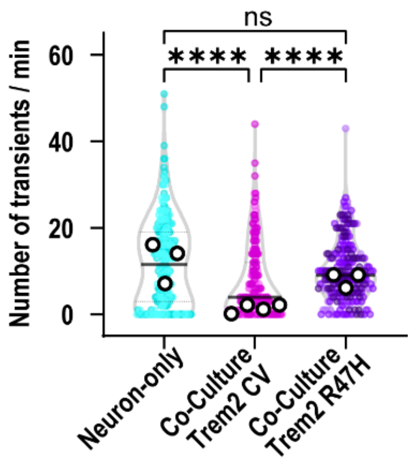


Minor Concerns:

**3**) There is some concern for pseudoreplication. Please highlight how each N was considered for statistical tests for Figure 1, 4, S2, S3, S5. For statistics in Figure S5, how were significant tests done, as an N as each ROI or per each biological N?

We apologise for the lack of clarity. We have now modified the graphs and clarified the text in the figure legends to clearly indicate how the significant tests were done and what the N is. The significance of the statistical tests was performed by biological replicates and the independent acquisitions are plotted just to depict the intra-replica variability. We have included a more detailed description in the Statistics subheading of the Materials and Methods section, please refer to the

amended text on line 35 on page 26. Please see the amended figures and their respective figure legends in the text.

In particular for **Fig S5**, we conducted a statistical analysis employing a Kruskal-Wallis test complemented by Dunn's multiple comparisons test due to the non-normal distribution of the data. The revised violin plot now incorporates the display of the median and frequency distribution, and the median values of each independent experiment are depicted as open circles within the plots. The statistical tests for GCaMP experiments were conducted on a per-individual-spine basis, in accordance with the field's standards.

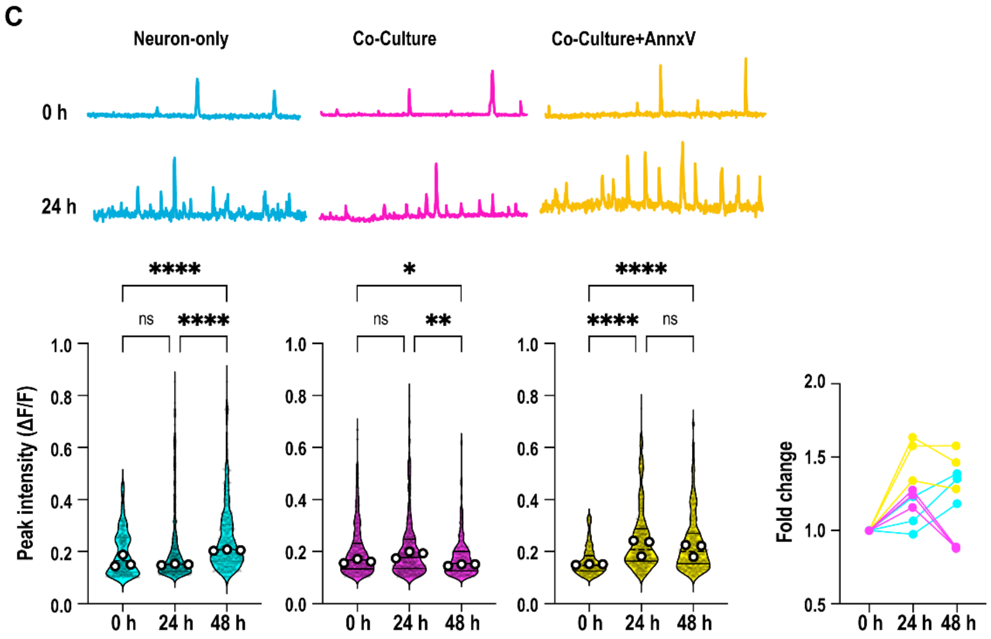


**4**) Recommend in the abstract/introduction replacing the term "apoptotic" synapses with "synaptotic" or "apoptotic-like" synapses consistently for clarity.

We agree with the Reviewer. We have now modified the text throughout the manuscript and replaced "apoptotic" synapses with "apoptotic-like" synapses for clarity as suggested.

Formatting:

**5**) There are a few typos populated throughout the text (full stop on page 7 line 25, GCaMP7 on page 15 line 33, hours on page 35 line 4, Synaptotagmin on page 36 line 8)

We thank the Reviewer for pointing this out. We have amended these and any other typos.

**6**) Please introduce reagents (Annexin V, PSVue, etc.) as they first appear in the text (i.e. background statement on Annexin V appears on page 8 even though it first appeared on page 5)

We have amended this and other inaccuracies in the text.

**7**) Please add arrowheads to highlight a few representative PtdSer+ vs. PtdSer- spines in Movie S5.

We have now added arrowheads to highlight the spines of relevance in the videos. Please refer to the amended videos.

**8**) Movies are currently mislabeled (i.e. Movie S1 was labeled and uploaded as Movie 3)

We have now amended the labels.

## 1st Revision - Editorial Decision 25th Jun 2023

Dear Soyon,

Thank you for submitting your revised manuscript to The EMBO Journal. Your study has now been seen by the two original referees and they comments are provided below. As you can see from the comments the referees appreciate the introduced changes and support publication here.

Referee #1 still has some hesitations regarding the findings in figure 4. Can you make sure that you clearly state in the text which results are significant and which ones are not.

When you submit the revised version will you also take care of the following editorial points:

- Please upload a MS file without figures and high-quality individual figure files.

- The funding info should be provided as part of the Ackn section.

- We need a Data Availability section. This is the place to enter accession numbers etc. If no data is generated that needs to be deposited in a database then please state: Data Availability: This study includes no data deposited in external repositories.

- COI: should be merged under "Disclosure and competing interests statement".

- Please remove the Authors Contributions from the manuscript. The 'Author Contributions' section is replaced by the CRediT contributor roles taxonomy to specify the contributions of each author in the journal submission system. Please use the free text box in the 'author information' section of the manuscript submisssion system to provide more detailed descriptions (e.g., 'X provided intracellular Ca++ measurements in fig Y').

- Please make sure that there are figure callouts for Fig.S5C, Fig S6 and Fig S8 panels.

- Please order the sections of the MS: abstract, introduction, results, discussion, materials & methods, data availability section, acknowledgments, disclosure statement and competing interests, references, main figure legends, tables, expanded figure legends.

- The movie files be renamed to Movie EV1, etc. and each movie zipped together with its corresponding legend; the movie legends should be removed from the MS and the callouts updated.

- The Supplementary information (figures, legends, additional text) should be removed from the MS and placed in an Appendix file that has a ToC with page numbers; the nomenclature should be Appendix Figure S1, etc. callouts in the MS text should be updated accordingly.

- Table S1 should be renamed to Table EV1 and uploaded as Dataset. Please correct callout in text.

- Our publisher has also done their pre-publication check on your manuscript. When you log into the manuscript submission system you will see the file "Data Edited Manuscript file". Please take a look at the word file and the comments regarding the figure legends and respond to the issues.

- Please upload a synopsis text. We need a summary statement plus 3-5 bullet points describing the key findings of the MS.

- We also need a synopsis image => 550 wide by [200-400].

- Please also provide source data - see email from Hannah Sonntag from the 25th of Jan 23.

That should be all - let us know if you have any further questions.

With best wishes

Karin

Karin Dumstrei, PhD
Senior Editor
The EMBO Journal

Guide For Authors: https://www.embopress.org/page/journal/14602075/authorguide

------------------------------------------------

Referee #1:

The authors have responded appropriately, providing revised text and some new data. I raised questions about Figure 4 and they have now obtained more samples, but as they note, the n's are still small. I remain unconvinced and the key comparisons are not significant.

Referee #2:

The authors have addressed all my concerns. Thank you for this important study!

Dear Dr. Dumstrei,

Thank you for giving us the opportunity to respond additional comments from you and the referees. We have since performed additional edits regarding the main concerns and revised accordingly. Below, please find our point-by-point responses.

We would like to thank the reviewers for their supporting comments and positive reception of our work

**Referee #1:**

The authors have responded appropriately, providing revised text and some new data. I raised questions about Figure 4 and they have now obtained more samples, but as they note, the n's are still small. I remain unconvinced and the key comparisons are not significant.

We thank the Reviewer for the recognition of our efforts. We appreciate the referee's suggestion and have revised the manuscript to tune down our conclusion regarding the human translatability of our findings, making clear which results are significant and which ones are not (line 24 on page 11 in Main Text). Also modifying accordingly the **Fig 4H** to represent all the significant comparisons and not reporting the non-significant ones.

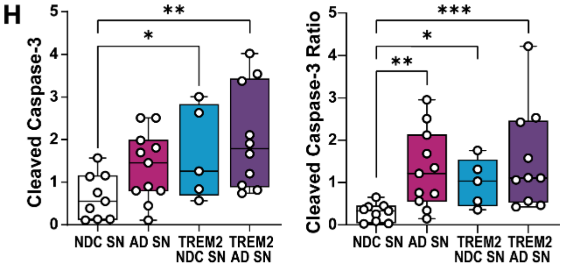


"Finally, we assessed the translational relevance of ePtdSer$^{+}$ synapses in patient brains. Direct PSVue labeling is not possible in frozen post-mortem samples. However, PtdSer is canonically externalized by the cleavage of caspase 3 (Ertürk *et al*, 2014; Segawa *et al*, 2014) as such, we probed for levels of full length and cleaved caspase-3 by western-blotting on isolated human synaptosomes. We found increased levels of caspase-3 ratio in synaptosomes isolated from human AD brains as compared to those from NDC brains (**Fig 4G** and **H;** N=9 for NDC, N=11 for AD, N=5 TREM2 NDC and N=10 TREM2 AD)**.** Due to limited availability of TREM2 patient tissue we were only able to sample a small cohort of patients carrying AD-risk associated TREM2 variants. In this cohort, we found significantly higher levels of caspase-3 activation in TREM2 AD synaptosomes compared to NDC and similarly when comparing synaptosomes derived from patients without pathology, between TREM2 NDC and NDC (**Fig 4G** and **H**). These data may suggest that TREM2 loss-of-function may impact human brains likewise to what we observed in mouse models, but larger cohort analysis is needed to better elucidate the relationship in between TREM2 function and apoptotic-like synapses in humans."

**Referee #2:**

The authors have addressed all my concerns. Thank you for this important study!

We thank the Reviewer for the recognition of our efforts.

**Editorial points**

- Please upload a MS file without figures and high-quality individual figure files.

This has been done.

- The funding info should be provided as part of the Ackn section.

This has been done.

- We need a Data Availability section. This is the place to enter accession numbers etc. If no data is generated that needs to be deposited in a database then please state: Data Availability: This study includes no data deposited in external repositories.

This statement has been included.

- COI: should be merged under "Disclosure and competing interests statement".

These sections have been merged.

- Please remove the Authors Contributions from the manuscript. The 'Author Contributions' section is replaced by the CRediT contributor roles taxonomy to specify the contributions of each author in the journal submission system. Please use the free text box in the 'author information' section of the manuscript submission system to provide more detailed descriptions (e.g., 'X provided intracellular Ca++ measurements in fig Y').

This has been removed.

- Please make sure that there are figure callouts for Fig.S5C, Fig S6 and Fig S8 panels.

This has been included.

- Please order the sections of the MS: abstract, introduction, results, discussion, materials & methods, data availability section, acknowledgments, disclosure statement and competing interests, references, main figure legends, tables, expanded figure legends.

This has been done.

- The movie files be renamed to Movie EV1, etc. and each movie zipped together with its corresponding legend; the movie legends should be removed from the MS and the callouts updated.

This has been done.

- The Supplementary information (figures, legends, additional text) should be removed from the MS and placed in an Appendix file that has a ToC with page numbers; the nomenclature should be Appendix Figure S1, etc. callouts in the MS text should be updated accordingly.

This has been done.

- Table S1 should be renamed to Table EV1 and uploaded as Dataset. Please correct callout in text.

This has been done.

- Our publisher has also done their pre-publication check on your manuscript. When you log into the manuscript submission system you will see the file "Data Edited Manuscript file". Please take a look at the word file and the comments regarding the figure legends and respond to the issues.

This has been amended.

- Please upload a synopsis text. We need a summary statement plus 3-5 bullet points describing the key findings of the MS.

This has been uploaded.

“Early stages of Alzheimer’s disease (AD) are characterised by neuronal hyperactivity. Aβ oligomers induce synapses to become hyperactive and externalize phosphatidylserine (ePtdSer), a canonical ‘eat-me’ signal. Functional TREM2 is crucial for microglia to detect and preferentially engulf ePtdSer$^{+}$ synapses and help ameliorate neuronal hyperactivity.

- Aβ oligomers induce synapses to externalize PtdSer.

- ePtdSer$^{+}$ spines have higher spontaneous $Ca^{2+}$ signals.

- Microglia engulf ePtdSer$^{+}$ hyperactive synapses via TREM2, resulting in the resolution of neuronal hyperactivity.

- TREM2 loss-of-function results in disruption of microglial removal of ePtdSer$^{+}$ synapses and sustained neuronal hyperactivity.”

- We also need a synopsis image => 550 wide by [200-400].

This has been uploaded.

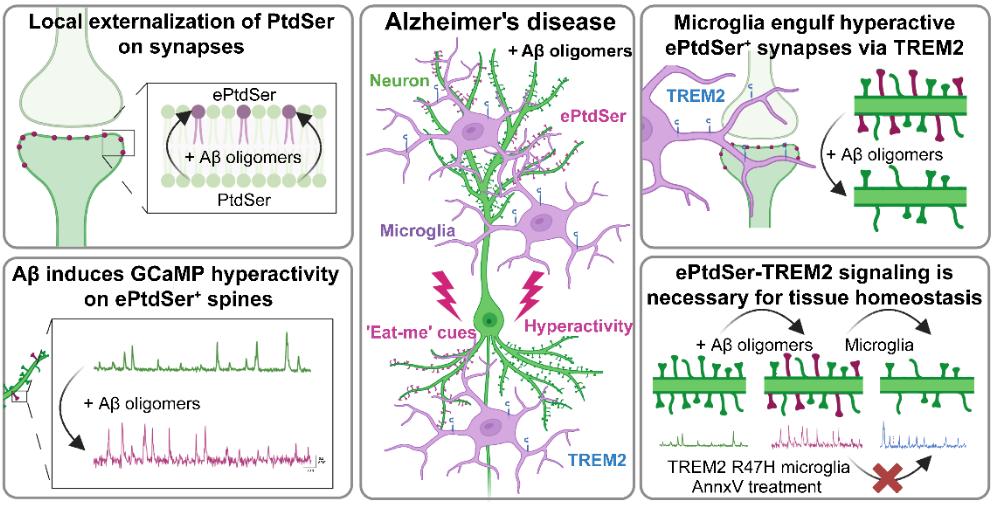


- Please also provide source data - see email from Hannah Sonntag from the 25th of Jan 23.

This has been provided.

**2nd Revision - Editorial Decision** **25th Jul 2023**

Dear Soyon,

Thank you for submitting your revised manuscript to The EMBO Journal. I have now had a chance to take a careful look at it and all looks good.

I am therefore very pleased to accept the manuscript for publication here.

with best wishes

Karin

Karin Dumstrei, PhD
Senior Editor
The EMBO Journal

------------------------------------------------

Please note that it is EMBO Journal policy for the transcript of the editorial process (containing referee reports and your response letter) to be published as an online supplement to each paper. If you do NOT want this, you will need to inform the Editorial Office via email immediately. More information is available here:
https://www.embopress.org/page/journal/14602075/authorguide#transparentprocess

Your manuscript will be processed for publication in the journal by EMBO Press. Manuscripts in the PDF and electronic editions of The EMBO Journal will be copy edited, and you will be provided with page proofs prior to publication. Please note that supplementary information is not included in the proofs.

You will be contacted by Wiley Author Services to complete licensing and payment information. The required 'Page Charges Authorization Form' is available here: https://www.embopress.org/pb-assets/embo-site/tej_apc.pdf - please download and complete the form and return to embopressproduction@wiley.com

EMBO Press participates in many Publish and Read agreements that allow authors to publish Open Access with reduced/no publication charges. Check your eligibility: https://authorservices.wiley.com/author-resources/Journal-Authors/open-access/affiliation-policies-payments/index.html

Should you be planning a Press Release on your article, please get in contact with embojournal@wiley.com as early as possible, in order to coordinate publication and release dates.

If you have any questions, please do not hesitate to call or email the Editorial Office. Thank you for your contribution to The EMBO Journal.

## EMBO Press Author Checklist

| | |
|---|---|
| Corresponding Author Name: Soyon Hong | |
| Journal Submitted to: The EMBO Journal | |
| Manuscript Number: EMBOJ-2022-113246R | |

**USEFUL LINKS FOR COMPLETING THIS FORM**
The EMBO Journal - Author Guidelines
EMBO Reports - Author Guidelines
Molecular Systems Biology - Author Guidelines
EMBO Molecular Medicine - Author Guidelines

**Reporting Checklist for Life Science Articles (updated January**
This checklist is adapted from Materials Design Analysis Reporting (MDAR) Checklist for Authors. MDAR establishes a minimum set of requirements in transparent reporting in the life sciences (see Statement of Task: 10.31222/osf.io/9sm4x). Please follow the journal's guidelines in preparing your manuscript.
**Please note that a copy of this checklist will be published alongside your article.**

**Abridged guidelines for figures**
**1. Data**
The data shown in figures should satisfy the following conditions:
- the data were obtained and processed according to the field's best practice and are presented to reflect the results of the experiments in an accurate and unbiased manner.
- ideally, figure panels should include only measurements that are directly comparable to each other and obtained with the same assay.
- plots include clearly labeled error bars for independent experiments and sample sizes. Unless justified, error bars should not be shown for technical
- if n<5, the individual data points from each experiment should be plotted. Any statistical test employed should be justified.
- Source Data should be included to report the data underlying figures according to the guidelines set out in the authorship guidelines on Data

**2. Captions**
Each figure caption should contain the following information, for each panel where they are relevant:
- a specification of the experimental system investigated (eg cell line, species name).
- the assay(s) and method(s) used to carry out the reported observations and measurements.
- an explicit mention of the biological and chemical entity(ies) that are being measured.
- an explicit mention of the biological and chemical entity(ies) that are altered/varied/perturbed in a controlled manner.
- the exact sample size (n) for each experimental group/condition, given as a number, not a range;
- a description of the sample collection allowing the reader to understand whether the samples represent technical or biological replicates (including how many animals, litters, cultures, etc.).
- a statement of how many times the experiment shown was independently replicated in the laboratory.
- definitions of statistical methods and measures:
  - common tests, such as t-test (please specify whether paired vs. unpaired), simple χ2 tests, Wilcoxon and Mann-Whitney tests, can be unambiguously identified by name only, but more complex techniques should be described in the methods section;
  - are tests one-sided or two-sided?
  - are there adjustments for multiple comparisons?
  - exact statistical test results, e.g., P values = x but not P values < x;
  - definition of 'center values' as median or average;
  - definition of error bars as s.d. or s.e.m.

**Please complete ALL of the questions below.**
**Select "Not Applicable" only when the requested information is not relevant for your study.**

**Materials**

| **Newly Created Materials** | **Information included in the manuscript?** | **In which section is the information available?** (Reagents and Tools Table, Materials and Methods, Figures, Data Availability Section) |
|---|---|---|
| New materials and reagents need to be available; do any restrictions apply? | Not Applicable | |

| **Antibodies** | **Information included in the manuscript?** | **In which section is the information available?** (Reagents and Tools Table, Materials and Methods, Figures, Data Availability Section) |
|---|---|---|
| For **antibodies** provide the following information:<br>- Commercial antibodies: RRID (if possible) or supplier name, catalogue number and or/clone number<br>- Non-commercial: RRID or citation | Yes | Materials and Methods, Antibodies Information Section |

| **DNA and RNA sequences** | **Information included in the manuscript?** | **In which section is the information available?** (Reagents and Tools Table, Materials and Methods, Figures, Data Availability Section) |
|---|---|---|
| **Short novel DNA or RNA including primers, probes**: provide the sequences. | Yes | Materials and Methods, Primers sequences Section |

| **Cell materials** | **Information included in the manuscript?** | **In which section is the information available?** (Reagents and Tools Table, Materials and Methods, Figures, Data Availability Section) |
|---|---|---|
| **Cell lines:** Provide species information, strain. Provide accession number in repository **OR** supplier name, catalog number, clone number, and/**OR** RRID. | Not Applicable | |
| **Primary cultures:** Provide species, strain, sex of origin, genetic modification status. | Yes | Materials and Methods, Animals Section |
| Report if the cell lines were recently **authenticated** (e.g., by STR profiling) and tested for mycoplasma contamination. | Not Applicable | |

| **Experimental animals** | **Information included in the manuscript?** | **In which section is the information available?** (Reagents and Tools Table, Materials and Methods, Figures, Data Availability Section) |
|---|---|---|
| **Laboratory animals or Model organisms:** Provide species, strain, sex, age, genetic modification status. Provide accession number in repository **OR** supplier name, catalog number, clone number, **OR** RRID. | Yes | Materials and Methods, Animals Section |
| **Animal observed in or captured from the field:** Provide species, sex, and age where possible. | Not Applicable | |
| Please detail **housing and husbandry conditions**. | Yes | Materials and Methods, Animals Section |

| **Plants and microbes** | **Information included in the manuscript?** | **In which section is the information available?** (Reagents and Tools Table, Materials and Methods, Figures, Data Availability Section) |
|---|---|---|
| **Plants:** provide species and strain, ecotype and cultivar where relevant, unique accession number if available, and source (including location for collected wild specimens). | Not Applicable | |
| **Microbes:** provide species and strain, unique accession number if available, and source. | Not Applicable | |

| **Human research participants** | **Information included in the manuscript?** | **In which section is the information available?** (Reagents and Tools Table, Materials and Methods, Figures, Data Availability Section) |
|---|---|---|
| If collected and within the bounds of privacy constraints report on age, sex and gender or ethnicity for all study participants. | Yes | Table S1 |

| **Core facilities** | **Information included in the manuscript?** | **In which section is the information available?** (Reagents and Tools Table, Materials and Methods, Figures, Data Availability Section) |
|---|---|---|
| If your work benefited from core facilities, was their service mentioned in the acknowledgments section? | Yes | Acknowledgements |

**Design**

| Study protocol | Information included in the manuscript? | In which section is the information available? (Reagents and Tools Table, Materials and Methods, Figures, Data Availability Section) |
|---|---|---|
| If study protocol has been **pre-registered, provide DOI in the manuscript**. For clinical trials, provide the trial registration number **OR** cite DOI. | Not Applicable | |
| Report the **clinical trial registration number** (at ClinicalTrials.gov or equivalent), where applicable. | Not Applicable | |

| Laboratory protocol | Information included in the manuscript? | In which section is the information available? (Reagents and Tools Table, Materials and Methods, Figures, Data Availability Section) |
|---|---|---|
| Provide DOI OR other citation details if **external detailed step-by-step protocols** are available. | Not Applicable | |

| Experimental study design and statistics | Information included in the manuscript? | In which section is the information available? (Reagents and Tools Table, Materials and Methods, Figures, Data Availability Section) |
|---|---|---|
| Include a statement about **sample size** estimate even if no statistical methods were used. | Yes | Figure legends |
| Were any steps taken to minimize the effects of subjective bias when allocating animals/samples to treatment (e.g. **randomization procedure**)? If yes, have they been described? | Yes | Materials and Methods |
| Include a statement about **blinding** even if no blinding was done. | Yes | Materials and Methods |
| Describe **inclusion/exclusion criteria** if samples or animals were excluded from the analysis. Were the criteria pre-established?<br><br>If sample or data points were omitted from analysis, report if this was due to attrition or intentional exclusion and provide justification. | Yes | Materials and Methods |
| For every figure, are **statistical tests** justified as appropriate? Do the data meet the assumptions of the tests (e.g., normal distribution)? Describe any methods used to assess it. Is there an estimate of variation within each group of data? Is the variance similar between the groups that are being statistically compared? | Yes | Figure legends |

| Sample definition and in-laboratory replication | Information included in the manuscript? | In which section is the information available? (Reagents and Tools Table, Materials and Methods, Figures, Data Availability Section) |
|---|---|---|
| In the figure legends: state number of times the experiment was **replicated** in laboratory. | Yes | Figure legends |
| In the figure legends: define whether data describe **technical or biological replicates**. | Yes | Figure legends |

## Ethics

| Ethics | Information included in the manuscript? | In which section is the information available? (Reagents and Tools Table, Materials and Methods, Figures, Data Availability Section) |
|---|---|---|
| Studies involving **human participants**: State details of **authority granting ethics approval** (IRB or equivalent committee(s), provide reference number for approval. | Yes | Licences |
| Studies involving **human participants**: Include a statement confirming that **informed consent** was obtained from all subjects and that the experiments conformed to the principles set out in the WMA Declaration of Helsinki and the Department of Health and Human Services Belmont Report. | Not Applicable | |
| Studies involving **human participants:** For publication of **patient photos**, include a statement confirming that consent to publish was obtained. | Not Applicable | |
| Studies involving experimental **animals**: State details of **authority granting ethics approval** (IRB or equivalent committee(s), provide reference number for approval. Include a statement of compliance with ethical regulations. | Yes | Licences |
| Studies involving **specimen and field samples:** State if relevant **permits** obtained, provide details of authority approving study; if none were required, explain why. | Not Applicable | |

| Dual Use Research of Concern (DURC) | Information included in the manuscript? | In which section is the information available? (Reagents and Tools Table, Materials and Methods, Figures, Data Availability Section) |
|---|---|---|
| Could your study fall under dual use research restrictions? Please check biosecurity documents and list of **select agents and toxins** (CDC): https://www.selectagents.gov/sat/list.htm | Not Applicable | |
| If you used a select agent, is the security level of the lab appropriate and reported in the manuscript? | Not Applicable | |
| If a study is subject to dual use research of concern regulations, is the name of the **authority granting approval and reference number** for the regulatory approval provided in the manuscript? | Not Applicable | |

## Reporting

The MDAR framework recommends adoption of discipline-specific guidelines, established and endorsed through community initiatives. Journals have their own policy about requiring specific guidelines and recommendations to complement MDAR.

| Adherence to community standards | Information included in the manuscript? | In which section is the information available? (Reagents and Tools Table, Materials and Methods, Figures, Data Availability Section) |
|---|---|---|
| State if relevant guidelines or checklists (e.g., **ICMJE, MIBBI, ARRIVE, PRISMA**) have been followed or provided. | Not Applicable | |
| For **tumor marker prognostic studies,** we recommend that you follow the **REMARK** reporting guidelines (see link list at top right). See author guidelines, under 'Reporting Guidelines'. Please confirm you have followed these guidelines. | Not Applicable | |
| For **phase II and III randomized controlled trials**, please refer to the **CONSORT** flow diagram (see link list at top right) and submit the CONSORT checklist (see link list at top right) with your submission. See author guidelines, under 'Reporting Guidelines'. Please confirm you have submitted this list. | Not Applicable | |

## Data Availability

| Data availability | Information included in the manuscript? | In which section is the information available? (Reagents and Tools Table, Materials and Methods, Figures, Data Availability Section) |
|---|---|---|
| Have **primary datasets** been deposited according to the journal's guidelines (see 'Data Deposition' section) and the respective accession numbers provided in the Data Availability Section? | Not Applicable | |
| Were **human clinical and genomic datasets** deposited in a public access-controlled repository in accordance to ethical obligations to the patients and to the applicable consent agreement? | Not Applicable | |
| Are **computational models** that are central and integral to a study available without restrictions in a machine-readable form? Were the relevant accession numbers or links provided? | Not Applicable | |
| If publicly available data were reused, provide the respective **data citations in the reference list**. | Not Applicable | |
